# Patterns in the bony skull development of marsupials: high variation in onset of ossification and conserved regions of bone contact

**DOI:** 10.1038/srep43197

**Published:** 2017-02-24

**Authors:** Stephan N. F. Spiekman, Ingmar Werneburg

**Affiliations:** 1 Paläontologisches Institut und Museum der Universität Zürich, Karl-Schmid-Strasse 4, 8006 Zürich, Switzerland; 2 Institute of Biology Leiden (IBL) at Leiden University, Sylviusweg 72, 2333 BE Leiden, the Netherlands; 3 Museum für Naturkunde, Leibniz-Institut für Evolutions- and Biodiversitätsforschung an der Humboldt-Universität zu Berlin, Invalidenstraße 43, 10115 Berlin, Germany; 4 Senckenberg Center for Human Evolution and Palaeoenvironment (HEP) at Eberhard Karls Universität, Sigwartstraße 10, 72074 Tübingen, Germany; 5 Eberhard Karls Universität, Hölderlinstraße 12, room: 308g, D-72076 Tübingen, Germany

## Abstract

Development in marsupials is specialized towards an extremely short gestation and highly altricial newborns. As a result, marsupial neonates display morphological adaptations at birth related to functional constraints. However, little is known about the variability of marsupial skull development and its relation to morphological diversity. We studied bony skull development in five marsupial species. The relative timing of the onset of ossification was compared to literature data and the ossification sequence of the marsupial ancestor was reconstructed using squared-change parsimony. The high range of variation in the onset of ossification meant that no patterns could be observed that differentiate species. This finding challenges traditional studies concentrating on the onset of ossification as a marker for phylogeny or as a functional proxy. Our study presents observations on the developmental timing of cranial bone-to-bone contacts and their evolutionary implications. Although certain bone contacts display high levels of variation, connections of early and late development are quite conserved and informative. Bones that surround the oral cavity are generally the first to connect and the bones of the occipital region are among the last. We conclude that bone contact is preferable over onset of ossification for studying cranial bone development.

Marsupials are born at the most altricial (premature) stage of all mammals. A large part of the development that occurs embryonically in other mammals takes place after birth in marsupials, making them a very important group for developmental studies because of the opportunity to observe these later stages *in vivo* and ex utero^1^,^2^,^3^. With the increased interest in constraints for studies in evolutionary biology^4^, marsupial development has been extensively studied in light of the marsupial-placental dichotomy^5^, with a particular focus on the presence^6^,^7^ or absence^8^ of developmental constraints. Very little is known, however, about patterns of development within the marsupial clade and their relation to marsupial morphological diversity.

In addition to the physiological adaptations attributed to the unique life history strategy of marsupials, most adaptations of the marsupial neonate relate to the journey from the vagina into the maternal pouch and the subsequent attachment to the teat immediately after birth, affecting both cranial and postcranial morphology. The postcranium is most notably effected by the neonate’s climb into the pouch, which is unaided by the mother in many species^9^. In order to make this journey at such a highly altricial state, marsupials have well-developed forelimbs. Conversely, development of the hind limbs is delayed^10^,^11^,^12^,^13^. These functional requirements of the forelimbs to reach the teat have been shown to constrain variability in forelimb morphotypes^14^. This constraint has often been used to explain why marsupials do not show the high forelimb disparity as seen in placentals. Placental forelimbs have evolved into specialized structures such as wings (in chiropterans) and flippers (in cetaceans, sirenians, and pinnipeds). Marsupial forelimbs are much more conserved, although specialized adaptations in marsupials occur to some degree (e.g., the marsupial mole *Notoryctes*)^15^,^16^.

Cranial development is mainly affected by functional requirements of early life attached to the teat of the mother. After attachment, the oral region effectively fuses with the teat, which permits a stable position such that further development of the pouch young can occur. This fusion takes place through a swelling of the teat inside the mouth cavity and the bilateral fusion of the lips by a keratinized membrane called the epitrichium^11^. This membrane also covers the eyes and ears to protect these structures in the neonate until the epitrichium is lost later during development^1^. In order to suckle and breathe simultaneously, both the nasal cavity and tongue are strongly developed. A cervical swelling supports the head while being attached to the teat^11^,^17^. To facilitate these adaptations for suckling, the relative timing of the development of bones and musculature in the oral and facial region is accelerated, whereas the relative timing of the brain is delayed compared to placentals^18^,^19^,^20^. This earlier development of the oral region and the complex suckling behavior led to the consideration that this region is particularly constrained in marsupials. Recently, Goswami *et al*.^21^ quantified cranial ontogeny using phenotypic trajectory analysis to compare disparity in placental and marsupial cranial development with a special focus on the oral region. They showed that the development of the bones of this region is indeed constrained in marsupials compared to placentals. However, when considering the entire skull, both groups showed the same amount of cranial disparity.

Although bony skull development in marsupials has been studied in this comparative context among mammals, studies focusing on differences in cranial skeletogenesis within the marsupial clade never included more than two marsupial species^22^,^23^. Therefore, in order to further understand the cranial morphological disparity observed within this group, a detailed investigation of the variation of cranial development in a wide range of marsupials was desirable^21^,^24^.

The aim of our study was to quantify cranial development of marsupials by establishing 1) the relative timing of the onset of ossification for each bone and 2) the relative timing of each bone-to-bone contact. We considered bone contact, which has previously not been used as a quantitative metric in developmental biology, to be the most reliable proxy to indicate the presence of structurally robust and functionally important bones during skeletal development. Furthermore, since bone contact logically follows some time after the onset of ossification of the respective bones, the former provides information for more progressed developmental stages than the latter. The relative timing and its variation for both bone onset and bone contact could be compared between all skull elements, giving new quantitative insights into marsupial osteogenesis. Finally, by comparing these two different metrics, we were able to determine their general value for studying cranial development.

## Methods

### Specimens

**Figure 1:**
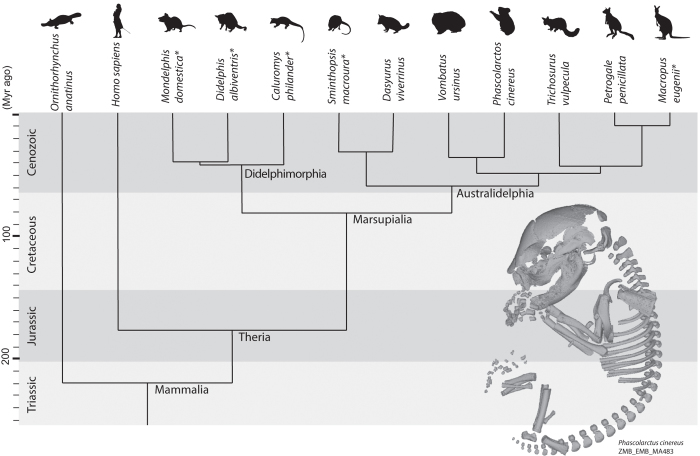
A scaled phylogeny of the marsupial species studied in the present study, as well as the species from Koyabu *et al*.^29^ that were included for the onset of ossification analysis, and two outgroup representatives, one placental (*Homo*) and one monotreme (*Ornithorhynchus*). The species for which all data were taken from Koyabu *et al*.^29^ are marked by an asterisk (*). Note however that for the onset of ossification analysis, data for *Dasyurus viverrinus* and *Trichosurus vulpecula* were taken from Koyabu *et al*.^29^. For *T. vulpecula* these data were used in addition to data collected directly for this study. In addition, the most relevant clade names are included. The phylogenetic tree is scaled following the divergence times used for the analyses in Mesquite.

In total, we studied 115 different pouch young in five marsupial species: the common brushtail possum (*Trichosurus vulpecula*, 35 specimens), the eastern quoll (*Dasyurus viverrinus*, 23 specimens), the koala (*Phascolarctos cinereus*, 30 specimens), the common wombat (*Vombatus ursinus*, 11 specimens), and the brush-tailed rock-wallaby (*Petrogale penicillata*, 16 specimens) (Fig. 1 and Suppl. Table 1). We have chosen these species out of all 41 marsupial species available in the Hill-Collection^25^, which is part of the Embryological collection of the Museum für Naturkunde Berlin, Germany^26^,^27^. The selected species are represented by a well distributed series of specimens at different relative levels of development, ranging from early pouch young at the beginning of cranial osteogenesis to adult specimens. The studied species represent a phylogenetically diverse subset of marsupials.

### μCT-scanning

**Figure 2:**
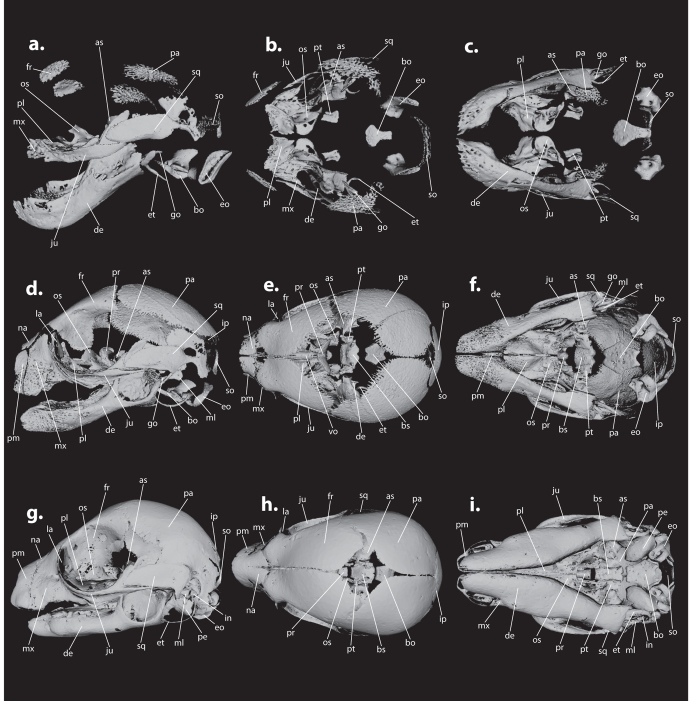
One plate with three different stages of cranial bone development in *Petrogale penicillata*. The left lateral side of the skull is shown in **a**,**d**, and **g**, the dorsal view in **b**,**e**, and **h**, and the ventral view in **c**,**f**, and **i**. Images were created using MeshLab 1.3.3 and Adobe Illustrator CS6. **a**,**b**, and **c** represent ZMB_EMB_MA587, the earliest of the three stages; **d**,**e**, and **f** represent ZMB_EMB_MA590, the intermediate stage; and **g**,**h**, and **i** represent ZMB_EMB_MA593A, the most advanced of the three stages illustrated here. Plates of the other four species can be found in the Suppl. Figs 3, 4, 5, and 6. Abbreviations (for all figures): as (alisphenoid), bo (basioccipital), bs (basisphenoid), de (dentary), eo (exoccipital), et (ectotympanic), fr (frontal), go (goniale), in (incus), ip (interparietal), ju (jugal), la (lacrimal), ml (malleus), mx (maxilla), na (nasal), os (orbitosphenoid), pa (parietal), pe (petrosal), pl (palatine), pm (premaxilla), pr (presphenoid), pt (pterygoid), so (supraoccipital), sq (squamosal), vo (vomer).

Because of the rare and historic value of the specimens from the Hill–Collection, we have chosen the non-invasive technique of micro-computed tomography (μCT) to analyze all 115 specimens. To prevent shrinkage, all specimens were wrapped in several layers of synthetic sponge that had been wetted in ethanol. The wrapped specimens were subsequently put into tubes that were closed off with either a cap or tape and secured inside the scanner. All studied specimens were scanned at the Museum für Naturkunde Berlin using a Phoenix nanotom X-ray|s tube at between 50 and 90 kV and 150 to 300 μA, depending on the size of the specimen. For each scan 1000 projections were generated with 750 ms per scan. Effective voxel size ranged between 2 and 3.33 μm. The cone beam reconstruction was performed using the datos|x-reconstruction software (GE Sensing & Inspection Technologies GMBH phoenix|x-ray datos|x 2). Isosurface renderings were produced in VG Studio Max 2.2. Because the settings used for scanning varied depending on the size of each specimens, the gray-scale thresholds used for the isosurface renderings also differed between specimens and were set to include as many bone fragments as possible without including any non-calcified tissue, such as cartilage, skin, or muscle. Isosurfaces were processed as .ply or .stl files using MeshLab 1.3.33^28^, in which any noise from the scan was removed by deleting isolated pieces by diameter or manually. Images were further edited in Adobe Photoshop CS6 and Adobe Illustrator CS6 (see Fig. 2). For each species, also one adult skull from the Mammal Collection of the Museum für Naturkunde Berlin was μCT-scanned (Suppl. Table 1).

### Analytical framework 1: onset of ossification

We used two approaches to study cranial bone development in marsupials. The first, conventional approach was to study cranial osteogenesis by determining the onset of ossification of 25 bones (Suppl. Tables 4–10). Because no data on the absolute age of the specimens were available, the specimens were ordered based on the bone onset timing and were subsequently ranked with each new rank showing the first appearance of one or more bones (sensu Koyabu *et al*.^29^, Suppl. Tables 4–8). A relative scaling was made to create a matrix containing continuous data between 0 and 1 (sensu Werneburg *et al*.^30^,^31^ and Germain and Laurin^32^; Suppl. Tables 9 and 10). We only included species for which six or more ranks were available in order to decrease the possibility of errors related to sample size^29^,^30^. By combining the data on the onset of ossification from our study with data from the literature as assembled by Koyabu *et al*.^29^, we compared marsupial cranial osteogenesis in 10 different species; seven species from Koyabu *et al*.^29^ and four species from our study, with *T. vulpecula* being included twice using data from both studies. We excluded the data of *D. viverrinus* from this analysis because only four different ranks of bone onset could be distinguished. The interparietal, malleus, presphenoid, and incus were not studied by Koyabu *et al*.^29^.

Thus, we reported variation in the timing of bone onset among the studied species by presenting the minimal and maximal values for the ossification timing of each bone, as well as by calculating their respective median and 25^th^ and 75^th^ percentiles. Furthermore, we reconstructed the relative ancestral timing of ossification for each bone. We calculated ancestral values using squared-change parsimony analysis^33^, taking divergence time into consideration in the form of the branch lengths in a molecular time scaled phylogeny. We chose this method of ancestral trait reconstruction over other methods such as event-pairing or Parsimov because this continuous analysis has been convincingly shown to be more reliable^32^,^34^. Furthermore, we calculated the 70% confidence intervals (CIs) of the reconstructed timing for each bone to determine the statistical strength of the reconstructions (sensu Werneburg *et al*.^30^).

Our molecular time scaled phylogeny was modified from the one used by Koyabu *et al*.^29^ by adding the species of the present study using Mesquite 3.03^35^. Topology and divergence time of the added taxa were determined using The Timetree of Life^36^. The divergence time of *Ma. eugenii* and *Pe. penicillata* were retrieved from http://www.timetree.org/ (2016-11-22) and were scaled to the other divergence times by calculating the deviation between the divergence time found for Potoroidae and Macropodidae from http://www.timetree.org/ and Springer *et al*.^36^ (scale = 0.5617).

### Analytical framework 2: bone contacts

The second, non-traditional, approach was to study the onset of bone contacts (Suppl. Tables 11–21). This is the first study to quantify initial bone contact (note: cranial suture closure has previously been studied quantitatively, e.g. by Wilson and Sánchez-Villagra, Rager *et al*., and Wilson^37^,^38^,^39^). Bone contacts were defined as clearly observable contacts between the different cranial bones in the .ply and .stl files. We excluded contacts that were only formed by three or less triangles, because these contacts were likely to be the result of the reconstruction process rather than representing an actual connection between two bones (for further discussion, see Suppl. Fig. 7). We ordered the specimens based on bone contact and ranked them (as for the onset of ossification data, Suppl. Tables 11–15). The contacts were scaled and the median and 25^th^ and 75^th^ percentiles were calculated. Ancestral values and 70% CIs were calculated in Mesquite^35^, using the same phylogeny as for the onset of ossification analysis. The last common ancestor of all taxa for which bone contact was analyzed was the last common ancestor of all Australidelphia (i.e., Marsupialia excl. the American forms) (Fig. 1).

## Results

### Onset of ossification

In total, 25 different bones were analyzed for their onset of ossification. In the following, we summarize our results and briefly describe differences to the findings of previously published papers.

**Figure 3:**
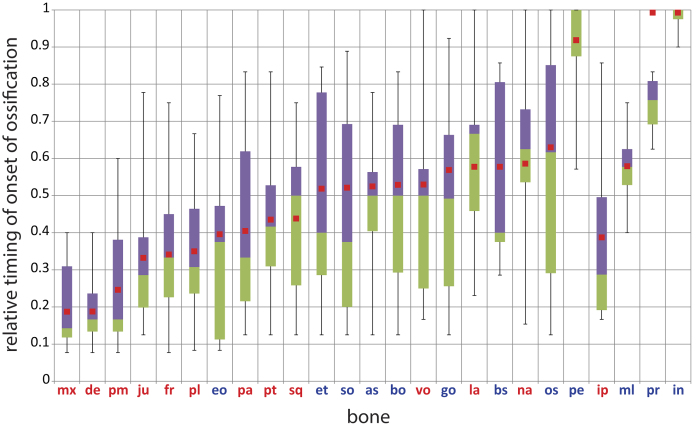
Boxplot comparison showing the range of variation for the relative timing of the onset of ossification. The 25th percentile is marked by the lower edge of the box, the 75^th^ percentile by the upper end. The median is marked by the boundary between the purple and green parts of the box. The upper and lower whiskers mark the maximal and minimal value, respectively. The reconstructed timing of the onset of ossification for each bone in the marsupial ancestor is shown by the red squares. The bones are ordered by the onset of ossification in the ancestor of all marsupials. Bones colored in red are of dermal origin, bones colored in blue of endochondral origin (sensu Koyabu *et al*.^29^ and Hall^55^). The variation in onset of ossification is very large for all bones and the reconstructed ossification sequence follows previous authors in that the bones surrounding the mouth cavity are among the first bones to ossify^22^,^23^,^29^. For a graphic representation of the onset timing for each individual species see Suppl. Fig. 1. The values for this graph can be found in Suppl. Table 2.

The relative timing of all bones in species with more than six ranks are summarized in Fig. 3. The general sequence of ossification found in this study confirms previous studies on marsupials in that the bones of the oral region are the first to ossify (maxilla, premaxilla, and dentary)^22^,^23^,^29^.

We only included pouch young (=postnatal) specimens in our study because we did not detect ossification centers in any of the fetal specimens of the collection. This supports the observation of Gemell *et al*.^23^ that cranial ossification commences distinctly after birth in *Isoodon macrourus* and *Trichosurus vulpecula*. However, Clark and Smith^22^ reported the presence of an already ossified premaxilla, maxilla, dentary, palatine, and pterygoid at birth in *Monodelphis domestica* and *Macropus eugenii*. In the case of *Ma. eugenii*, the presence of these bones at birth does not necessarily contradict the observations of the present study because this species is more precocial than any of the species studied here^11^. For *Mo. domestica*, on the other hand, this observation seems more contradictory, since this species only shows an intermediate stage of development at birth among marsupials^3^,^11^.

**Figure 4:**
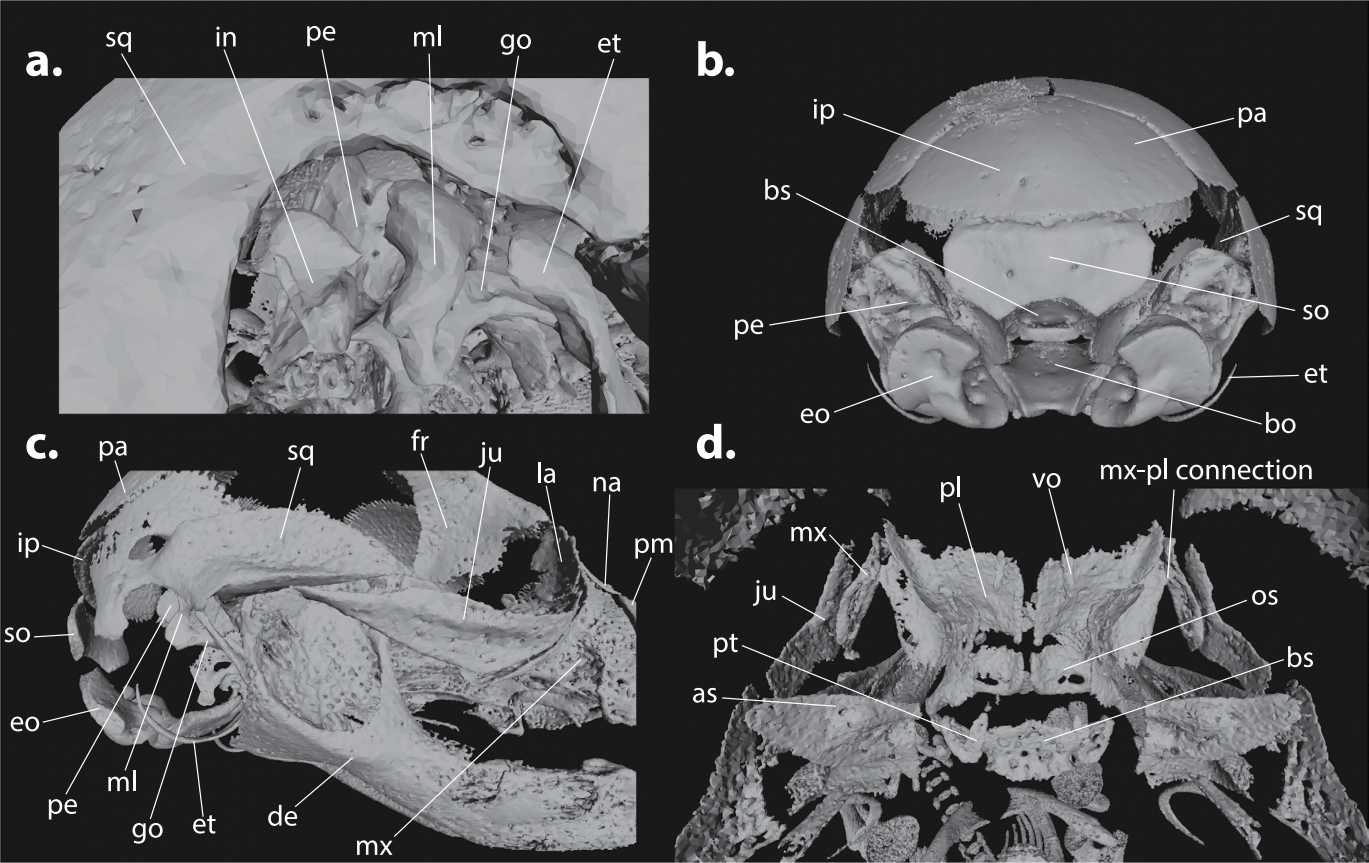
(**a**) Right lateral view of the ear region of *Trichosurus vulpecula* ZMB_EMB_MA400 showing the incus, malleus, goniale, and dorsal part of the ectotympanic. In most species the goniale detaches from its surrounding bones at the final stages of cranial bone development (Suppl. Tables 11–15). The connection of the goniale to the ectotympanic is the result of their close proximity across most of their length (see also Fig. 5B). Similarly, this is likely also the case for the connection of the malleus and incus. (**b**) Posterior view of the skull of *T. vulpecula* ZMB_EMB_MA400 showing a large interparietal. In *T. vulpecula*, the interparietal has a very early onset and large size early in development, being much larger than the supraoccipital. It appears to generally have one center of ossification, although intraspecific variation occurs. It remains very prominent in the adult skull, even connecting to the squamosal bone, and forms the posterior part of the cranial dome. In *Dasyurus viverrinus*, the interparietal can be clearly distinguished in the earliest developmental rank (ZMB_EMB_MA759) but cannot be found in more developed specimens, indicating a very early fusion of the interparietal with the supraoccipital. In *Phascolarctos cinereus*, the interparietal generally has three ossification centers; otherwise its development is similar to *T. vulpecula*, although it does not make contact with the squamosal. In contrast to the other species, the interparietal ossifies relatively very late in *Vombatus ursinus*. It is comparatively very small throughout cranial development, shows two centers of ossification, and most likely fuses completely with the parietals. The interparietal appears to have one ossification center in *Petrogale penicillata* (0.38), but otherwise seems to follow the same general developmental pattern as in *Ph. cinereus and T. vulpecula*. (**c**) Lateral view of the skull of *Pe. penicillata* ZMB_EMB_MA564 showing the jugal, maxilla and squamosal bones that form the jugal arch. (**d**) Dorsal view of the sphenoid region of *Ph. cinereus* ZMB_EMB_MA495B showing the clear distinction of the individual bones, as well as the presence of early ossification centers of both vomers. Furthermore, the early connection between the maxilla and palatine can be observed.

**Figure 5:**
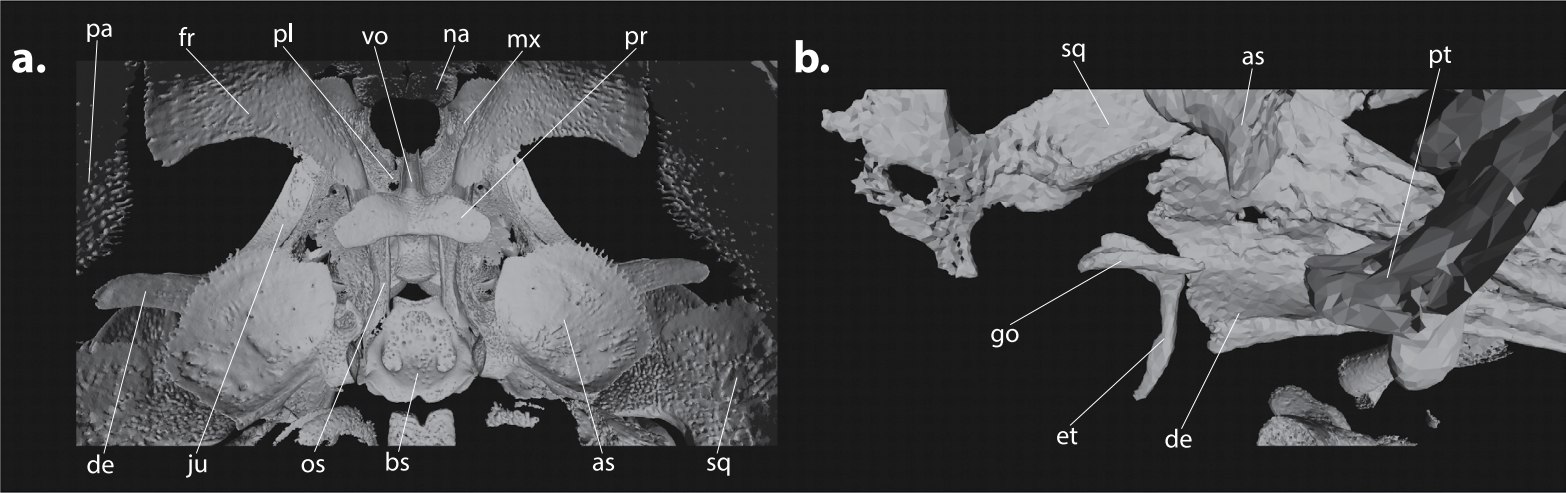
(**a**) Posterodorsal view of the sphenoid region of *Petrogale penicillata* ZMB_EMB_MA585 showing the shape and location of the presphenoid. This bone is often overlooked in osteological studies; it originates from two different ossification centers at the anterior part of the braincase. Its ventral part connects successively to the orbitosphenoid, vomer, and basisphenoid, whereas the dorsal part forms two winglike lateral extensions that first connect to the frontal and at a later stage to the alisphenoid. Regarding the presphenoid, Clark and Smith^22^ reported that it connects to the orbitosphenoid almost immediately after the individual ossification of both bones. Although the orbitosphenoid is generally the first bone the presphenoid connects to in our data, it occurs late in development (median: 0.79), and the orbitosphenoid first connects to the maxilla (0.14), alisphenoid (0.69) pterygoid (0.69), and frontal (0.73) before connecting to the presphenoid. (**b**) Medial view of the middle ear region of *Pe. penicillata* ZMB_EMB_MA587 showing the close proximity of the goniale and ectotympanic.

Our μCT-data permitted the identification of bones that were not treated by Koyabu *et al*.^29^, namely two middle ear bones (malleus and incus), the interparietal, and the presphenoid (Figs 4a,b and 5). However, these bones have previously been studied, among other cranial bones, in *Mo. domestica* and *Ma. eugenii* by Clark and Smith^22^.

According to Clark and Smith^22^, the incus is the last bone to ossify, with the exception of the stapes, the latter of which has not been treated in our study because it could not be distinguished in the μCT-scans. This concurs with our results, in which the incus is the last bone to ossify (median: 1.00; potentially excluding the stapes, which was not analyzed herein). Furthermore, Clark and Smith^22^ reported that the malleus ossifies 11 and 21 days after birth in *Mo. domestica* and *Ma. eugenii*, respectively, which is relatively late. This could also be confirmed for the relative timing of the malleus in our study (median: 0.58; Fig. 3).

The interparietal is present as a postparietal bone (Fig. 4b) and ossifies eight days after birth in *Ma. eugenii* and and three days after birth in *Mo. domestica*^22^, which is relatively earlier than in *Vombatus ursinus*, but later than in *T. vulpecula, Dasyurus viverrinus, Phascolarctos cinereus*, and *Petrogale penicillata* (Fig. 3).

The onset of ossification data show a large amount of variation. The variation in relative onset of ossification is more than 75% in six of the 25 bones, namely in the ossification of the supraoccipital (min-max: 0.13–0.89; Fig. 3), vomer (0.17–1.00), goniale (0.13–0.92), lacrimal (0.23–1.00), nasal (0.15–1.00), and orbitosphenoid (0.13–1.00). The incus, which was only studied in four of the 10 species, is the only bone that shows less than 25% variation (0.90–1.00) (Fig. 3).

The 70% confidence intervals for the reconstructed ancestral sequence of ossification are very broad. Only the squamosal (70% lower CI: 0.30 – reconstructed value: 0.4–70% upper CI: 0.57), vomer (0.35–0.5–0.71), goniale (0.40–0.6–0.70), and petrosal (0.83–0.92–0.95) are relatively narrow (Suppl. Table 2). Overall, this indicates a very high uncertainty for the reconstructed onset of ossification values. As such, any comparison with the reconstructions of the ancestral ossification sequence of Mammalia by Koyabu *et al*.^29^ has to be handled with care. However, compared to the mammalian ancestor reconstructed by Koyabu *et al*.^29^, the premaxilla, squamosal, pterygoid, and nasal ossified distinctly earlier in the marsupial ancestor as reconstructed herein. The exoccipital ossified distinctly later in the marsupial ancestor.

### Bone contacts

**Figure 6:**
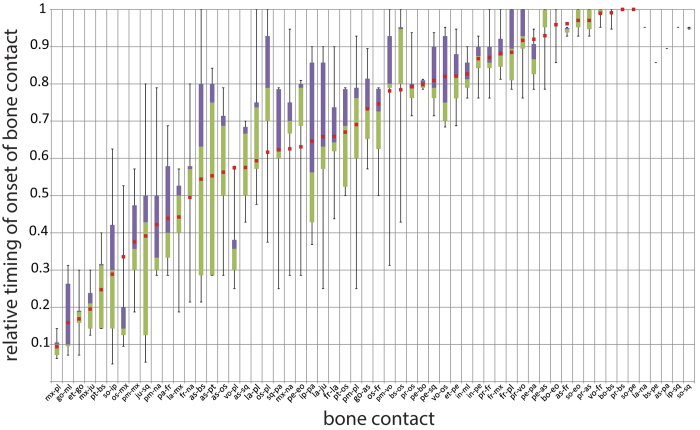
Boxplot comparison showing the range of variation for the relative timing of the bone contacts. The reconstructed timing of bone-to-bone contact of the last common ancestor of all studied species (Australidelphia) is marked by the red squares. The bone contacts are ordered based on the bone contact sequence in the last common ancestor. The bone contacts clearly show less variation in timing than bone onset (see Fig. 3). The contacts of the occipital and middle ear regions, as well as the contacts of the petrosal and the maxilla-palatine contact show especially little variation. The values for this graph can be found in Suppl. Table 3.

In total, 64 different bone contacts were defined, out of which 50 contacts were observed in all species and 14 contacts could only be observed in certain species (see Suppl. Tables 17–19). A boxplot of the relative timing of the bone contacts and the reconstructed bone contact sequence for the ancestor of the marsupials is shown in Fig. 6. In comparison to the reconstructed ancestral values for the onset of ossification, the reconstructed bone contact values show very narrow confidence intervals (Suppl. Table 3).

Most bones that connect at an early stage are bones surrounding the mouth cavity [maxilla-palatine (median: 0.10; Fig. 6), maxilla-jugal (0.21), orbitosphenoid-maxilla (0.14), premaxilla-maxilla (0.36), and premaxilla-nasal (0.33) and the goniale and the bones to which it connects: goniale-malleus (0.10) and ectotympanic-goniale (0.19)].

The bones that are among the last to make contact are the bones that make up the back of the skull, particularly the bones of the occipital region [supraoccipital-petrosal (median: 1.00), basioccipital-basisphenoid (1.00), supraoccipital-exoccipital (1.00), basioccipital-exoccipital (1.00), petrosal-alisphenoid (1.00), and petrosal-parietal (0.87)]. The very late connection between the vomer and the frontal is created by the formation of the nasal septum (1.00).

The bone contact data also show a high degree of variation (Fig. 6). However, whereas for the onset of ossification variation is high in all bones, this is not the case for bone contact, where the bones that connect very early or late show a much lower range of variation than the intermediate contacts.

Contacts such as the jugal-squamosal (min-max: 0.05–0.80; Fig. 6), interparietal-parietal (0.37–0.90), supraoccipital-interparietal (0.05–0.63), alisphenoid-basisphenoid (0.21–1.00), and maxilla-nasal (0.29–0.95) show a particularly high range of variation. The high variation in the connection of the interparietal with the parietal and the supraoccipital is obviously effected by the high interspecific variation in the size and shape of the interparietal^40^.

Most bones that connect in the sphenoid region show a high range of variation as well (Fig. 6). The alisphenoid-basisphenoid (min-max: 0.21–1.00) connection shows most variation, but the alisphenoid-pterygoid (0.29–0.84), alisphenoid-orbitosphenoid (0.29–0.79), and basisphenoid-orbitosphenoid (0.43–1.00) connections are also highly variable. The pterygoid-basisphenoid (0.14–0.40) and pterygoid-orbitosphenoid (0.50–0.79) show a much lower range of variation.

The earliest and one of the most conserved connections is the maxilla-palatine connection (median: 0.10; min-max: 0.06–0.14). The petrosal connects to many different bones and these contacts are quite conserved [petrosal-basioccipital (min-max: 0.79–0.81; Fig. 6), petrosal-squamosal (0.71–0.94), ectotympanic-petrosal (0.69–0.95), incus-petrosal (0.76–0.93), petrosal-parietal (0.79–0.95), petrosal-alisphenoid (0.79–1.00), and supraoccipital-petrosal (1.00–1.00)] and are established very late in development.

The connections of the occipital region also occur very late and show a small range of variation [basioccipital-exoccipital (min-max: 0.86–1.00), supraoccipital-exoccipital (0.93–1.00), and basioccipital-basisphenoid (0.95–1.00)] (Fig. 4b and 6). In the ear region, the connections of goniale and malleus (min-max: 0.07–0.31), goniale and ectotympanic (0.07–0.30), and malleus and incus (0.76–0.90) are very conserved (Fig. 4a).

The presphenoid (Fig. 5a) ossifies relatively late (onset of ossification median: 0.76) and connects successively to the orbitosphenoid (bone contact median: 0.79), frontal (0.86), vomer (0.93), alisphenoid (1.00), and basisphenoid (1.00) (Figs 3 and 6). These connections also occur generally late in development and are quite conserved [presphenoid-orbitosphenoid (min-max: 0.71–0.94), presphenoid-frontal (0.76–1.00), presphenoid-vomer (0.76–1.00), and presphenoid-alisphenoid (0.93–1.00)], especially the presphenoid-basisphenoid connection (1.00–1.00), which only develops in the last rank for all species.

## Discussion

Our study revealed a large variation in the onset of ossification of all cranial bones (Fig. 3). In contrast, bone to bone contacts are constrained in many cases (Fig. 6). The calculated confidence intervals for the reconstructed ancestral values are much broader for the onset of ossification than for bone contact, showing that onset of ossification reconstructions are comparatively much less reliable (Suppl. Tables 2 and 3). This challenges many traditional studies that use the onset of ossification as their marker for phylogeny or as a functional proxy. Because onset of ossification per definition precedes bone contact, one could argue that there is an inherent correlation between onset and first contact timing and that it seems contradictory that, within the process of bone development, a highly variable phase is followed by a much more conserved phase. In part, the variability of contact timing is diminished simply because we could observe a greater number of bone contacts: 64 bone contacts compared to 25 bones observed. This larger amount of contacts logically leads to a larger number of different ranks from which the relative scaling is established (Suppl. Tables 9 and 10 for the onset of ossification and Suppl. Tables 16–21 for bone contact), thereby diminishing the randomness. Mathematically speaking, bone number is inverse to contact variability. As such, the absolute randomness of both datasets are not comparable, as is supported by the narrower confidence intervals calculated for bone contact compared to onset of ossification.

Nonetheless, we had expected to find conservation in the onset of ossification in marsupials as they are strongly affected by constraints in early postnatal development related to early delivery and the tight fixation to the mother’s teat. However, the high variation observed likely indicates that bones offer only very little functional support to the head during early postnatal development in marsupials. Actually, cranial osteogenesis has yet to start at birth in all of the studied species and instead the cartilaginous chondocranium is strongly developed within a very short time span in the neonate to withstand ex utero conditions in general and the forces associated with suckling activities in particular^41^.

The early ossification of the premaxilla, maxilla, dentary, palatine, and pterygoid in marsupials has been suggested by Clark and Smith^22^ to represent an adaptation to suckling and the attachment to the teat. However, these bones are the first to ossify in the crania of monotreme and placental mammals (and sauropsids) as well^29^,^42^. Monotremes do not suckle but instead lick the milk from a field enclosed by skin folds in which the milk is collected^43^. Therefore, instead of being a specialized adaptation to suckling, early ossification of the bones surrounding the mouth cavity seems to be the ancestral condition in mammals, as was reconstructed by Koyabu *et al*.^29^.

One traditional approach to deal with the timing of ossification is to speculate a correlation of developmental timing and adult prominence of an element. The related “rule of thumb” states that the earlier a structure occurs in development, the larger or the more differentiated the structure appears in the adult stage and the later it occurs, the smaller or the less differentiated the structure appears^44^,^45^,^46^,^47^,^48^,^49^,^50^. This is because earlier appearing structures have more time to develop than later appearing structures. This “rule of thumb” has been tested by Werneburg *et al*.^51^, revealing that only in very few cases a clear correlation exists between ontogenetic timing and adult appearance of a structure. There are many possible reasons for this, including different speeds of development between different elements. Also, single elements can have faster and slower phases of development. Moreover, a comparative quantification of adult bone prominence and complexity is very difficult, which makes a correlation to the relative timing of the first appearance of the structures very complicated. The whole issue becomes even more challenging when considering the observed high variation in the onset of ossification among the very similar species of our study. The petrosal bone, for example, ossifies very late and also makes contact with other bones late in development (Figs 3 and 6). The relative timing in the onset of ossification is comparatively conserved and the petrosal increases in size rapidly to connect to a number of other bones. This contradicts the “early equals important rule” that bones that ossify earlier are larger and more differentiated in the adult stage. Simultaneously, the very late onset of ossification and subsequent rapid growth after onset explains the conserved connections of the petrosal with other bones.

Given the results of our study, a well supported discussion can only be drawn on ontogenetic structures of little variation among species. This will consequently result in the discussion of highly conserved developmental processes such as the observed earlier ossification of the malleus in comparison to the incus. This pattern probably recapitulates the development of the articular (malleus) bone in synapsid ancestors, which, as a part of the mandible, would have ossified earlier than the quadrate (=incus)^52^ (Fig. 3).

We observed high variation for most bone contacts. However, certain contacts were conserved (Fig. 6). These were generally established either quite early or late in development. The bones of the oral region, as well as the goniale and its surrounding bones, connect early and in doing so, they correspond to the (generally) early ossification of these bones.

In many cases, the observed variation in bone contacts can primarily be explained through characteristics of individual bones during ontogeny that do not necessarily constitute any detectable evolutionary value, such as shape and proximity to other bones. In other cases, the absence or presence of variation in the timing of bone contacts seems to indicate the functional requirements of these connections during ontogeny. Therefore, the only way to infer the meaning of the observed timing of bone contacts is to interpret every contact based on the individual development of each bone and its relationships with the other bones surrounding it.

The ectotympanic, for example, is located near the posterior end of the dentary in mammals and is homologous to the angular in non-mammalian amniotes^53^. In all studied species, it shows an early and conserved connection to the goniale, which is homologous to the prearticular, with which it forms a ring-like structure from which the tympanic membrane or eardrum is spanned (Figs 4c and 5b)^52^. The incus-malleus connection, which facilitates the advanced hearing in mammals and is therefore also functionally important, is also quite conserved (Fig. 6).

The maxilla-palatine contact, the contacts of the occipital region, the petrosal contacts, and the middle ear bone contacts are particularly constrained and show little variation (Fig. 6). The earliest and one of the most conserved connections is the maxilla-palatine connection; this might be an important adaptation for suckling as it supports the palatine region (Fig. 4d). Similarly, the late timing of a conserved bone-to-bone connection could also indicate the functional role of a structure. Namely, the late and constrained connections of the occipital region could indicate that this area does not have an important function for cervical support during suckling early in development. Therefore the cervical support provided by the swelling of non-ossified tissue is likely sufficient^11^.

Most bones that connect in the sphenoid region show a high range of variation (Fig. 6). This overall high variability is most likely related to the proximity of these connections to each other (Fig. 4d, but see also Suppl. Fig. 7B). Because of their close proximity, a small variation in the position of the bones can result in relatively large differences in the onset of bone contact.

It is important to note that the jugal-squamosal contact is highly variable. Its connection implies the formation of the jugal arch, which supports part of the jaw musculature (mm. masseter et zygomandibularis) and the formation of the eye socket^54^. Therefore, the jugal arch is an important structure for both feeding and perception (Fig. 4c). The high variation observed in this connection, however, seems to indicate that the jugal arch is not of strong functional importance during marsupial development in the pouch. The early and constrained maxilla-jugal connection, however, which connects the anterior part of the jugal arch, contrasts with this very variable jugal-squamosal contact (Fig. 4c and 6).

The high variation in the maxilla-nasal connection could be explained in that this connection is generally very small, as the nasal mainly connects to the premaxilla to form a connection with the lower part of the skull. Therefore, the maxilla-nasal connection most likely does not constitute an important supporting function and is dependent on the size and formation of the snout (see Suppl. Fig. 7D).

The late connections of certain bones to the frontal (frontal-maxilla, frontal-palatine, alisphenoid-frontal, and formation of the nasal septum: vomer-frontal) (Fig. 6) represent the connection of the upper and lower parts of the cranium, which are further formed during early development by the premaxilla and nasal and in the middle of development by the squamosal and parietal, anteriorly and posteriorly, respectively.

The bones in the oral region have both an early onset of ossification and are among the first bones to connect (Figs 3 and 6). Overall, this might indicate that in the altricial marsupial neonate suckling forces require more structural support from the cranial bones of the oral region than the cervical region requires for the connection between the body and the head. The support for this connection is already largely provided by the cervical swelling in the early neonate^11^,^17^.

Whereas the early onset of ossification is very variable and not unique to marsupials (see above), the bone contact formations are more conservative. The latter result in similar interpretations as provided for ossification in the past^11^,^22^,^23^,^29^ but having greater statistic support. However, in order to further substantiate such a statement, the timing of bone contacts in the other two major mammalian clades has to be investigated in the future.

## Conclusion

The results of this study indicate that studying bone contact is a very suitable marker for studying cranial osteogenesis, since it offers more data than the onset of ossification and is less variable partly because of this. By observing and interpreting the timing of every bone contact individually, particularly for contacts that show little variation, its timing and degree of variation can give valuable insights into phylogeny and functional requirements early in development (e.g., the early connection of the middle ear bones and the maxilla-palatine connection). Furthermore, bone contacts give insight into much later stages of development than can be observed through studying onset of ossification alone. Therefore, bone contact should be considered an important proxy in studies of cranial osteogenesis in vertebrates.

## Additional Information

**How to cite this article**: Spiekman, S. N. F. and Werneburg, I. Patterns in the bony skull development of marsupials: high variation in onset of ossification and conserved regions of bone contact. *Sci. Rep.* **7**, 43197; doi: 10.1038/srep43197 (2017).

**Publisher's note:** Springer Nature remains neutral with regard to jurisdictional claims in published maps and institutional affiliations.

## Supplementary Material

Supplementary Information

Supplementary Dataset 1
