# Supplementary material for: Patterns in the bony skull development of marsupials: high variation in onset of ossification and conserved regions of bone contact
Source: Sci Rep. 2017 Feb 24;7:43197. doi: 10.1038/srep43197 (PMC5324120; doi:10.1038/srep43197)
Supplement: Supplementary Information [file srep43197-s1.doc]

**Patterns in the bony skull development of marsupials: high variation in onset of ossification and conserved regions of bone contact**

Stephan N. F. Spiekman, Ingmar Werneburg

**Supplementary Information**

# SUPPLEMENTARY FIGURES


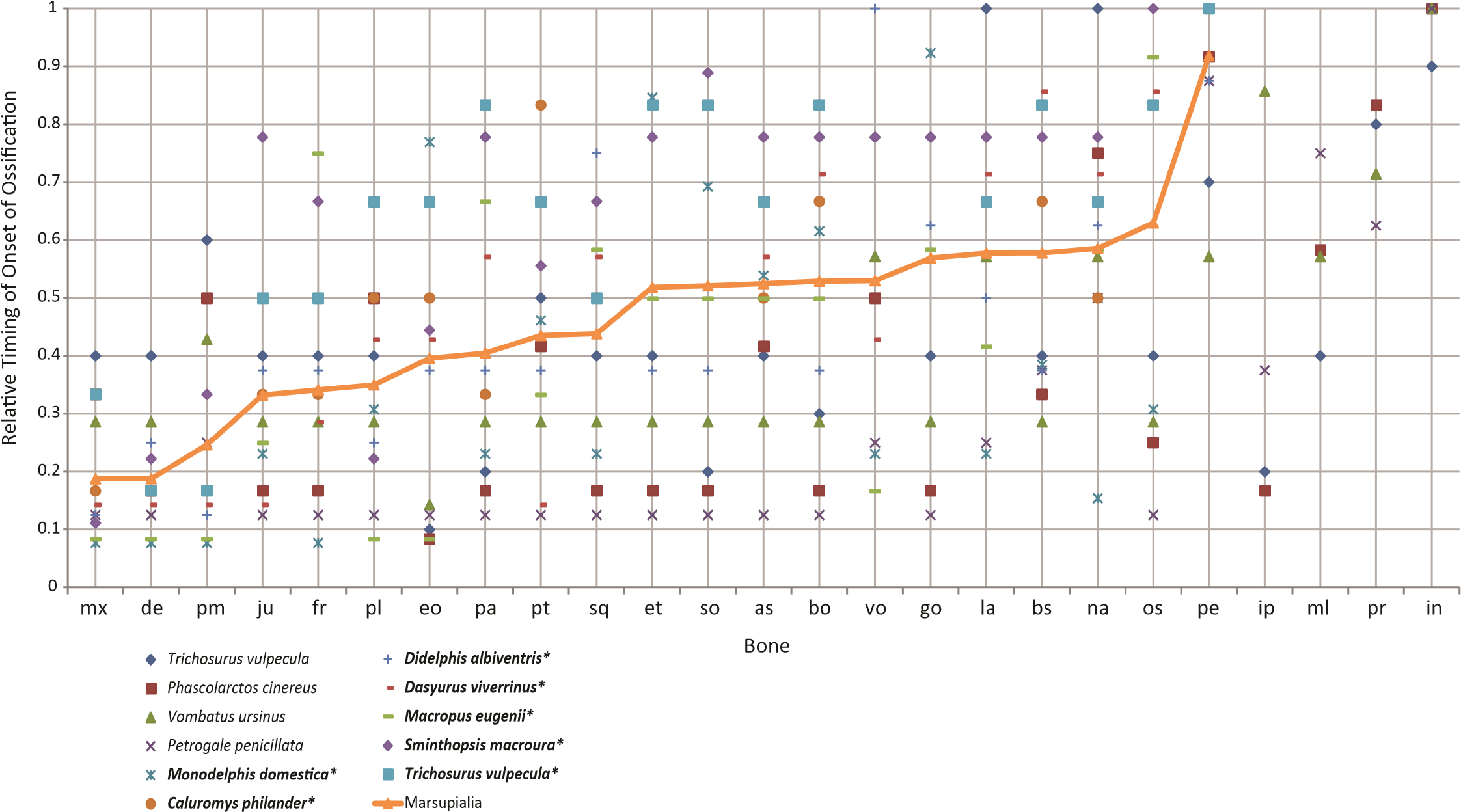


**Supplementary Figure 1.** The reconstructed timing of the onset of ossification in the marsupial ancestor (orange line) compared to the onset of ossification of the species studied in this study and by Koyabu et al. 2014 (marked in bold and with an asterisk) containing at least six ranks. The bones are ordered by the onset of ossification in the ancestor of all marsupials. The variation is high and only few consistencies can be found within the phylogenetic groups. In the carnivorous Dasyuridae, *Dasyurus viverrinus* and *Sminthopsis macroura*, the maxilla develops earlier than in the reconstructed marsupial ancestor, whereas the parietal, basisphenoid, squamosal, nasal, alisphenoid, basioccipital, and lacrimal all ossify distinctly later in the carnivores than in the reconstructed marsupial ancestor. Most bones in *S. macroura* ossify very late compared to all other species including *D. viverrinus*. In Didelphidae, the maxilla and premaxilla ossify early compared to other species and the marsupial ancestor. In the Vombatiformes the majority of the bones ossify distinctly earlier than in the other species and the reconstructed marsupial ancestor. The maxilla and premaxilla, however, ossify later than in the reconstructed marsupial ancestor. In Macropodidae, the maxilla, dentary, palatine, exoccipital, and vomer ossify comparatively early. The two datasets of *Trichosurus vulpecula* differ in their values for many bones. Compared to the reconstructed marsupial ancestor, the maxilla, jugal, frontal, palatine, and pterygoid develop late in both datasets of *T. vulpecula*.


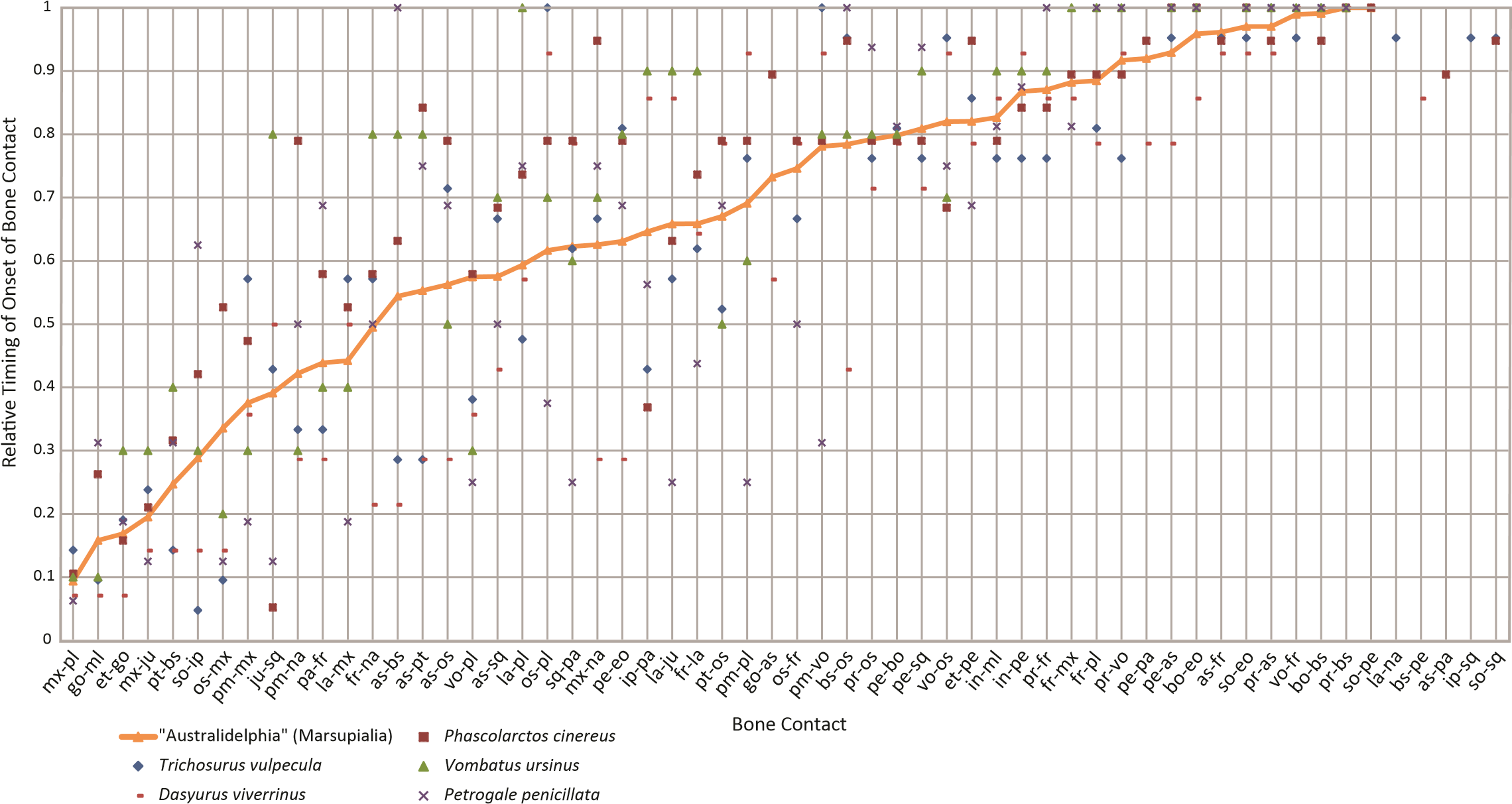


**Supplementary Figure 2.** The reconstructed timing of bone-to-bone contact of the last common ancestor of all studied species (Australidelphia, orange line) is compared to the timing of bone contact of species of this study. The bone contacts are ordered based on the sequence in the last common ancestor. Whereas data on the onset of ossification was available for ten different marsupial species, bone contact has not been studied previously and therefore data is only available for the five species that we describe here. In contrast to the observed onset of ossification, the timing of individual bone contacts reflects functional requirements and phylogenetic history in some of the contacts, particularly the contacts that show the least amount of variation.

**Supplementary Figure 3.** Three different stages of cranial bone development in *Trichosurus vulpecula*. The earliest development stage shown is ZMB_EMB_MA454 (A, B, and C), the intermediate stage is ZMB_EMB_MA358, and the most developed stage is ZMB_EMB_MA397. Images show the left lateral skull view in A, D, and G; the dorsal view in B, E, and H; and the ventral view in C, F, and I. Images were created using MeshLab 1.3.3 and Adobe Illustrator CS6. The high range of variation in the onset of ossification is highlighted by the comparison of the two different datasets for *T. vulpecula*, of which one set was collected during our study and the other set was retrieved from Koyabu et al. 2014, which combined original data with data from Gemmell et al. 1988 for this species. These two datasets generally do not show more similarities with each other than they do with the datasets of any other studied species. However, it is relevant to note out a few differences between the sample collected during this study and the data from Koyabu et al. 2014. A number of bones ossify distinctly earlier in the species studied in this study and one bone, the premaxilla, generally ossifies later in our data compared to the data of Koyabu et al. 2014. It is important to note that for our species four more bones were studied, which were all relatively late in their development. Therefore, all other bones appear earlier on a relative time scale. Nonetheless, this pattern might also be the result of other factors, such as preservation and method of data acquisition, which differed between the two studies. For example, although most species were studied using µCT-scanning by Koyabu et al. 2014, data for *Didelphis albiventris*, *Dasyurus viverrinus*, and *T. vulpecula* were obtained using clearing and double staining. However, when comparing the data for these three species to the other studied species, no similarities can be found. Therefore, this different method of data acquisition does not seem to explain the differences between the two sets of data observed in *T. vulpecula*.

**Supplementary Figure 4.** Three different stages of cranial bone development in *Dasyurus viverrinus*. A, B and C represent ZMB_EMB_MA759, D, E and G ZMB_EMB_MA750 and G, H and I ZMB_EMB_752A. For orientation compare with Supplementary Figure 3.

**Supplementary Figure 5.** Three stages of cranial bone development in *Phascolarctos cinereus*. A, B and C show ZMB_EMB_MA485A, D, E, and F ZMB_EMB_MA483 and G, H, and I ZMB_EMB_MA492. For orientation compare with Supplementary Figure 3. Note the presence of a distinct bulbing of the maxilla in *Ph. cinereus* ZMB_EMB_MA492 (G and H). This bulbing can be observed in four specimens, constituting ranks 15 to 18 in the bone contact matrix (Supplementary Table 13), the last developmental stages before the adult stage. A similar shape for the maxilla cannot be observed for any of the other studied species and it does not seem to constitute any functional adaptation. This bulbing cannot be observed in the adult skull and has not been described previously.

**Supplementary Figure 6.** Three different stages of cranial bone development in *Vombatus ursinus*. A, B, and C represent ZMB_EMB_MA524, D, E, and F represent ZMB_EMB_MA538 and G, H, and I represent ZMB_EMB_MA523. For orientation compare with Supplementary Figure 3.


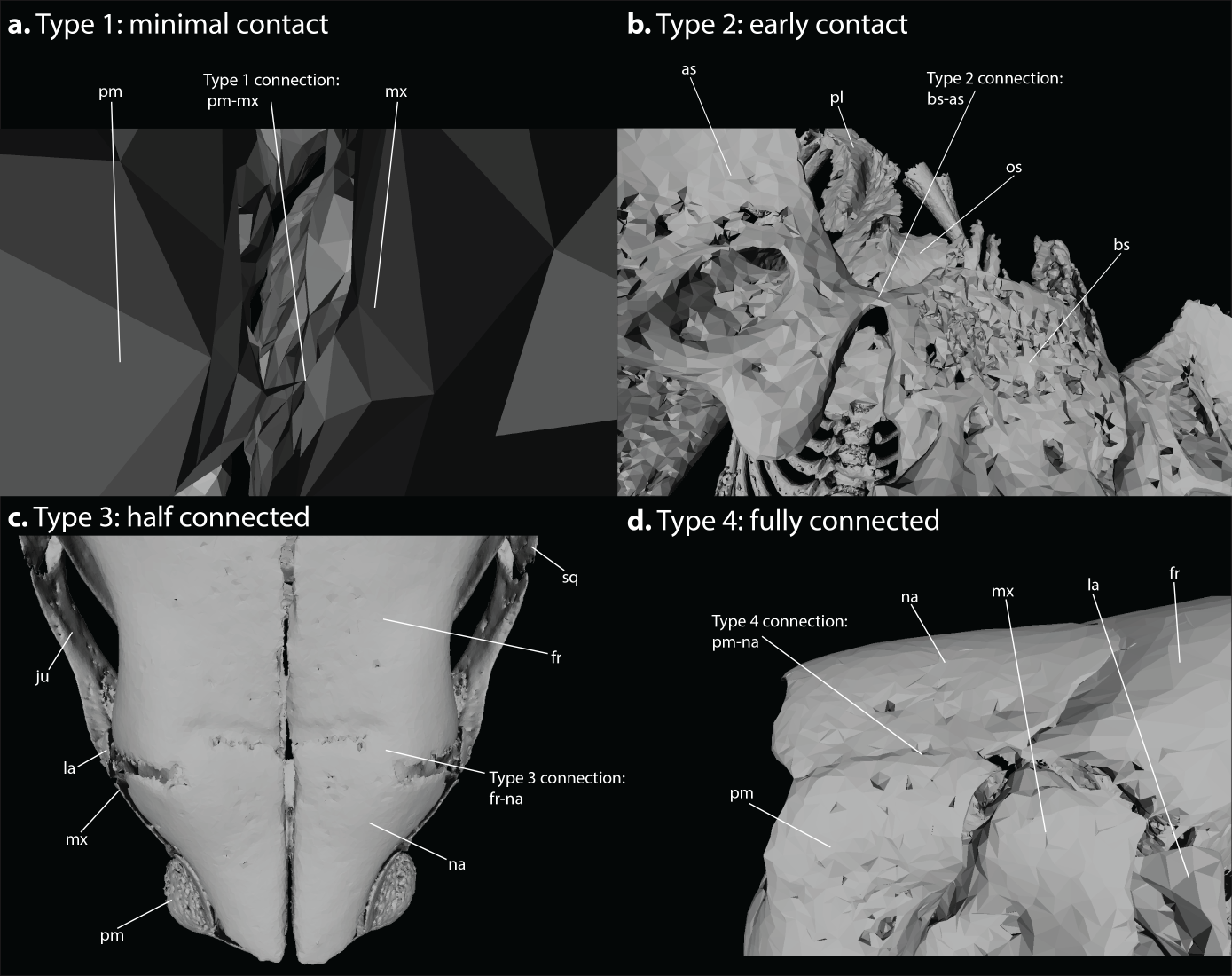


**Supplementary Figure 7.** Four different types of contact were distinguished to cover the different degrees of bone contact and were coded for in Supplementary Tables 11-15. However, in the main text, only the initial contact is considered for our analyses and discussion. Here, examples of each of the four types of bone contact that were used to distinguish bone connections are shown. **A)** Contact between two bones consists of less than three triangles in the .ply or .stl file; exemplified by the connection between the premaxilla and maxilla in *Phascolarctos cinereus* ZMB_EMB_MA495B (dorsal view). This connection was excluded from the final analyses, because it is likely that such a connection is the result of digital reconstruction, rather than forming an actual biological contact. This was evidenced by a small number of type 1-contacts that were found between bones that do not connect during ontogeny such as contacts between the dentary and the ectotympanic, jugal, alisphenoid, and squamosal. **B**) There is a connection between bones but it is clearly less than half of the final connection between the two bones found in the adult skull; exemplified by the connection between the basisphenoid and alisphenoid in *Trichosurus vulpecula* ZMB_EMB_MA453B (tilted posterodorsal view). **C)** The two bones are connected to about half the extent of the final connection. Exemplified by the connection between the frontal and nasal in *T. vulpecula* ZMB_EMB_MA400 (dorsal view). **D)** The two bones are connected across the length of their final connection as found in the adult skull; exemplified by the connection between the premaxilla and nasal in *Ph. cinereus* ZMB_EMB_MA491.
