# Supplementary material for: Patterns in the bony skull development of marsupials: high variation in onset of ossification and conserved regions of bone contact
Source: Sci Rep. 2017 Feb 24;7:43197. doi: 10.1038/srep43197 (PMC5324120; doi:10.1038/srep43197)
Supplement: Supplementary Dataset 1 [file srep43197-s2.doc]

**Patterns in the bony skull development of marsupials: high variation in onset of ossification and conserved regions of bone contact**

Stephan N. F. Spiekman, Ingmar Werneburg

**Supplementary Dataset**

**Supplementary Table 1.** All the specimens µCT-scanned for this study.

| **Genus** | **Species** | **Collection** | **Specimen ID** | **Specimen ID IW** | **SVL** |
| --- | --- | --- | --- | --- | --- |
| *Trichosurus* | *vulpecula* | ZMB_EMB_ | MA374 | IW1500 | 29 |
| *Trichosurus* | *vulpecula* | ZMB_EMB_ | MA454 | IW1501 | 79 |
| *Trichosurus* | *vulpecula* | ZMB_EMB_ | MA362 | IW1502 | 32 |
| *Trichosurus* | *vulpecula* | ZMB_EMB_ | MA353 | IW1503 | 36 |
| *Trichosurus* | *vulpecula* | ZMB_EMB_ | MA381 | IW1504 | 70 |
| *Trichosurus* | *vulpecula* | ZMB_EMB_ | MA358 | IW1505 | 45 |
| *Trichosurus* | *vulpecula* | ZMB_EMB_ | MA396b | IW1506 | 49 |
| *Trichosurus* | *vulpecula* | ZMB_EMB_ | MA350a | IW1507 | 48 |
| *Trichosurus* | *vulpecula* | ZMB_EMB_ | MA377 | IW1508 | 57 |
| *Trichosurus* | *vulpecula* | ZMB_EMB_ | MA376 | IW1509 | 43 |
| *Trichosurus* | *vulpecula* | ZMB_EMB_ | MA378 | IW1510 | 56 |
| *Trichosurus* | *vulpecula* | ZMB_EMB_ | MA398 | IW1511 | 61 |
| *Trichosurus* | *vulpecula* | ZMB_EMB_ | MA453b | IW1512 | 87 |
| *Trichosurus* | *vulpecula* | ZMB_EMB_ | MA450 | IW1513 | 71 |
| *Trichosurus* | *vulpecula* | ZMB_EMB_ | MA452,1 | IW1514 | 98 |
| *Trichosurus* | *vulpecula* | ZMB_EMB_ | MA379 | IW1515 | 73 |
| *Trichosurus* | *vulpecula* | ZMB_EMB_ | MA350b | IW1516 | 70 |
| *Trichosurus* | *vulpecula* | ZMB_EMB_ | MA402 | IW1517 | 79 |
| *Trichosurus* | *vulpecula* | ZMB_EMB_ | MA447 | IW1518 | 84 |
| *Trichosurus* | *vulpecula* | ZMB_EMB_ | MA401 | IW1519 | 92 |
| *Trichosurus* | *vulpecula* | ZMB_EMB_ | MA449 | IW1520 | 102 |
| *Trichosurus* | *vulpecula* | ZMB_EMB_ | MA452,2 | IW1521 | 107 |
| *Trichosurus* | *vulpecula* | ZMB_EMB_ | MA400 | IW1522 | 108 |
| *Trichosurus* | *vulpecula* | ZMB_EMB_ | MA453a | IW1523 | 88 |
| *Trichosurus* | *vulpecula* | ZMB_EMB_ | MA397 | IW1524 | 88 |
| *Trichosurus* | *vulpecula* | ZMB_EMB_ | MA399 | IW1525 | 129 |
| *Trichosurus* | *vulpecula* | ZMB_EMB_ | MA444 | IW1526 | 129 |
| *Trichosurus* | *vulpecula* | ZMB_EMB_ | MA443 | IW1527 | 147 |
| *Trichosurus* | *vulpecula* | ZMB_EMB_ | MA467 | IW1528 | 264 |
| *Trichosurus* | *vulpecula* | ZMB_Mam_ | 13271 | IW1529 | - |
| *Dasyurus* | *viverrinus* | ZMB_EMB_ | MA759A | IW1530 | 45 |
| *Dasyurus* | *viverrinus* | ZMB_EMB_ | MA750 | IW1531 | 51 |
| *Dasyurus* | *viverrinus* | ZMB_EMB_ | MA752B | IW1532 | 65 |
| *Dasyurus* | *viverrinus* | ZMB_EMB_ | MA735 | IW1533 | 55 |
| *Dasyurus* | *viverrinus* | ZMB_EMB_ | MA758A | IW1534 | 57 |
| *Dasyurus* | *viverrinus* | ZMB_EMB_ | MA718 | IW1535 | 72 |
| *Dasyurus* | *viverrinus* | ZMB_EMB_ | MA759B | IW1536 | 58 |
| *Dasyurus* | *viverrinus* | ZMB_EMB_ | MA758B | IW1537 | 61 |
| *Dasyurus* | *viverrinus* | ZMB_EMB_ | MA752A | IW1538 | 65 |
| *Dasyurus* | *viverrinus* | ZMB_EMB_ | MA726 | IW1539 | 69 |
| *Dasyurus* | *viverrinus* | ZMB_EMB_ | MA717 | IW1540 | 69 |
| *Dasyurus* | *viverrinus* | ZMB_EMB_ | MA716 | IW1541 | 89 |
| *Dasyurus* | *viverrinus* | ZMB_EMB_ | MA712A | IW1542 | 130 |
| *Dasyurus* | *viverrinus* | ZMB_EMB_ | MA712B | IW1543 | 140 |
| *Dasyurus* | *viverrinus* | ZMB_Mam_ | 72258 | IW1544 | - |
| *Phascolarctos* | *cinereus* | ZMB_EMB_ | MA487 2 | IW1545 | 32 |
| *Phascolarctos* | *cinereus* | ZMB_EMB_ | MA487 1 | IW1546 | 36 |
| *Phascolarctos* | *cinereus* | ZMB_EMB_ | MA488 | IW1547 | 41 |
| *Phascolarctos* | *cinereus* | ZMB_EMB_ | MA499 2 | IW1548 | 56 |
| *Phascolarctos* | *cinereus* | ZMB_EMB_ | MA486 | IW1549 | 47 |
| *Phascolarctos* | *cinereus* | ZMB_EMB_ | MA485a | IW1550 | 52 |
| *Phascolarctos* | *cinereus* | ZMB_EMB_ | MA497 2 | IW1551 | 58 |
| *Phascolarctos* | *cinereus* | ZMB_EMB_ | MA503a 1 | IW1552 | 42 |
| *Phascolarctos* | *cinereus* | ZMB_EMB_ | MA503a 2 | IW1553 | 44 |
| *Phascolarctos* | *cinereus* | ZMB_EMB_ | MA500b | IW1554 | 52 |
| *Phascolarctos* | *cinereus* | ZMB_EMB_ | MA499 1 | IW1555 | 53 |
| *Phascolarctos* | *cinereus* | ZMB_EMB_ | MA497 1 | IW1556 | 62 |
| *Phascolarctos* | *cinereus* | ZMB_EMB_ | MA484 | IW1557 | 67 |
| *Phascolarctos* | *cinereus* | ZMB_EMB_ | MA498 2 | IW1558 | 63 |
| *Phascolarctos* | *cinereus* | ZMB_EMB_ | MA502 | IW1559 | 64 |
| *Phascolarctos* | *cinereus* | ZMB_EMB_ | MA498 1 | IW1560 | 66 |
| *Phascolarctos* | *cinereus* | ZMB_EMB_ | MA500a | IW1561 | 70 |
| *Phascolarctos* | *cinereus* | ZMB_EMB_ | MA496 | IW1562 | 71 |
| *Phascolarctos* | *cinereus* | ZMB_EMB_ | MA495 2 | IW1563 | 80 |
| *Phascolarctos* | *cinereus* | ZMB_EMB_ | MA495 1 | IW1564 | 81 |
| *Phascolarctos* | *cinereus* | ZMB_EMB_ | MA494 2 | IW1565 | 97 |
| *Phascolarctos* | *cinereus* | ZMB_EMB_ | MA483 | IW1566 | 99 |
| *Phascolarctos* | *cinereus* | ZMB_EMB_ | MA491 | IW1567 | 168 |
| *Phascolarctos* | *cinereus* | ZMB_EMB_ | MA492 | IW1568 | 169 |
| *Phascolarctos* | *cinereus* | ZMB_EMB_ | MA506 | IW1569 | 211 |
| *Phascolarctos* | *cinereus* | ZMB_EMB_ | MA507 | IW1570 | 267 |
| *Phascolarctos* | *cinereus* | ZMB_Mam_ | 36036 | IW1571 | - |
| *Vombatus* | *ursinus* | ZMB_EMB_ | MA521a | IW1572 | 44 |
| *Vombatus* | *ursinus* | ZMB_EMB_ | MA522 | IW1573 | 49 |
| *Vombatus* | *ursinus* | ZMB_EMB_ | MA524 | IW1574 | 41 |
| *Vombatus* | *ursinus* | ZMB_EMB_ | MA518 | IW1575 | 68 |
| *Vombatus* | *ursinus* | ZMB_EMB_ | MA517 | IW1576 | 91 |
| *Vombatus* | *ursinus* | ZMB_EMB_ | MA520 | IW1577 | 89 |
| *Vombatus* | *ursinus* | ZMB_EMB_ | MA538 | IW1578 | 108 |
| *Vombatus* | *ursinus* | ZMB_EMB_ | MA519 | IW1579 | 108 |
| *Vombatus* | *ursinus* | ZMB_EMB_ | MA516 | IW1580 | 104 |
| *Vombatus* | *ursinus* | ZMB_EMB_ | MA523 | IW1581 | 137 |
| *Vombatus* | *ursinus* | ZMB_EMB_ | MA537 | IW1582 | 239 |
| *Vombatus* | *ursinus* | ZMB_Mam_ | 5702 | IW1583 | - |
| *Petrogale* | *penicillata* | ZMB_EMB_ | MA587 | IW1584 | 41 |
| *Petrogale* | *penicillata* | ZMB_EMB_ | MA583 | IW1585 | 45 |
| *Petrogale* | *penicillata* | ZMB_EMB_ | MA588 1 | IW1586 | 61 |
| *Petrogale* | *penicillata* | ZMB_EMB_ | MA577 | IW1587 | 57 |
| *Petrogale* | *penicillata* | ZMB_EMB_ | MA589 2 | IW1588 | 65 |
| *Petrogale* | *penicillata* | ZMB_EMB_ | MA588 3 | IW1589 | 57 |
| *Petrogale* | *penicillata* | ZMB_EMB_ | MA589 1 | IW1590 | 67 |
| *Petrogale* | *penicillata* | ZMB_EMB_ | MA588 2 | IW1591 | 66 |
| *Petrogale* | *penicillata* | ZMB_EMB_ | MA590 | IW1592 | 83 |
| *Petrogale* | *penicillata* | ZMB_EMB_ | MA564 | IW1593 | 82 |
| *Petrogale* | *penicillata* | ZMB_EMB_ | MA576 | IW1594 | 108 |
| *Petrogale* | *penicillata* | ZMB_EMB_ | MA585 | IW1595 | 108 |
| *Petrogale* | *penicillata* | ZMB_EMB_ | MA591 | IW1596 | 114 |
| *Petrogale* | *penicillata* | ZMB_EMB_ | MA592 | IW1597 | 123 |
| *Petrogale* | *penicillata* | ZMB_EMB_ | MA593b | IW1598 | 137 |
| *Petrogale* | *penicillata* | ZMB_EMB_ | MA593a | IW1599 | 169 |
| *Petrogale* | *penicillata* | ZMB_Mam_ | 4212 | IW1600 | - |

**Supplementary Table 2.** The relative timing of the onset of ossification of each bone for all of the studied bones and their minimum and maximum values, as well as their median and 25th and 75th percentiles. Data for the species shown in bold and with an asterisk originate from Koyabu et al. 2014. For the values of the reconstructed marsupial ancestor the CIs are given. Values in brackets mean that the values could not be reconstructed for the ground pattern of all marsupials put instead only for the last common ancestor of the species in which the bone was studied. For abbreviations of the bones, see Figure 2. The same abbreviations are used for Supplementary Tables 4-21.

|  |  | **Mx** | **De** | **Pm** | **Ju** | **Fr** | **Pl** | **Eo** | **Pa** | **Pt** | **Sq** | **Et** | **So** | **As** | **Bo** | **Vo** | **Go** | **La** | **Bs** | **Na** | **Os** | **Pe** | **Ip** | **Ml** | **Pr** | **In** |
| --- | --- | --- | --- | --- | --- | --- | --- | --- | --- | --- | --- | --- | --- | --- | --- | --- | --- | --- | --- | --- | --- | --- | --- | --- | --- | --- |
|  | **Min** | 0.08 | 0.08 | 0.08 | 0.1 | 0.1 | 0.1 | 0.1 | 0.1 | 0.1 | 0.1 | 0.1 | 0.1 | 0.1 | 0.1 | 0.2 | 0.1 | 0.23 | 0.28571 | 0.2 | 0.1 | 0.57 | 0.16667 | 0.4 | 0.625 | 0.9 |
|  | **25th Percentile** | 0.12 | 0.13 | 0.13 | 0.2 | 0.2 | 0.2 | 0.1 | 0.2 | 0.3 | 0.3 | 0.3 | 0.2 | 0.4 | 0.3 | 0.3 | 0.3 | 0.46 | 0.375 | 0.5 | 0.3 | 0.88 | 0.19167 | 0.5286 | 0.69196 | 0.975 |
|  | **Median** | 0.14 | 0.17 | 0.17 | 0.3 | 0.3 | 0.3 | 0.4 | 0.3 | 0.4 | 0.5 | 0.4 | 0.4 | 0.5 | 0.5 | 0.5 | 0.5 | 0.67 | 0.4 | 0.6 | 0.6 | 1 | 0.2875 | 0.5774 | 0.75714 | 1 |
|  | **75th Percentile** | 0.31 | 0.24 | 0.38 | 0.4 | 0.5 | 0.5 | 0.5 | 0.6 | 0.5 | 0.6 | 0.8 | 0.7 | 0.6 | 0.7 | 0.6 | 0.7 | 0.69 | 0.80556 | 0.7 | 0.9 | 1 | 0.49554 | 0.625 | 0.80833 | 1 |
|  | **Max** | 0.4 | 0.4 | 0.6 | 0.8 | 0.8 | 0.7 | 0.8 | 0.8 | 0.8 | 0.8 | 0.8 | 0.9 | 0.8 | 0.8 | 1 | 0.9 | 1 | 0.85714 | 1 | 1 | 1 | 0.85714 | 0.75 | 0.83333 | 1 |
| **Clade** | **Species** |  |  |  |  |  |  |  |  |  |  |  |  |  |  |  |  |  |  |  |  |  |  |  |  |  |
| Dasyuridae | ***Dasyurus viverrinus**** | 0.14 | 0.14 | 0.14 | 0.1 | 0.3 | 0.4 | 0.4 | 0.6 | 0.1 | 0.6 |  |  | 0.6 | 0.7 | 0.4 |  | 0.71 | 0.86 | 0.7 | 0.9 | 1 |  |  |  |  |
|  | ***Sminthopsis macroura**** | 0.11 | 0.22 | 0.33 | 0.8 | 0.7 | 0.2 | 0.4 | 0.8 | 0.6 | 0.7 | 0.8 | 0.9 | 0.8 | 0.8 | 0.8 | 0.8 | 0.78 | 0.78 | 0.8 | 1 | 1 |  |  |  |  |
| Didelphidae | ***Didelphis albiventris**** | 0.13 | 0.25 | 0.13 | 0.4 | 0.4 | 0.3 | 0.4 | 0.4 | 0.4 | 0.8 | 0.4 | 0.4 |  | 0.4 | 1 | 0.6 | 0.5 | 0.38 | 0.6 |  | 0.88 |  |  |  |  |
|  | ***Monodelphis domestica**** | 0.08 | 0.08 | 0.08 | 0.2 | 0.1 | 0.3 | 0.8 | 0.2 | 0.5 | 0.2 | 0.9 | 0.7 | 0.5 | 0.6 | 0.2 | 0.9 | 0.23 | 0.38 | 0.2 | 0.3 | 1 |  |  |  |  |
|  | ***Caluromys philander**** | 0.17 | 0.17 | 0.17 | 0.3 | 0.3 | 0.5 | 0.5 | 0.3 | 0.8 | 0.5 |  |  | 0.5 | 0.7 |  |  | 0.67 | 0.67 | 0.5 | 0.8 | 1 |  |  |  |  |
| Phalangeridae | *Trichosurus vulpecula* | 0.4 | 0.4 | 0.6 | 0.4 | 0.4 | 0.4 | 0.1 | 0.2 | 0.5 | 0.4 | 0.4 | 0.2 | 0.4 | 0.3 | 0.5 | 0.4 | 1 | 0.4 | 1 | 0.4 | 0.7 | 0.2 | 0.4 | 0.8 | 0.9 |
|  | ***Trichosurus vulpecula**** | 0.33 | 0.17 | 0.17 | 0.5 | 0.5 | 0.7 | 0.7 | 0.8 | 0.7 | 0.5 | 0.8 | 0.8 | 0.7 | 0.8 |  |  | 0.67 | 0.83 | 0.7 | 0.8 | 1 |  |  |  |  |
| Vombatiformes | *Phascolarctos cinereus* | 0.33 | 0.17 | 0.5 | 0.2 | 0.2 | 0.5 | 0.1 | 0.2 | 0.4 | 0.2 | 0.2 | 0.2 | 0.4 | 0.2 | 0.5 | 0.2 | 0.67 | 0.33 | 0.8 | 0.3 | 0.92 | 0.17 | 0.58 | 0.83 | 1 |
|  | *Vombatus ursinus* | 0.29 | 0.29 | 0.43 | 0.3 | 0.3 | 0.3 | 0.1 | 0.3 | 0.3 | 0.3 | 0.3 | 0.3 | 0.3 | 0.3 | 0.6 | 0.3 | 0.57 | 0.29 | 0.6 | 0.3 | 0.57 | 0.86 | 0.57 | 0.71 | 1 |
| Macropodidae | *Petrogale penicillata* | 0.13 | 0.13 | 0.25 | 0.1 | 0.1 | 0.1 | 0.1 | 0.1 | 0.1 | 0.1 | 0.1 | 0.1 | 0.1 | 0.1 | 0.3 | 0.1 | 0.25 | 0.38 | 0.5 | 0.1 | 0.88 | 0.38 | 0.75 | 0.63 | 1 |
|  | ***Macropus eugenii**** | 0.08 | 0.08 | 0.08 | 0.3 | 0.8 | 0.1 | 0.1 | 0.7 | 0.3 | 0.6 | 0.5 | 0.5 | 0.5 | 0.5 | 0.2 | 0.6 | 0.42 | 0.83 | 0.6 | 0.9 | 1 |  |  |  |  |
|  |  |  |  |  |  |  |  |  |  |  |  |  |  |  |  |  |  |  |  |  |  |  |  |  |  |  |
| Reconstructed ancestral value | Marsupialia | 0.19 | 0.19 | 0.25 | 0.3 | 0.3 | 0.4 | 0.4 | 0.4 | 0.4 | 0.4 | 0.5 | 0.5 | 0.5 | 0.5 | 0.5 | 0.6 | 0.58 | 0.58 | 0.6 | 0.6 | 0.92 | [0.39] | [0.58] | [0.74] | [0.97] |
|  | **70% lower CI** | -0.6 | -2.6 | -4.9 | -0.9 | -0.9 | -2 | -6.9 | -7.7 | -1.8 | 0.3 | -5.1 | -7.5 | -3.1 | -6.3 | 0.4 | 0.4 | -3.7 | -infinity | -3.6 | -4.2 | 0.83 | -infinity | -infinity | -infinity | -infinity |
|  | **70% upper CI** | 0.98 | 2.94 | 5.36 | 1.6 | 1.5 | 2.8 | 7.7 | 8.5 | 2.7 | 0.6 | 6.1 | 8.6 | 4.1 | 7.3 | 0.7 | 0.7 | 4.83 | infinity | 4.8 | 5.4 | 0.95 | infinity | infinity | infinity | infinity |

**Supplementary Table 3.** The minimum and maximum values for each bone contact and respective median and 25th and 75th percentiles, as well as the reconstructed values for the timing of bone contact of the last common ancestor of Australidelphia (last common ancestor of the studied species) and respective CIs.

| **Bone contact** | **Min** | **25th Percentile** | **Median** | **75th Percentile** | **Max** | **Reconstructed ancestral timing of bone contact** | **70% lower CI** | **70% upper CI** |
| --- | --- | --- | --- | --- | --- | --- | --- | --- |
| supraoccipital-interparietal | 0.05 | 0.14 | 0.30 | 0.42 | 0.63 | 0.29 | 0.12 | 0.46 |
| interparietal-parietal | 0.37 | 0.43 | 0.56 | 0.86 | 0.90 | 0.65 | 0.46 | 0.83 |
| parietal-frontal | 0.29 | 0.33 | 0.40 | 0.58 | 0.69 | 0.44 | 0.32 | 0.56 |
| frontal-nasal | 0.21 | 0.50 | 0.57 | 0.58 | 0.80 | 0.49 | 0.36 | 0.63 |
| squamosal-parietal | 0.25 | 0.60 | 0.62 | 0.79 | 0.79 | 0.62 | 0.47 | 0.77 |
| alisphenoid-squamosal | 0.43 | 0.50 | 0.67 | 0.68 | 0.70 | 0.58 | 0.5 | 0.65 |
| pterygoid-basisphenoid | 0.14 | 0 | 0.31 | 0 | 0.40 | 0.25 | 0.17 | 0.32 |
| alisphenoid-pterygoid | 0.29 | 0 | 0.75 | 0.80 | 0.84 | 0.55 | 0.37 | 0.74 |
| alisphenoid-basisphenoid | 0.21 | 0.29 | 0.63 | 0.80 | 1.00 | 0.54 | 0.31 | 0.78 |
| pterygoid-orbitosphenoid | 0.5 | 0.52 | 0.69 | 0.79 | 0.79 | 0.67 | 0.57 | 0.78 |
| alisphenoid-orbitosphenoid | 0.29 | 0.50 | 0.69 | 0.71 | 0.79 | 0.56 | 0.43 | 0.7 |
| basisphenoid-orbitosphenoid | 0.43 | 0.80 | 0.95 | 0.95 | 1.00 | 0.78 | 0.64 | 0.93 |
| ectotympenoid-goniale | 0.07 | 0.16 | 0.19 | 0 | 0.30 | 0.17 | 0.11 | 0.23 |
| goniale-malleus | 0.07 | 0.10 | 0 | 0.26 | 0.31 | 0.16 | 0.08 | 0.24 |
| goniale-alisphenenoid | 0.57 | 0.65 | 0.73 | 0.81 | 0.89 | 0.17 | 0.11 | 0.23 |
| vomer-palatine | 0.25 | 0.30 | 0.36 | 0.38 | 0.58 | 0.96 | 0.92 | 1 |
| vomer-orbitosphenoid | 0.68 | 0.70 | 0.75 | 0.93 | 0.95 | 0.84 | 0.74 | 0.93 |
| vomer/nasal septum-frontal | 0.95 | 0.99 | 1.00 | 1 | 1.00 | 0.99 | 0.97 | 1.01 |
| maxilla-palatine | 0.06 | 0.07 | 0.10 | 0.11 | 0.14 | 0.09 | 0.07 | 0.11 |
| orbitosphenoid-palatine | 0.38 | 0.70 | 0.79 | 0.93 | 1.00 | 0.61 | 0.28 | 0.96 |
| orbitosphenoid-maxilla | 0.10 | 0.13 | 0.14 | 0.20 | 0.53 | 0.34 | 0.17 | 0.5 |
| orbitosphenoid-frontal | 0.50 | 0.63 | 0.7 | 0.79 | 0.79 | 0.75 | 0.62 | 0.87 |
| lacrimal-maxilla | 0.19 | 0.40 | 0.5 | 0.53 | 0.57 | 0.53 | 0.5 | 0.56 |
| lacrimal-palatine | 0.48 | 0.57 | 0.74 | 0.75 | 1.00 | 0.59 | 0.47 | 0.71 |
| lacrimal-jugal | 0.25 | 0.57 | 0.63 | 0.86 | 0.90 | 0.66 | 0.48 | 0.83 |
| maxilla-jugal | 0.13 | 0.14 | 0.21 | 0.24 | 0.30 | 0.2 | 0.15 | 0.26 |
| jugal-squamosal | 0.05 | 0.13 | 0.4 | 0.50 | 0.80 | 0.39 | 0.15 | 0.63 |
| premaxilla-palatine | 0.25 | 0.60 | 0.76 | 0.79 | 0.93 | 0.69 | 0.51 | 0.87 |
| premaxilla-vomer | 0.31 | 0.79 | 0.80 | 0.93 | 1.00 | 0.78 | 0.59 | 0.98 |
| premaxilla-maxilla | 0.19 | 0.30 | 0.36 | 0.47 | 0.57 | 0.38 | 0.26 | 0.49 |
| premaxilla-nasal | 0.29 | 0.30 | 0.33 | 0.50 | 0.79 | 0.42 | 0.26 | 0.58 |
| maxilla-nasal | 0.29 | 0.67 | 0.70 | 0.75 | 0.95 | 0.63 | 0.47 | 0.78 |
| frontal-lacrimal | 0.44 | 0.62 | 0.64 | 0.74 | 0.90 | 0.84 | 0.74 | 0.93 |
| lacrimal-nasal | 0.95 | 1 | 1 | 1 | 0.95 | 0.99 | 0.97 | 1.01 |
| petrosal-exoccipital | 0.29 | 0.69 | 0.8 | 0.80 | 0.81 | 0.63 | 0.5 | 0.77 |
| petrosal-basioccipital | 0.79 | 1 | 0.80 | 0.81 | 0.81 | 0.8 | 0.79 | 0.81 |
| petrosal-squamosal | 0.71 | 0.76 | 0.79 | 0.90 | 0.94 | 0.83 | 0.75 | 0.9 |
| petrosal-parietal | 0.79 | 0.83 | 0.87 | 0.91 | 0.95 | 0.92 | 0.85 | 0.99 |
| petrosal-alisphenoid | 0.79 | 0.95 | 1.00 | 1 | 1.00 | 0.93 | 0.87 | 0.99 |
| presphenoid-orbitosphenoid | 0.71 | 0.76 | 0.79 | 0.80 | 0.94 | 0.79 | 0.73 | 0.85 |
| presphenoid-frontal | 0.76 | 0.84 | 0.86 | 0.90 | 1.00 | 0.87 | 0.81 | 0.94 |
| presphenoid-vomer | 0.76 | 0.89 | 0.93 | 1.00 | 1.00 | 0.92 | 0.84 | 0.99 |
| presphenoid-alisphenoid | 0.93 | 0.95 | 1.00 | 1 | 1.00 | 0.97 | 0.95 | 0.99 |
| presphenoid-basisphenoid | 1 | 1 | 1 | 1 | 1.00 | 1 | 1 | 1 |
| incus-malleus | 0.76 | 0.79 | 0.81 | 0.86 | 0.90 | 0.83 | 0.79 | 0.87 |
| basioccipital-basisphenoid | 0.95 | 1.00 | 1 | 1 | 1.00 | 0.99 | 0.97 | 1.01 |
| basisphenoid-petrosal | 0.86 | 1 | 1 | 1 | 0.86 | 0.6 | 0.55 | 0.7 |
| incus-petrosal | 0.76 | 0.84 | 0.88 | 0.90 | 0.93 | 0.87 | 0.72 | 0.91 |
| ectotympic-petrosal | 0.69 | 0.76 | 0.82 | 0.88 | 0.95 | 0.81 | 0.69 | 0.92 |
| frontal-maxilla | 0.81 | 0.85 | 0.88 | 0.92 | 1.00 | 0.85 | 0.82 | 0.89 |
| frontal-palatine | 0.79 | 0.81 | 0.89 | 1.00 | 1.00 | 0.88 | 0.81 | 0.96 |
| alisphenoid-frontal | 0.93 | 0.94 | 0.95 | 1 | 0.95 | 0.96 | 0.94 | 0.99 |
| alisphenoid-parietal | 0.89 | 1 | 1 | 1 | 0.89 | 0.66 | 0.55 | 0.77 |
| basioccipital-exoccipital | 0.86 | 1.00 | 1 | 1 | 1.00 | 0.87 | 0.82 | 0.91 |
| interparietal-squamosal | 0.95 | 1 | 1 | 1 | 0.95 | 0.96 | 0.92 | 1 |
| supraoccipital.-exoccipital | 0.93 | 0.95 | 1.00 | 1 | 1.00 | 0.97 | 0.95 | 0.99 |
| supraoccipital-squamosal | 0.95 | 1 | 1 | 1 | 0.95 | 0.17 | 0.11 | 0.23 |
| supraoccipital-petrosal | 1 | 1 | 1 | 1 | 1.00 | 0.96 | 0.92 | 1 |

**Supplementary Table 4.** The data matrix for the onset of ossification in *Trichosurus vulpecula*.

| **Genus** | *Trichosurus* | *Trichosurus* | *Trichosurus* | *Trichosurus* | *Trichosurus* | *Trichosurus* | *Trichosurus* | *Trichosurus* | *Trichosurus* | *Trichosurus* | *Trichosurus* | *Trichosurus* | *Trichosurus* | *Trichosurus* | *Trichosurus* | *Trichosurus* | *Trichosurus* | *Trichosurus* | *Trichosurus* | *Trichosurus* | *Trichosurus* | *Trichosurus* | *Trichosurus* | *Trichosurus* | *Trichosurus* | *Trichosurus* | *Trichosurus* | *Trichosurus* | *Trichosurus* | *Trichosurus* |
| --- | --- | --- | --- | --- | --- | --- | --- | --- | --- | --- | --- | --- | --- | --- | --- | --- | --- | --- | --- | --- | --- | --- | --- | --- | --- | --- | --- | --- | --- | --- |
| **Species** | *vulpecula* | *vulpecula* | *vulpecula* | *vulpecula* | *vulpecula* | *vulpecula* | *vulpecula* | *vulpecula* | *vulpecula* | *vulpecula* | *vulpecula* | *vulpecula* | *vulpecula* | *vulpecula* | *vulpecula* | *vulpecula* | *vulpecula* | *vulpecula* | *vulpecula* | *vulpecula* | *vulpecula* | *vulpecula* | *vulpecula* | *vulpecula* | *vulpecula* | *vulpecula* | *vulpecula* | *vulpecula* | *vulpecula* | *vulpecula* |
| **Specimen ID** | ZMB_EMB_MA374 | ZMB_EMB_MA454 | ZMB_EMB_MA362 | ZMB_EMB_MA353 | ZMB_EMB_MA381 | ZMB_EMB_MA358 | ZMB_EMB_MA396b | ZMB_EMB_MA350a | ZMB_EMB_MA377 | ZMB_EMB_MA376 | ZMB_EMB_MA378 | ZMB_EMB_MA398 | ZMB_EMB_MA453b | ZMB_EMB_MA450 | ZMB_EMB_MA452,1 | ZMB_EMB_MA379 | ZMB_EMB_MA350b | ZMB_EMB_MA402 | ZMB_EMB_MA447 | ZMB_EMB_MA401 | ZMB_EMB_MA449 | ZMB_EMB_MA452,2 | ZMB_EMB_MA400 | ZMB_EMB_MA453a | ZMB_EMB_MA397 | ZMB_EMB_MA399 | ZMB_EMB_MA444 | ZMB_EMB_MA443 | ZMB_EMB_MA467 | ZMB_Mam_13271 |
| **IW ID** | IW1500 | IW1501 | IW1502 | IW1503 | IW1504 | IW1505 | IW1506 | IW1507 | IW1508 | IW1509 | IW1510 | IW1511 | IW1512 | IW1513 | IW1514 | IW1515 | IW1516 | IW1517 | IW1518 | IW1519 | IW1520 | IW1521 | IW1522 | IW1523 | IW1524 | IW1525 | IW1526 | IW1527 | IW1528 | IW1529 |
| **Rank** | 1 | 2 | 3 |  |  | 4 | 5 |  |  |  | 6 |  | 7 | 8 | 9 | 10 |  |  |  |  |  |  |  |  |  |  |  |  |  |  |
| **SVL** | 29 | 79 | 32 | 36 | 70 | 45 | 49 | 48 | 57 | 43 | 56 | 61 | 87 | 71 | 98 | 73 | 70 | 79 | 84 | 92 | 102 | 107 | 108 | 88 | 88 | 129 | 129 | 147 | 264 | - |
| **pm** | 0 | 0 | 0 | 0 | 0 | 0 | 0 | 0 | 0 | 0 | 1 | 1 | 0 | 0 | 0 | 1 | 1 | 1 | 0 | 1 | 1 | 1 | 1 | 1 | 1 | 1 | 1 | 1 | 1 | 1 |
| **mx** | 0 | 0 | 0 | 0 | 0 | 1 | 1 | 1 | 1 | 1 | 1 | 1 | 1 | 1 | 1 | 1 | 1 | 1 | 1 | 1 | 1 | 1 | 1 | 1 | 1 | 1 | 1 | 1 | 1 | 1 |
| **de** | 0 | 0 | 0 | 0 | 0 | 1 | 1 | 1 | 1 | 1 | 1 | 1 | 1 | 1 | 1 | 1 | 1 | 1 | 1 | 1 | 1 | 1 | 1 | 1 | 1 | 1 | 1 | 1 | 1 | 1 |
| **fr** | 0 | 0 | 0 | 0 | 0 | 1 | 1 | 1 | 1 | 1 | 1 | 1 | 1 | 1 | 1 | 1 | 1 | 1 | 1 | 1 | 1 | 1 | 1 | 1 | 1 | 1 | 1 | 1 | 1 | 1 |
| **na** | 0 | 0 | 0 | 0 | 0 | 0 | 0 | 0 | 0 | 0 | 0 | 0 | 0 | 0 | 0 | 1 | 1 | 1 | 0 | 1 | 1 | 1 | 1 | 1 | 1 | 1 | 1 | 1 | 1 | 1 |
| **ju** | 0 | 0 | 0 | 0 | 0 | 1 | 1 | 1 | 1 | 1 | 1 | 1 | 1 | 1 | 1 | 1 | 1 | 1 | 1 | 1 | 1 | 1 | 1 | 1 | 1 | 1 | 1 | 1 | 1 | 1 |
| **la** | 0 | 0 | 0 | 0 | 0 | 0 | 0 | 0 | 0 | 0 | 0 | 0 | 0 | 0 | 0 | 1 | 1 | 1 | 1 | 1 | 1 | 1 | 1 | 1 | 1 | 1 | 1 | 1 | 1 | 1 |
| **pa** | 0 | 1 | 0 | 1 | 1 | 1 | 1 | 1 | 1 | 1 | 1 | 1 | 1 | 1 | 1 | 1 | 1 | 1 | 1 | 1 | 1 | 1 | 1 | 1 | 1 | 1 | 1 | 1 | 1 | 1 |
| **sq** | 0 | 0 | 0 | 0 | 0 | 1 | 1 | 1 | 1 | 1 | 1 | 1 | 1 | 1 | 1 | 1 | 1 | 1 | 1 | 1 | 1 | 1 | 1 | 1 | 1 | 1 | 1 | 1 | 1 | 1 |
| **vo** | 0 | 0 | 0 | 0 | 0 | 0 | 1 | 0 | 0 | 1 | 1 | 1 | 0 | 1 | 0 | 1 | 1 | 1 | 1 | 1 | 1 | 1 | 1 | 1 | 1 | 1 | 1 | 1 | 1 | 1 |
| **pl** | 0 | 0 | 0 | 0 | 0 | 1 | 1 | 1 | 1 | 1 | 1 | 1 | 1 | 1 | 1 | 1 | 1 | 1 | 1 | 1 | 1 | 1 | 1 | 1 | 1 | 1 | 1 | 1 | 1 | 1 |
| **os** | 0 | 0 | 0 | 0 | 0 | 1 | 1 | 1 | 1 | 1 | 1 | 1 | 1 | 1 | 1 | 1 | 1 | 1 | 1 | 1 | 1 | 1 | 1 | 1 | 1 | 1 | 1 | 1 | 1 | 1 |
| **pr** | 0 | 0 | 0 | 0 | 0 | 0 | 0 | 0 | 0 | 0 | 0 | 0 | 0 | 1 | 1 | 0 | 0 | 0 | 1 | 0 | 1 | 1 | 1 | 1 | 1 | 1 | 1 | 1 | 1 | 1 |
| **bs** | 0 | 0 | 0 | 0 | 0 | 1 | 1 | 1 | 1 | 1 | 1 | 1 | 1 | 1 | 1 | 1 | 1 | 1 | 1 | 1 | 1 | 1 | 1 | 1 | 1 | 1 | 1 | 1 | 1 | 1 |
| **pt** | 0 | 0 | 0 | 0 | 0 | 0 | 1 | 1 | 1 | 1 | 1 | 1 | 1 | 1 | 1 | 1 | 1 | 1 | 1 | 1 | 1 | 1 | 1 | 1 | 1 | 1 | 1 | 1 | 1 | 1 |
| **as** | 0 | 0 | 0 | 0 | 0 | 1 | 1 | 1 | 1 | 1 | 1 | 1 | 1 | 1 | 1 | 1 | 1 | 1 | 1 | 1 | 1 | 1 | 1 | 1 | 1 | 1 | 1 | 1 | 1 | 1 |
| **bo** | 0 | 0 | 1 | 1 | 1 | 1 | 1 | 1 | 1 | 1 | 1 | 1 | 1 | 1 | 1 | 1 | 1 | 1 | 1 | 1 | 1 | 1 | 1 | 1 | 1 | 1 | 1 | 1 | 1 | 1 |
| **so** | 0 | 1 | 1 | 1 | 1 | 1 | 1 | 1 | 1 | 1 | 1 | 1 | 1 | 1 | 1 | 1 | 1 | 1 | 1 | 1 | 1 | 1 | 1 | 1 | 1 | 1 | 1 | 1 | 1 | 1 |
| **eo** | 1 | 1 | 1 | 1 | 1 | 1 | 1 | 1 | 1 | 1 | 1 | 1 | 1 | 1 | 1 | 1 | 1 | 1 | 1 | 1 | 1 | 1 | 1 | 1 | 1 | 1 | 1 | 1 | 1 | 1 |
| **et** | 0 | 0 | 0 | 0 | 0 | 1 | 1 | 1 | 1 | 1 | 1 | 1 | 1 | 1 | 1 | 1 | 1 | 1 | 1 | 1 | 1 | 1 | 1 | 1 | 1 | 1 | 1 | 1 | 1 | 1 |
| **go** | 0 | 0 | 0 | 0 | 0 | 1 | 1 | 1 | 1 | 1 | 1 | 1 | 1 | 1 | 1 | 1 | 1 | 1 | 1 | 1 | 1 | 1 | 1 | 1 | 1 | 1 | 1 | 1 | 1 | 1 |
| **pe** | 0 | 0 | 0 | 0 | 0 | 0 | 0 | 0 | 0 | 0 | 0 | 0 | 1 | 1 | 1 | 1 | 1 | 1 | 1 | 1 | 1 | 1 | 1 | 1 | 1 | 1 | 1 | 1 | 1 | 1 |
| **ml** | 0 | 0 | 0 | 0 | 0 | 1 | 0 | 1 | 1 | 1 | 1 | 1 | 1 | 1 | 1 | 1 | 1 | 1 | 1 | 1 | 1 | 1 | 1 | 1 | 1 | 1 | 1 | 1 | 1 | 1 |
| **in** | 0 | 0 | 0 | 0 | 0 | 0 | 0 | 0 | 0 | 0 | 0 | 0 | 0 | 0 | 1 | 0 | 0 | 1 | 0 | 0 | 1 | 1 | 1 | 0 | 1 | 1 | 1 | 1 | 1 | 1 |
| **ip** | 0 | 1 | 1 | 0 | 1 | 1 | 1 | 1 | 1 | 1 | 1 | 1 | 1 | 1 | 1 | 1 | 1 | 1 | 1 | 1 | 1 | 1 | 1 | 1 | 1 | 1 | 1 | 1 | 1 | 1 |

**Supplementary Table 5.** The data matrix for the onset of ossification in *Dasyurus viverrinus*.

| **Genus** | *Dasyurus* | *Dasyurus* | *Dasyurus* | *Dasyurus* | *Dasyurus* | *Dasyurus* | *Dasyurus* | *Dasyurus* | *Dasyurus* | *Dasyurus* | *Dasyurus* | *Dasyurus* | *Dasyurus* | *Dasyurus* | *Dasyurus* |
| --- | --- | --- | --- | --- | --- | --- | --- | --- | --- | --- | --- | --- | --- | --- | --- |
| **Species** | *viverrinus* | *viverrinus* | *viverrinus* | *viverrinus* | *viverrinus* | *viverrinus* | *viverrinus* | *viverrinus* | *viverrinus* | *viverrinus* | *viverrinus* | *viverrinus* | *viverrinus* | *viverrinus* | *viverrinus* |
| **Specimen ID** | ZMB_EMB_MA759A | ZMB_EMB_MA750 | ZMB_EMB_MA752B | ZMB_EMB_MA735 | ZMB_EMB_MA758A | ZMB_EMB_MA718 | ZMB_EMB_MA759B | ZMB_EMB_MA758B | ZMB_EMB_MA752A | ZMB_EMB_MA726 | ZMB_EMB_MA717 | ZMB_EMB_MA716 | ZMB_EMB_MA712A | ZMB_EMB_MA712B | ZMB_Mam_72258 |
| **IW ID** | IW1530 | IW1531 | IW1532 | IW1533 | IW1534 | IW1535 | IW1536 | IW1537 | IW1538 | IW1539 | IW1540 | IW1541 | IW1542 | IW1543 | IW1544 |
| **Rank** | 1 | 2 | 3 |  |  |  |  |  |  |  | 4 |  |  |  |  |
| **SVL** | 45 | 51 | 65 | 55 | 57 | 72 | 58 | 61 | 65 | 69 | 69 | 89 | 130 | 140 | - |
| **pm** | 1 | 1 | 1 | 1 | 1 | 1 | 1 | 1 | 1 | 1 | 1 | 1 | 1 | 1 | 1 |
| **mx** | 1 | 1 | 1 | 1 | 1 | 1 | 1 | 1 | 1 | 1 | 1 | 1 | 1 | 1 | 1 |
| **de** | 1 | 1 | 1 | 1 | 1 | 1 | 1 | 1 | 1 | 1 | 1 | 1 | 1 | 1 | 1 |
| **fr** | 1 | 1 | 1 | 1 | 1 | 1 | 1 | 1 | 1 | 1 | 1 | 1 | 1 | 1 | 1 |
| **na** | 1 | 1 | 1 | 1 | 1 | 1 | 1 | 1 | 1 | 1 | 1 | 1 | 1 | 1 | 1 |
| **ju** | 1 | 1 | 1 | 1 | 1 | 1 | 1 | 1 | 1 | 1 | 1 | 1 | 1 | 1 | 1 |
| **la** | 1 | 1 | 1 | 1 | 1 | 1 | 1 | 1 | 1 | 1 | 1 | 1 | 1 | 1 | 1 |
| **pa** | 1 | 1 | 1 | 1 | 1 | 1 | 1 | 1 | 1 | 1 | 1 | 1 | 1 | 1 | 1 |
| **sq** | 0 | 1 | 1 | 1 | 1 | 1 | 1 | 1 | 1 | 1 | 1 | 1 | 1 | 1 | 1 |
| **vo** | 1 | 1 | 1 | 1 | 1 | 1 | 1 | 1 | 1 | 1 | 0 | 1 | 1 | 1 | 1 |
| **pl** | 1 | 1 | 1 | 1 | 1 | 1 | 1 | 1 | 1 | 1 | 1 | 1 | 1 | 1 | 1 |
| **os** | 1 | 1 | 1 | 1 | 1 | 1 | 1 | 1 | 1 | 1 | 1 | 1 | 1 | 1 | 1 |
| **pr** | 0 | 0 | 1 | 1 | 1 | 1 | 1 | 1 | 1 | 1 | 1 | 1 | 1 | 1 | 1 |
| **bs** | 1 | 1 | 1 | 1 | 1 | 1 | 1 | 1 | 1 | 1 | 1 | 1 | 1 | 1 | 1 |
| **pt** | 1 | 1 | 1 | 1 | 1 | 1 | 1 | 1 | 1 | 1 | 1 | 1 | 1 | 1 | 1 |
| **as** | 1 | 1 | 1 | 1 | 1 | 1 | 1 | 1 | 1 | 1 | 1 | 1 | 1 | 1 | 1 |
| **bo** | 1 | 1 | 1 | 1 | 1 | 1 | 1 | 1 | 1 | 1 | 1 | 1 | 1 | 1 | 1 |
| **so** | 1 | 1 | 1 | 1 | 1 | 1 | 1 | 1 | 1 | 1 | 1 | 1 | 1 | 1 | 1 |
| **eo** | 1 | 1 | 1 | 1 | 1 | 1 | 1 | 1 | 1 | 1 | 1 | 1 | 1 | 1 | 1 |
| **et** | 1 | 1 | 1 | 1 | 1 | 1 | 1 | 1 | 1 | 1 | 1 | 1 | 1 | 1 | 1 |
| **go** | 1 | 1 | 1 | 1 | 1 | 1 | 1 | 1 | 1 | 1 | 1 | 1 | 1 | 1 | 1 |
| **pe** | 0 | 0 | 1 | 1 | 1 | 1 | 1 | 1 | 1 | 1 | 1 | 1 | 1 | 1 | 1 |
| **ml** | 1 | 1 | 1 | 1 | 1 | 1 | 1 | 1 | 1 | 1 | 1 | 1 | 1 | 1 | 1 |
| **in** | 0 | 0 | 0 | 0 | 0 | 0 | 0 | 0 | 0 | 0 | 1 | 1 | 1 | 1 | 1 |
| **ip** | 1 | 1 | 1 | 1 | 1 | 1 | 1 | 1 | 1 | 1 | 1 | 1 | 1 | 1 | 1 |

**Supplementary Table 6.** The data matrix for the onset of ossification in *Phascolarctos cinereus*.

| **Genus** | *Phascolarctos* | *Phascolarctos* | *Phascolarctos* | *Phascolarctos* | *Phascolarctos* | *Phascolarctos* | *Phascolarctos* | *Phascolarctos* | *Phascolarctos* | *Phascolarctos* | *Phascolarctos* | *Phascolarctos* | *Phascolarctos* | *Phascolarctos* | *Phascolarctos* | *Phascolarctos* | *Phascolarctos* | *Phascolarctos* | *Phascolarctos* | *Phascolarctos* | *Phascolarctos* | *Phascolarctos* | *Phascolarctos* | *Phascolarctos* | *Phascolarctos* | *Phascolarctos* | *Phascolarctos* |
| --- | --- | --- | --- | --- | --- | --- | --- | --- | --- | --- | --- | --- | --- | --- | --- | --- | --- | --- | --- | --- | --- | --- | --- | --- | --- | --- | --- |
| **Species** | *cinereus* | *cinereus* | *cinereus* | *cinereus* | *cinereus* | *cinereus* | *cinereus* | *cinereus* | *cinereus* | *cinereus* | *cinereus* | *cinereus* | *cinereus* | *cinereus* | *cinereus* | *cinereus* | *cinereus* | *cinereus* | *cinereus* | *cinereus* | *cinereus* | *cinereus* | *cinereus* | *cinereus* | *cinereus* | *cinereus* | *cinereus* |
| **Specimen ID** | ZMB_EMB_MA487 2 | ZMB_EMB_MA487 1 | ZMB_EMB_MA488 | ZMB_EMB_MA499 2 | ZMB_EMB_MA486 | ZMB_EMB_MA485a | ZMB_EMB_MA497 2 | ZMB_EMB_MA503a 1 | ZMB_EMB_MA503a 2 | ZMB_EMB_MA500b | ZMB_EMB_MA499 1 | ZMB_EMB_MA497 1 | ZMB_EMB_MA484 | ZMB_EMB_MA498 2 | ZMB_EMB_MA502 | ZMB_EMB_MA498 1 | ZMB_EMB_MA500a | ZMB_EMB_MA496 | ZMB_EMB_MA495 2 | ZMB_EMB_MA495 1 | ZMB_EMB_MA494 2 | ZMB_EMB_MA483 | ZMB_EMB_MA491 | ZMB_EMB_MA492 | ZMB_EMB_MA506 | ZMB_EMB_MA507 | ZMB_Mam_36036 adult |
| **IW ID** | IW1545 | IW1546 | IW1547 | IW1548 | IW1549 | IW1550 | IW1551 | IW1552 | IW1553 | IW1554 | IW1555 | IW1556 | IW1557 | IW1558 | IW1559 | IW1560 | IW1561 | IW1562 | IW1563 | IW1564 | IW1565 | IW1566 | IW1567 | IW1568 | IW1569 | IW1570 | IW1571 |
| **Rank** | 1 |  |  | 2 | 3 | 4 | 5 | 6 |  |  |  | 7 | 8 | 9 |  | 10 |  |  |  |  | 11 |  | 12 |  |  |  |  |
| **SVL** | 32 | 36 | 41 | 56 | 47 | 52 | 58 | 42 | 44 | 52 | 53 | 62 | 67 | 63 | 64 | 66 | 70 | 71 | 80 | 81 | 97 | 99 | 168 | 169 | 211 | 267 | - |
| **pm** | 0 | 0 | 0 | 0 | 0 | 0 | 0 | 1 | 1 | 0 | 0 | 0 | 0 | 1 | 1 | 1 | 1 | 1 | 1 | 1 | 1 | 1 | 1 | 1 | 1 | 1 | 1 |
| **mx** | 0 | 0 | 0 | 0 | 0 | 1 | 1 | 1 | 1 | 1 | 1 | 1 | 1 | 1 | 1 | 1 | 1 | 1 | 1 | 1 | 1 | 1 | 1 | 1 | 1 | 1 | 1 |
| **de** | 0 | 0 | 0 | 1 | 1 | 1 | 1 | 1 | 1 | 1 | 1 | 1 | 1 | 1 | 1 | 1 | 1 | 1 | 1 | 1 | 1 | 1 | 1 | 1 | 1 | 1 | 1 |
| **fr** | 0 | 0 | 0 | 1 | 1 | 1 | 1 | 1 | 1 | 1 | 1 | 1 | 1 | 1 | 1 | 1 | 1 | 1 | 1 | 1 | 1 | 1 | 1 | 1 | 1 | 1 | 1 |
| **na** | 0 | 0 | 0 | 0 | 0 | 0 | 0 | 0 | 0 | 0 | 0 | 0 | 0 | 1 | 1 | 0 | 1 | 1 | 1 | 1 | 1 | 1 | 1 | 1 | 1 | 1 | 1 |
| **ju** | 0 | 0 | 0 | 1 | 1 | 1 | 1 | 1 | 1 | 1 | 1 | 1 | 1 | 1 | 1 | 1 | 1 | 1 | 1 | 1 | 1 | 1 | 1 | 1 | 1 | 1 | 1 |
| **la** | 0 | 0 | 0 | 0 | 0 | 0 | 0 | 0 | 0 | 0 | 0 | 0 | 1 | 1 | 1 | 1 | 1 | 1 | 1 | 1 | 1 | 1 | 1 | 1 | 1 | 1 | 1 |
| **pa** | 0 | 0 | 0 | 1 | 1 | 1 | 1 | 1 | 1 | 1 | 1 | 1 | 1 | 1 | 1 | 1 | 1 | 1 | 1 | 1 | 1 | 1 | 1 | 1 | 1 | 1 | 1 |
| **sq** | 0 | 0 | 0 | 1 | 1 | 1 | 1 | 1 | 1 | 1 | 1 | 1 | 1 | 1 | 1 | 1 | 1 | 1 | 1 | 1 | 1 | 1 | 1 | 1 | 1 | 1 | 1 |
| **vo** | 0 | 0 | 0 | 0 | 0 | 0 | 0 | 1 | 1 | 1 | 1 | 1 | 1 | 1 | 1 | 1 | 1 | 1 | 1 | 1 | 1 | 1 | 1 | 1 | 1 | 1 | 1 |
| **pl** | 0 | 0 | 0 | 0 | 0 | 0 | 0 | 1 | 1 | 1 | 1 | 1 | 1 | 1 | 1 | 1 | 1 | 1 | 1 | 1 | 1 | 1 | 1 | 1 | 1 | 1 | 1 |
| **os** | 0 | 0 | 0 | 0 | 1 | 1 | 0 | 1 | 1 | 1 | 1 | 1 | 1 | 1 | 1 | 1 | 1 | 1 | 1 | 1 | 1 | 1 | 1 | 1 | 1 | 1 | 1 |
| **pr** | 0 | 0 | 0 | 0 | 0 | 0 | 0 | 0 | 0 | 0 | 0 | 0 | 0 | 0 | 0 | 1 | 1 | 0 | 1 | 1 | 1 | 1 | 1 | 1 | 1 | 1 | 1 |
| **bs** | 0 | 0 | 0 | 0 | 0 | 1 | 1 | 0 | 1 | 1 | 1 | 1 | 1 | 1 | 1 | 1 | 1 | 1 | 1 | 1 | 1 | 1 | 1 | 1 | 1 | 1 | 1 |
| **pt** | 0 | 0 | 0 | 0 | 0 | 0 | 1 | 1 | 1 | 1 | 1 | 1 | 1 | 1 | 1 | 1 | 1 | 1 | 1 | 1 | 1 | 1 | 1 | 1 | 1 | 1 | 1 |
| **as** | 0 | 0 | 0 | 0 | 0 | 0 | 1 | 0 | 0 | 1 | 1 | 1 | 1 | 1 | 1 | 1 | 1 | 1 | 1 | 1 | 1 | 1 | 1 | 1 | 1 | 1 | 1 |
| **bo** | 0 | 0 | 0 | 1 | 1 | 1 | 1 | 1 | 1 | 1 | 1 | 1 | 1 | 1 | 1 | 1 | 1 | 1 | 1 | 1 | 1 | 1 | 1 | 1 | 1 | 1 | 1 |
| **so** | 0 | 0 | 0 | 1 | 1 | 1 | 1 | 1 | 0 | 1 | 1 | 1 | 1 | 1 | 1 | 1 | 1 | 1 | 1 | 1 | 1 | 1 | 1 | 1 | 1 | 1 | 1 |
| **eo** | 1 | 1 | 1 | 1 | 1 | 1 | 1 | 1 | 1 | 1 | 1 | 1 | 1 | 1 | 1 | 1 | 1 | 1 | 1 | 1 | 1 | 1 | 1 | 1 | 1 | 1 | 1 |
| **et** | 0 | 0 | 0 | 1 | 1 | 1 | 1 | 1 | 1 | 1 | 1 | 1 | 1 | 1 | 1 | 1 | 1 | 1 | 1 | 1 | 1 | 1 | 1 | 1 | 1 | 1 | 1 |
| **go** | 0 | 0 | 0 | 1 | 1 | 1 | 1 | 1 | 1 | 1 | 1 | 1 | 1 | 1 | 1 | 1 | 1 | 1 | 1 | 1 | 1 | 1 | 1 | 1 | 1 | 1 | 1 |
| **pe** | 0 | 0 | 0 | 0 | 0 | 0 | 0 | 0 | 0 | 0 | 0 | 0 | 0 | 0 | 0 | 0 | 0 | 0 | 0 | 0 | 1 | 1 | 1 | 1 | 1 | 1 | 1 |
| **ml** | 0 | 0 | 0 | 0 | 0 | 0 | 0 | 0 | 0 | 0 | 0 | 1 | 1 | 1 | 1 | 1 | 1 | 1 | 1 | 1 | 1 | 1 | 1 | 1 | 1 | 1 | 1 |
| **in** | 0 | 0 | 0 | 0 | 0 | 0 | 0 | 0 | 0 | 0 | 0 | 0 | 0 | 0 | 0 | 0 | 0 | 0 | 0 | 0 | 0 | 0 | 1 | 1 | 1 | 1 | 1 |
| **ip** | 0 | 0 | 0 | 1 | 1 | 0 | 1 | 0 | 0 | 0 | 1 | 1 | 1 | 1 | 1 | 1 | 1 | 1 | 1 | 1 | 1 | 1 | 1 | 1 | 1 | 1 | 1 |

**Supplementary Table 7.** The data matrix for the onset of ossification in *Vombatus ursinus*.

| **Genus** | *Vombatus* | *Vombatus* | *Vombatus* | *Vombatus* | *Vombatus* | *Vombatus* | *Vombatus* | *Vombatus* | *Vombatus* | *Vombatus* | *Vombatus* | *Vombatus* |
| --- | --- | --- | --- | --- | --- | --- | --- | --- | --- | --- | --- | --- |
| **Species** | *ursinus* | *ursinus* | *ursinus* | *ursinus* | *ursinus* | *ursinus* | *ursinus* | *ursinus* | *ursinus* | *ursinus* | *ursinus* | *ursinus* |
| **Specimen ID** | ZMB_EMB_MA521a | ZMB_EMB_MA522 | ZMB_EMB_MA524 | ZMB_EMB_MA518 | ZMB_EMB_MA517 | ZMB_EMB_MA520 | ZMB_EMB_MA538 | ZMB_EMB_MA519 | ZMB_EMB_MA516 | ZMB_EMB_MA523 | ZMB_EMB_MA537 | ZMB_Mam_5702 |
| **IW ID** | IW1572 | IW1573 | IW1574 | IW1575 | IW1576 | IW1577 | IW1578 | IW1579 | IW1580 | IW1581 | IW1582 | IW1583 |
| **Rank** |  | 1 | 2 | 3 | 4 | 5 |  | 6 |  |  | 7 |  |
| **SVL** | 44 | 49 | 41 | 68 | 91 | 89 | 108 | 108 | 104 | 137 | 239 |  |
| **pm** | 0 | 0 | 0 | 1 | 1 | 1 | 1 | 1 | 1 | 1 | 1 | 1 |
| **mx** | 0 | 0 | 1 | 1 | 1 | 1 | 1 | 1 | 1 | 1 | 1 | 1 |
| **de** | 0 | 0 | 1 | 1 | 1 | 1 | 1 | 1 | 1 | 1 | 1 | 1 |
| **fr** | 0 | 0 | 1 | 1 | 1 | 1 | 1 | 1 | 1 | 1 | 1 | 1 |
| **na** | 0 | 0 | 0 | 0 | 1 | 1 | 1 | 1 | 1 | 1 | 1 | 1 |
| **ju** | 0 | 0 | 1 | 1 | 1 | 1 | 1 | 1 | 1 | 1 | 1 | 1 |
| **la** | 0 | 0 | 0 | 0 | 1 | 1 | 1 | 1 | 1 | 1 | 1 | 1 |
| **pa** | 0 | 0 | 1 | 0 | 1 | 1 | 1 | 1 | 1 | 1 | 1 | 1 |
| **sq** | 0 | 0 | 1 | 1 | 1 | 1 | 1 | 1 | 1 | 1 | 1 | 1 |
| **vo** | 0 | 0 | 0 | 0 | 1 | 1 | 1 | 1 | 1 | 1 | 1 | 1 |
| **pl** | 0 | 0 | 1 | 1 | 1 | 1 | 1 | 1 | 1 | 1 | 1 | 1 |
| **os** | 0 | 0 | 1 | 1 | 1 | 1 | 1 | 1 | 1 | 1 | 1 | 1 |
| **pr** | 0 | 0 | 0 | 0 | 0 | 1 | 1 | 1 | 1 | 1 | 1 | 1 |
| **bs** | 0 | 0 | 1 | 1 | 1 | 1 | 1 | 1 | 1 | 1 | 1 | 1 |
| **pt** | 0 | 0 | 1 | 1 | 1 | 1 | 1 | 1 | 1 | 1 | 1 | 1 |
| **as** | 0 | 0 | 1 | 1 | 1 | 1 | 1 | 1 | 1 | 1 | 1 | 1 |
| **bo** | 0 | 0 | 1 | 0 | 1 | 1 | 1 | 1 | 1 | 1 | 1 | 1 |
| **so** | 0 | 0 | 1 | 0 | 1 | 1 | 1 | 1 | 1 | 1 | 1 | 1 |
| **eo** | 0 | 1 | 1 | 0 | 1 | 1 | 1 | 1 | 1 | 1 | 1 | 1 |
| **et** | 0 | 0 | 1 | 0 | 1 | 1 | 1 | 1 | 1 | 1 | 1 | 1 |
| **go** | 0 | 0 | 1 | 0 | 1 | 1 | 1 | 1 | 1 | 1 | 1 | 1 |
| **pe** | 0 | 0 | 0 | 0 | 1 | 1 | 1 | 1 | 1 | 1 | 1 | 1 |
| **ml** | 0 | 0 | 0 | 0 | 1 | 1 | 1 | 1 | 1 | 1 | 1 | 1 |
| **in** | 0 | 0 | 0 | 0 | 0 | 0 | 0 | 0 | 0 | 0 | 1 | 1 |
| **ip** | 0 | 0 | 0 | 0 | 0 | 0 | 0 | 1 | 1 | 1 | 1 | 1 |

**Supplementary Table 8.** The data matrix for the onset of ossification in *Petrogale penicillata*.

| **Genus** | *Petrogale* | *Petrogale* | *Petrogale* | *Petrogale* | *Petrogale* | *Petrogale* | *Petrogale* | *Petrogale* | *Petrogale* | *Petrogale* | *Petrogale* | *Petrogale* | *Petrogale* | *Petrogale* | *Petrogale* | *Petrogale* | *Petrogale* |
| --- | --- | --- | --- | --- | --- | --- | --- | --- | --- | --- | --- | --- | --- | --- | --- | --- | --- |
| **Species** | *penicillata* | *penicillata* | *penicillata* | *penicillata* | *penicillata* | *penicillata* | *penicillata* | *penicillata* | *penicillata* | *penicillata* | *penicillata* | *penicillata* | *penicillata* | *penicillata* | *penicillata* | *penicillata* | *penicillata* |
| **Specimen ID** | ZMB_EMB_MA587 | ZMB_EMB_MA583 | ZMB_EMB_MA588 1 | ZMB_EMB_MA577 | ZMB_EMB_MA589 2 | ZMB_EMB_MA588 3 | ZMB_EMB_MA589 1 | ZMB_EMB_MA588 2 | ZMB_EMB_MA590 | ZMB_EMB_MA564 | ZMB_EMB_MA576 | ZMB_EMB_MA585 | ZMB_EMB_MA591 | ZMB_EMB_MA592 | ZMB_EMB_MA593b | ZMB_EMB_MA593a | ZMB_Mam_4212 |
| **IW ID** | IW1584 | IW1585 | IW1586 | IW1587 | IW1588 | IW1589 | IW1590 | IW1591 | IW1592 | IW1593 | IW1594 | IW1595 | IW1596 | IW1597 | IW1598 | IW1599 | IW1600 |
| **Rank** | 1 | 2 | 3 |  | 4 | 5 | 6 | 7 |  |  | 8 |  |  |  |  |  |  |
| **SVL** | 41 | 45 | 61 | 57 | 65 | 57 | 67 | 66 | 83 | 82 | 108 | 108 | 114 | 123 | 137 | 169 |  |
| **pm** | 0 | 1 | 1 | 1 | 1 | 1 | 1 | 1 | 1 | 1 | 1 | 1 | ? | 1 | 1 | 1 | 1 |
| **mx** | 1 | 1 | 1 | 1 | 1 | 1 | 1 | 1 | 1 | 1 | 1 | 1 | 1 | 1 | 1 | 1 | 1 |
| **de** | 1 | 1 | 1 | 1 | 1 | 1 | 1 | 1 | 1 | 1 | 1 | 1 | 1 | 1 | 1 | 1 | 1 |
| **fr** | 1 | 1 | 1 | 1 | 1 | 1 | 1 | 1 | 1 | 1 | 1 | 1 | 1 | 1 | 1 | 1 | 1 |
| **na** | 0 | 0 | 0 | 0 | 1 | 1 | 1 | 1 | 1 | 1 | 1 | 1 | ? | 1 | 1 | 1 | 1 |
| **ju** | 1 | 1 | 1 | 1 | 1 | 1 | 1 | 1 | 1 | 1 | 1 | 1 | 1 | 1 | 1 | 1 | 1 |
| **la** | 0 | 1 | 1 | 1 | 1 | 1 | 1 | 1 | 1 | 1 | 1 | 1 | 1 | 1 | 1 | 1 | 1 |
| **pa** | 1 | 1 | 1 | 1 | 1 | 1 | 1 | 1 | 1 | 1 | 1 | 1 | 1 | 1 | 1 | 1 | 1 |
| **sq** | 1 | 1 | 1 | 1 | 1 | 1 | 1 | 1 | 1 | 1 | 1 | 1 | 1 | 1 | 1 | 1 | 1 |
| **vo** | 0 | 1 | 1 | 1 | 1 | 1 | 1 | 1 | 1 | 1 | 1 | 1 | ? | 1 | 1 | 1 | 1 |
| **pl** | 1 | 1 | 1 | 1 | 1 | 1 | 1 | 1 | 1 | 1 | 1 | 1 | 1 | 1 | 1 | 1 | 1 |
| **os** | 1 | 1 | 1 | 1 | 1 | 1 | 1 | 1 | 1 | 1 | 1 | 1 | 1 | 1 | 1 | 1 | 1 |
| **pr** | 0 | 0 | 0 | 0 | 0 | 1 | 1 | 1 | 1 | 1 | 1 | 1 | 1 | 1 | 1 | 1 | 1 |
| **bs** | 0 | 0 | 1 | 1 | 1 | 1 | 1 | 1 | 1 | 1 | 1 | 1 | 1 | 1 | 1 | 1 | 1 |
| **pt** | 1 | 1 | 1 | 1 | 1 | 1 | 1 | 1 | 1 | 1 | 1 | 1 | 1 | 1 | 1 | 1 | 1 |
| **as** | 1 | 1 | 1 | 1 | 1 | 1 | 1 | 1 | 1 | 1 | 1 | 1 | 1 | 1 | 1 | 1 | 1 |
| **bo** | 1 | 1 | 1 | 1 | 1 | 1 | 1 | 1 | 1 | 1 | 1 | 1 | 1 | 1 | 1 | 1 | 1 |
| **so** | 1 | 1 | 1 | 1 | 1 | 1 | 1 | 1 | 1 | 1 | 1 | 1 | 1 | 1 | 1 | 1 | 1 |
| **eo** | 1 | 1 | 1 | 1 | 1 | 1 | 1 | 1 | 1 | 1 | 1 | 1 | 1 | 1 | 1 | 1 | 1 |
| **et** | 1 | 1 | 1 | 1 | 1 | 1 | 1 | 1 | 1 | 1 | 1 | 1 | 1 | 1 | 1 | 1 | 1 |
| **go** | 1 | 1 | 1 | 1 | 1 | 1 | 1 | 1 | 1 | 1 | 1 | 1 | 1 | 1 | 1 | 1 | 1 |
| **pe** | 0 | 0 | 0 | 1? | 0 | 0 | 0 | 1 | 1 | 1 | 1 | 1 | 1 | 1 | 1 | 1 | 1 |
| **ml** | 0 | 0 | 0 | 0 | 0 | 0 | 1 | 0 | 1 | 1 | 1 | 1 | 1 | 1 | 1 | 1 | 1 |
| **in** | 0 | 0 | 0 | 0 | 0 | 0 | 0 | 0 | 0 | 0 | 1 | 1 | 1 | 1 | 1 | 1 | 1 |
| **ip** | 0 | 0 | 1 | 1 | 1 | 1 | 1 | 1 | 1 | 1 | 1 | 1 | 1 | 1 | 1 | 1 | 1 |

**Supplementary Table 9.** Table showing the ranking of all of the individual bones for the onset of ossification.

| **Ranking** | **pm** | **mx** | **de** | **fr** | **na** | **ju** | **la** | **pa** | **sq** | **vo** | **pl** | **os** | **pr** | **bs** | **pt** | **as** | **bo** | **so** | **eo** | **et** | **go** | **pe** | **ml** | **in** | **ip** |
| --- | --- | --- | --- | --- | --- | --- | --- | --- | --- | --- | --- | --- | --- | --- | --- | --- | --- | --- | --- | --- | --- | --- | --- | --- | --- |
| *T. vulpecula* | 6 | 4 | 4 | 4 | 10 | 4 | 10 | 2 | 4 | 5 | 4 | 4 | 8 | 4 | 5 | 4 | 3 | 2 | 1 | 4 | 4 | 7 | 4 | 9 | 2 |
| *D. viverrinus* | 1 | 1 | 1 | 1 | 1 | 1 | 1 | 1 | 2 | 1 | 1 | 1 | 3 | 1 | 1 | 1 | 1 | 1 | 1 | 1 | 1 | 3 | 1 | 4 | 1 |
| *Ph. cinereus* | 6 | 4 | 2 | 2 | 9 | 2 | 8 | 2 | 2 | 6 | 6 | 3 | 10 | 4 | 5 | 5 | 2 | 2 | 1 | 2 | 2 | 11 | 7 | 12 | 2 |
| *V. ursinus* | 3 | 2 | 2 | 2 | 4 | 2 | 4 | 2 | 2 | 4 | 2 | 2 | 5 | 2 | 2 | 2 | 2 | 2 | 1 | 2 | 2 | 4 | 4 | 7 | 6 |
| *Pe. penicillata* | 2 | 1 | 1 | 1 | 4 | 1 | 2 | 1 | 1 | 2 | 1 | 1 | 5 | 3 | 1 | 1 | 1 | 1 | 1 | 1 | 1 | 7 | 6 | 8 | 3 |

**Supplementary Table 10.** Table showing the relative timing of

all of the individual bones for the onset of ossification.

| **Relative timing** | **pm** | **mx** | **de** | **fr** | **na** | **ju** | **la** | **pa** | **sq** | **vo** | **pl** | **os** | **pr** | **bs** | **pt** | **as** | **bo** | **so** | **eo** | **et** | **go** | **pe** | **ml** | **in** | **ip** |
| --- | --- | --- | --- | --- | --- | --- | --- | --- | --- | --- | --- | --- | --- | --- | --- | --- | --- | --- | --- | --- | --- | --- | --- | --- | --- |
| *T. vulpecula* | 1 | 0.4 | 0.4 | 0.4 | 1 | 0.4 | 1 | 0.2 | 0.4 | 0.5 | 0.4 | 0.4 | 0.8 | 0 | 0.5 | 0.4 | 0.3 | 0.2 | 0.1 | 0.4 | 0.4 | 0.7 | 0.4 | 1 | 0.2 |
| *D. viverrinus* | 0 | 0.3 | 0.3 | 0.3 | 0 | 0.3 | 0 | 0.3 | 0.5 | 0.3 | 0.3 | 0.3 | 0.8 | 0 | 0.3 | 0.3 | 0.3 | 0.3 | 0.3 | 0.3 | 0.3 | 0.8 | 0.3 | 1 | 0.3 |
| *Ph. cinereus* | 1 | 0.3 | 0.2 | 0.2 | 1 | 0.2 | 0 | 0.2 | 0.2 | 0.5 | 0.5 | 0.3 | 0.8 | 0 | 0.4 | 0.4 | 0.2 | 0.2 | 0.1 | 0.2 | 0.2 | 0.9 | 0.6 | 1 | 0.2 |
| *V. ursinus* | 0 | 0.3 | 0.3 | 0.3 | 1 | 0.3 | 1 | 0.3 | 0.3 | 0.6 | 0.3 | 0.3 | 0.7 | 0 | 0.3 | 0.3 | 0.3 | 0.3 | 0.1 | 0.3 | 0.3 | 0.6 | 0.6 | 1 | 0.9 |
| *Pe. penicillata* | 0 | 0.13 | 0.13 | 0.13 | 1 | 0.13 | 0 | 0.13 | 0.13 | 0.3 | 0.13 | 0.13 | 0.6 | 0 | 0.13 | 0.13 | 0.13 | 0.13 | 0.13 | 0.13 | 0.13 | 0.9 | 0.8 | 1 | 0.4 |

**Supplementary Table 11.** The data matrix for the onset of bone contact in *Trichosurus vulpecula*.

| **Genus** | *Trichosurus* | *Trichosurus* | *Trichosurus* | *Trichosurus* | *Trichosurus* | *Trichosurus* | *Trichosurus* | *Trichosurus* | *Trichosurus* | *Trichosurus* | *Trichosurus* | *Trichosurus* | *Trichosurus* | *Trichosurus* | *Trichosurus* | *Trichosurus* | *Trichosurus* | *Trichosurus* | *Trichosurus* | *Trichosurus* | *Trichosurus* | *Trichosurus* | *Trichosurus* | *Trichosurus* | *Trichosurus* | *Trichosurus* | *Trichosurus* | *Trichosurus* | *Trichosurus* | *Trichosurus* |
| --- | --- | --- | --- | --- | --- | --- | --- | --- | --- | --- | --- | --- | --- | --- | --- | --- | --- | --- | --- | --- | --- | --- | --- | --- | --- | --- | --- | --- | --- | --- |
| **Species** | *vulpecula* | *vulpecula* | *vulpecula* | *vulpecula* | *vulpecula* | *vulpecula* | *vulpecula* | *vulpecula* | *vulpecula* | *vulpecula* | *vulpecula* | *vulpecula* | *vulpecula* | *vulpecula* | *vulpecula* | *vulpecula* | *vulpecula* | *vulpecula* | *vulpecula* | *vulpecula* | *vulpecula* | *vulpecula* | *vulpecula* | *vulpecula* | *vulpecula* | *vulpecula* | *vulpecula* | *vulpecula* | *vulpecula* | *vulpecula* |
| **Specimen ID** | ZMB_EMB_MA374 | ZMB_EMB_MA454 | ZMB_EMB_MA362 | ZMB_EMB_MA353 | ZMB_EMB_MA381 | ZMB_EMB_MA452,1 | ZMB_EMB_MA396b | ZMB_EMB_MA378 | ZMB_EMB_MA358 | ZMB_EMB_MA453b | ZMB_EMB_MA376 | ZMB_EMB_MA401 | ZMB_EMB_MA449 | ZMB_EMB_MA398 | ZMB_EMB_MA377 | ZMB_EMB_MA450 | ZMB_EMB_MA350a | ZMB_EMB_MA379 | ZMB_EMB_MA452,2 | ZMB_EMB_MA402 | ZMB_EMB_MA447 | ZMB_EMB_MA400 | ZMB_EMB_MA350b | ZMB_EMB_MA397 | ZMB_EMB_MA443 | ZMB_EMB_MA453a | ZMB_EMB_MA399 | ZMB_EMB_MA444 | ZMB_EMB_MA467 | ZMB_Mam_13271 |
| **IW ID** | IW1500 | IW1501 | IW1502 | IW1503 | IW1504 | IW1514 | IW1506 | IW1510 | IW1505 | IW1512 | IW1509 | IW1519 | IW1520 | IW1511 | IW1508 | IW1513 | IW1507 | IW1515 | IW1521 | IW1517 | IW1518 | IW1522 | IW1516 | IW1524 | IW1527 | IW1523 | IW1525 | IW1526 | IW1528 | IW1529 |
| **Rank** |  |  |  |  | 1 | 2 | 3 | 4 | 5 | 6 |  | 7 |  | 8 | 9 | 10 | 11 | 12 |  |  | 13 | 14 | 15 |  | 16 | 17 | 18 | 19 | 20 | 21 |
| **so-ip** | 0 | 0 | 0 | 0 | 2 | 0 | 0 | 0 | 0 | 0 | 0 | 0 | 0 | 0 | 0 | 0 | 2 | 2 | 2 | 3 | 2 | 3 | 3 | 3 | 3 | 0 | 4 | 3 | 4 | 4 |
| **ip-pa** | 0 | 0 | 0 | 0 | 0 | 0 | 0 | 0 | 0 | 0 | 0 | 0 | 0 | 0 | 2 | 2 | 0 | 3 | 2 | 2 | 2 | 0 | 3 | 2 | 2 | 2 | 4 | 4 | 4 | 4 |
| **pa-fr** | 0 | 0 | 0 | 0 | 0 | 0 | 0 | 0 | 0 | 0 | 0 | 0 | 2 | 0 | 0 | 3 | 0 | 2 | 3 | 0 | 3 | 3 | 3 | 3 | 0 | 2 | 3 | 3 | 3.5 | 4 |
| **fr-na** | 0 | 0 | 0 | 0 | 0 | 0 | 0 | 0 | 0 | 0 | 0 | 0 | 0 | 0 | 0 | 0 | 0 | 2 | 2 | 2 | 0 | 3 | 3 | 3 | 2 | 3 | 4 | 4 | 4 | 4 |
| **sq-pa** | 0 | 0 | 0 | 0 | 0 | 0 | 0 | 0 | 0 | 0 | 0 | 0 | 0 | 0 | 0 | 0 | 0 | 0 | 0 | 0 | 2 | 2.5 | 2 | 3 | 3 | 3 | 3 | 4 | 4 | 4 |
| **as-sq** | 0 | 0 | 0 | 0 | 0 | 0 | 0 | 0 | 0 | 0 | 0 | 0 | 0 | 0 | 0 | 0 | 0 | 0 | 0 | 0 | 0 | 2 | 0 | 2 | 4 | 2 | 3 | 3 | 4 | 4 |
| **pt-bs** | 0 | 0 | 0 | 0 | 0 | 0 | 2 | 2 | 0 | 0 | 0 | 2 | 0 | 3 | 0 | 0 | 0 | 3 | 0 | 2 | 2 | 3 | 3 | 3 | 0? | 3 | 0 | 4 | 4 | 4 |
| **as-pt** | 0 | 0 | 0 | 0 | 0 | 0 | 0 | 0 | 0 | 2 | 0 | 0 | 2 | 0 | 0 | 2 | 0 | 0 | 2 | 2 | 2 | 3 | 2 | 3 | 2? | 3 | 4 | 4 | 4 | 4 |
| **as-bs** | 0 | 0 | 0 | 0 | 0 | 0 | 0 | 0 | 0 | 3 | 0 | 0 | 0 | 0 | 0 | 0 | 0 | 0 | 0 | 0 | 0 | 0 | 0 | 0 | 3 | 0 | 0 | 3 | 4 | 4 |
| **pt-os** | 0 | 0 | 0 | 0 | 0 | 0 | 0 | 0 | 0 | 0 | 0 | 0 | 0 | 0 | 0 | 0 | 3 | 0 | 0 | 0 | 0 | 0 | 0 | 2 | 0 | 3 | 2 | 0? | ? | 4 |
| **as-os** | 0 | 0 | 0 | 0 | 0 | 0 | 0 | 0 | 0 | 0 | 0 | 0 | 0 | 0 | 0 | 0 | 0 | 0 | 0 | 0 | 0 | 0 | 2 | 0 | 0 | 3 | 3 | 0? | 3.5 | 4 |
| **bs-os** | 0 | 0 | 0 | 0 | 0 | 0 | 0 | 0 | 0 | 0 | 0 | 0 | 0 | 0 | 0 | 0 | 0 | 0 | 0 | 0 | 0 | 0 | 0 | 0 | 0 | 0 | 0 | 0 | 2 | 4 |
| **et-go** | 0 | 0 | 0 | 0 | 0 | 0 | 0 | 3 | 0 | 3 | 2 | 2 | 2 | 3 | 4 | 4 | 4 | 4 | 4 | 4 | 4 | 4 | 4 | 4 | 4 | 4 | 4 | 4 | 0 | 0 |
| **go-ml** | 0 | 0 | 0 | 0 | 0 | 2 | 0 | 0 | 4 | 0 | 2 | 3 | 3 | 3 | 4 | 4 | 3 | 4 | 4 | 4 | 4 | 4 | 4 | 4 | 4 | 4 | 4 | 4 | ? | 4 |
| **go-pe** | 0 | 0 | 0 | 0 | 0 | 0 | 0 | 0 | 0 | 0 | 0 | 0 | 0 | 0 | 0 | 0 | 0 | 0 | 0 | 0 | 0 | 0 | 0 | 0 | 0 | 0 | 0 | 0 | 0 | 0 |
| **go-as** | 0 | 0 | 0 | 0 | 0 | 0 | 0 | 0 | 0 | 0 | 0 | 0 | 0 | 0 | 0 | 0 | 0 | 0 | 0 | 0 | 0 | 0 | 0 | 0 | 0 | 0 | 0 | 0 | 0 | 0 |
| **vo-pl** | 0 | 0 | 0 | 0 | 0 | 0 | 0 | 0 | 0 | 0 | 0 | 0 | 0 | 2 | 0 | 0 | 0 | 0 | 0 | 2 | 2 | 2 | 3 | 3 | 0 | 3 | 3 | 3 | 3? | 4 |
| **vo-os** | 0 | 0 | 0 | 0 | 0 | 0 | 0 | 0 | 0 | 0 | 0 | 0 | 0 | 0 | 0 | 0 | 0 | 0 | 0 | 0 | 0 | 0 | 0 | 0 | 0 | 0 | 0 | 0 | 4 | 4 |
| **vo-fr(na spt)** | 0 | 0 | 0 | 0 | 0 | 0 | 0 | 0 | 0 | 0 | 0 | 0 | 0 | 0 | 0 | 0 | 0 | 0 | 0 | 0 | 0 | 0 | 0 | 0 | 0 | 0 | 0 | 0 | 4 | 4 |
| **mx-pl** | 0 | 0 | 0 | 0 | 0 | 0 | 3 | 3 | 3 | 0 | 3 | 4 | 4 | 4 | 3 | 4 | 3 | 4 | 3 | 4 | 4 | 4 | 4 | 4 | 4 | 4 | 4 | 4 | 4 | 4 |
| **os-pl** | 0 | 0 | 0 | 0 | 0 | 0 | 0 | 0 | 0 | 0 | 0 | 0 | 0 | 0 | 0 | 0 | 0 | 0 | 0 | 0 | 0 | 0 | 0 | 0 | 0 | 0 | 0 | 0 | 0 | 4 |
| **mx-os** | 0 | 0 | 0 | 0 | 0 | 2 | 0 | 0 | 0 | 2 | 2 | 0 | 2 | 0 | 0 | 2 | 3 | 2 | 2 | 2.5 | 3 | 2 | 2.5 | 2.5 | 0 | 2 | 2 | 3 | 4 | 4 |
| **os-fr** | 0 | 0 | 0 | 0 | 0 | 0 | 0 | 0 | 0 | 0 | 0 | 0 | 0 | 0 | 0 | 0 | 0 | 0 | 0 | 0 | 0 | 2 | 0 | 3 | 2.5 | 2 | 2 | 3 | 3? | 4 |
| **la-mx** | 0 | 0 | 0 | 0 | 0 | 0 | 0 | 0 | 0 | 0 | 0 | 0 | 0 | 0 | 0 | 0 | 0 | 2 | 0 | 2 | 3 | 2 | 2 | 2 | 2 | 2 | 3 | 4 | 4 | 4 |
| **la-pl** | 0 | 0 | 0 | 0 | 0 | 0 | 0 | 0 | 0 | 0 | 0 | 0 | 0 | 0 | 0 | 2.5 | 2 | 0 | 0 | 0 | 0 | 2.5 | 0 | 2 | 2 | 2 | 2 | 3.5 | 3 | 4 |
| **la-ju** | 0 | 0 | 0 | 0 | 0 | 0 | 0 | 0 | 0 | 0 | 0 | 0 | 0 | 0 | 0 | 0 | 0 | 0 | 2 | 0 | 0 | 0 | 2 | 2 | 0 | 0 | 0 | 0 | 4 | 4 |
| **mx-ju** | 0 | 0 | 0 | 0 | 0 | 0 | 0 | 0 | 2 | 0 | 2 | 2 | 0 | 2 | 3 | 2 | 4 | 3 | 2 | 3 | 3 | 2 | 3 | 3.5 | 2.5 | 2 | 3 | 4 | 4 | 4 |
| **ju-sq** | 0 | 0 | 0 | 0 | 0 | 0 | 0 | 0 | 0 | 0 | 0 | 0 | 0 | 0 | 2 | 0 | 2 | 0 | 2 | 0 | 0 | 0 | 2 | 2 | 2 | 2 | 2 | 0 | 3 | 4 |
| **et-de** | 0 | 0 | 0 | 0 | 0 | 0 | 0 | 0 | 0 | 0 | 0 | 0 | 0 | 0 | 0 | 0 | 2 | 0 | 0 | 0 | 0 | 0 | 0 | 0 | 0 | 0 | 0 | 0 | 0 | 0 |
| **pm-pl** | 0 | 0 | 0 | 0 | 0 | 0 | 0 | 0 | 0 | 0 | 0 | 0 | 0 | 0 | 0 | 0 | 0 | 0 | 0 | 0 | 0 | 0 | 0 | 0 | 2 | 0 | 0 | 0 | 4 | 4 |
| **pm-vo** | 0 | 0 | 0 | 0 | 0 | 0 | 0 | 0 | 0 | 0 | 0 | 0 | 0 | 0 | 0 | 0 | 0 | 0 | 0 | 0 | 0 | 0 | 0 | 0 | 0 | 0 | 0 | 0 | 0? | 4 |
| **pm-mx** | 0 | 0 | 0 | 0 | 0 | 0 | 0 | 0 | 0 | 0 | 0 | 0 | 0 | 0 | 0 | 0 | 0 | 0 | 2 | 0 | 0 | 0 | 3 | 2 | 0 | 2 | 0 | 2 | 2.5? | 4 |
| **pm-na** | 0 | 0 | 0 | 0 | 0 | 0 | 0 | 0 | 0 | 0 | 0 | 2 | 0 | 0 | 0 | 0 | 0 | 0 | 0 | 2 | 0 | 3 | 2 | 3 | 3 | 3 | 4 | 2 | 4 | 4 |
| **mx-na** | 0 | 0 | 0 | 0 | 0 | 0 | 0 | 0 | 0 | 0 | 0 | 0 | 0 | 0 | 0 | 0 | 0 | 0 | 0 | 0 | 0 | 2 | 0 | 2 | 0 | 2 | 4 | 2 | 4 | 4 |
| **fr-la** | 0 | 0 | 0 | 0 | 0 | 0 | 0 | 0 | 0 | 0 | 0 | 0 | 0 | 0 | 0 | 0 | 0 | 0 | 0 | 0 | 2 | 0 | 0 | 0 | 2 | 0 | 0 | 3 | 4 | 4 |
| **la-na** | 0 | 0 | 0 | 0 | 0 | 0 | 0 | 0 | 0 | 0 | 0 | 0 | 0 | 0 | 0 | 0 | 0 | 0 | 0 | 0 | 0 | 0 | 0 | 0 | 0 | 0 | 0 | 0 | 4 | 4 |
| **pe-eo** | 0 | 0 | 0 | 0 | 0 | 0 | 0 | 0 | 0 | 0 | 0 | 0 | 0 | 0 | 0 | 0 | 0 | 0 | 0 | 0 | 0 | 0 | 0 | 0 | 0 | 2 | 2 | 3 | 3.5 | 4 |
| **pe-bo** | 0 | 0 | 0 | 0 | 0 | 0 | 0 | 0 | 0 | 0 | 0 | 0 | 0 | 0 | 0 | 0 | 0 | 0 | 0 | 0 | 0 | 0 | 0 | 0 | 0 | 2 | 3 | 2 | 0 | 4 |
| **pe-sq** | 0 | 0 | 0 | 0 | 0 | 0 | 0 | 0 | 0 | 0 | 0 | 0 | 0 | 0 | 0 | 0 | 0 | 0 | 0 | 0 | 0 | 0 | 0 | 0 | 2.5? | 0 | 3 | 3 | 4 | 4 |
| **pe-pa** | 0 | 0 | 0 | 0 | 0 | 0 | 0 | 0 | 0 | 0 | 0 | 0 | 0 | 0 | 0 | 0 | 0 | 0 | 0 | 0 | 0 | 0 | 0 | 0 | 0 | 0 | 0 | 0 | 0 | 0 |
| **pe-as** | 0 | 0 | 0 | 0 | 0 | 0 | 0 | 0 | 0 | 0 | 0 | 0 | 0 | 0 | 0 | 0 | 0 | 0 | 0 | 0 | 0 | 0 | 0 | 0 | 0 | 0 | 0 | 0 | 2 | 4 |
| **pr-os** | 0 | 0 | 0 | 0 | 0 | 0 | 0 | 0 | 0 | 0 | 0 | 0 | 0 | 0 | 0 | 0 | 0 | 0 | 0 | 0 | 0 | 0 | 0 | 0 | 3? | 0 | 0 | 2 | 0 | 4 |
| **pr-fr** | 0 | 0 | 0 | 0 | 0 | 0 | 0 | 0 | 0 | 0 | 0 | 0 | 0 | 0 | 0 | 0 | 0 | 0 | 0 | 0 | 0 | 0 | 0 | 0 | 2.5 | 0 | 3 | 4 | 4? | 4 |
| **pr-vo** | 0 | 0 | 0 | 0 | 0 | 0 | 0 | 0 | 0 | 0 | 0 | 0 | 0 | 0 | 0 | 0 | 0 | 0 | 0 | 0 | 0 | 0 | 0 | 0 | 3? | 0 | 0 | 2 | 2.5? | 4 |
| **pr-as** | 0 | 0 | 0 | 0 | 0 | 0 | 0 | 0 | 0 | 0 | 0 | 0 | 0 | 0 | 0 | 0 | 0 | 0 | 0 | 0 | 0 | 0 | 0 | 0 | 0 | 0 | 0 | 0 | 0? | 4 |
| **pr-bs** | 0 | 0 | 0 | 0 | 0 | 0 | 0 | 0 | 0 | 0 | 0 | 0 | 0 | 0 | 0 | 0 | 0 | 0 | 0 | 0 | 0 | 0 | 0 | 0 | 0 | 0 | 0 | 0 | 0 | 2.5 |
| **in-ml** | 0 | 0 | 0 | 0 | 0 | 0 | 0 | 0 | 0 | 0 | 0 | 0 | 0 | 0 | 0 | 0 | 0 | 0 | 0 | 0 | 0 | 0 | 0 | 0 | 3? | 4? | 3? | 4 | 3? | 4 |
| **bo-bs** | 0 | 0 | 0 | 0 | 0 | 0 | 0 | 0 | 0 | 0 | 0 | 0 | 0 | 0 | 0 | 0 | 0 | 0 | 0 | 0 | 0 | 0 | 0 | 0 | 0 | 0 | 0 | 0 | 0 | 4 |
| **bs-pe** | 0 | 0 | 0 | 0 | 0 | 0 | 0 | 0 | 0 | 0 | 0 | 0 | 0 | 0 | 0 | 0 | 0 | 0 | 0 | 0 | 0 | 0 | 0 | 0 | 0 | 0 | 0 | 0 | 0 | 0 |
| **et-sq** | 0 | 0 | 0 | 0 | 0 | 0 | 0 | 0 | 0 | 0 | 0 | 0 | 0 | 0 | 0 | 0 | 0 | 0 | 0 | 0 | 0 | 0 | 0 | 0 | 0 | 0 | 0 | 2 | 0 | 0 |
| **as-et** | 0 | 0 | 0 | 0 | 0 | 0 | 0 | 0 | 0 | 0 | 0 | 0 | 0 | 0 | 0 | 0 | 0 | 0 | 0 | 0 | 0 | 0 | 0 | 0 | 0 | 0 | 0 | 0 | 4 | 0 |
| **in-pe** | 0 | 0 | 0 | 0 | 0 | 0 | 0 | 0 | 0 | 0 | 0 | 0 | 0 | 0 | 0 | 0 | 0 | 0 | 0 | 0 | 0 | 0 | 0 | 0 | 2 | 0 | 3 | 2 | 2 | 3 |
| **et-pe** | 0 | 0 | 0 | 0 | 0 | 0 | 0 | 0 | 0 | 0 | 0 | 0 | 0 | 0 | 0 | 0 | 0 | 0 | 0 | 0 | 0 | 0 | 0 | 0 | 0 | 0 | 2 | 0 | 2 | 4 |
| **fr-mx** | 0 | 0 | 0 | 0 | 0 | 0 | 0 | 0 | 0 | 0 | 0 | 0 | 0 | 0 | 0 | 0 | 0 | 0 | 0 | 0 | 0 | 0 | 0 | 0 | 0 | 0 | 0 | 0 | 0 | 0 |
| **fr-pl** | 0 | 0 | 0 | 0 | 0 | 0 | 0 | 0 | 0 | 0 | 0 | 0 | 0 | 0 | 0 | 0 | 0 | 0 | 0 | 0 | 0 | 0 | 0 | 0 | 0 | 2.5 | 2 | 0 | 0 | 4 |
| **as-fr** | 0 | 0 | 0 | 0 | 0 | 0 | 0 | 0 | 0 | 0 | 0 | 0 | 0 | 0 | 0 | 0 | 0 | 0 | 0 | 0 | 0 | 0 | 0 | 0 | 0 | 0 | 0 | 0 | 4 | 4 |
| **as-pa** | 0 | 0 | 0 | 0 | 0 | 0 | 0 | 0 | 0 | 0 | 0 | 0 | 0 | 0 | 0 | 0 | 0 | 0 | 0 | 0 | 0 | 0 | 0 | 0 | 0 | 0 | 0 | 0 | 0 | 0 |
| **bo-eo** | 0 | 0 | 0 | 0 | 0 | 0 | 0 | 0 | 0 | 0 | 0 | 0 | 0 | 0 | 0 | 0 | 0 | 0 | 0 | 0 | 0 | 0 | 0 | 0 | 0 | 0 | 0 | 0 | 0 | 4 |
| **ip-sq** | 0 | 0 | 0 | 0 | 0 | 0 | 0 | 0 | 0 | 0 | 0 | 0 | 0 | 0 | 0 | 0 | 0 | 0 | 0 | 0 | 0 | 0 | 0 | 0 | 0 | 0 | 0 | 0 | 2 | 0 |
| **so-eo** | 0 | 0 | 0 | 0 | 0 | 0 | 0 | 0 | 0 | 0 | 0 | 0 | 0 | 0 | 0 | 0 | 0 | 0 | 0 | 0 | 0 | 0 | 0 | 0 | 0 | 0 | 0 | 0 | 2 | 4 |
| **so-sq** | 0 | 0 | 0 | 0 | 0 | 0 | 0 | 0 | 0 | 0 | 0 | 0 | 0 | 0 | 0 | 0 | 0 | 0 | 0 | 0 | 0 | 0 | 0 | 0 | 0 | 0 | 0 | 0 | 2 | 4 |
| **so-pe** | 0 | 0 | 0 | 0 | 0 | 0 | 0 | 0 | 0 | 0 | 0 | 0 | 0 | 0 | 0 | 0 | 0 | 0 | 0 | 0 | 0 | 0 | 0 | 0 | 0 | 0 | 0 | 0 | 0 | 0 |
| **ju-de** | 0 | 0 | 0 | 0 | 0 | 0 | 0 | 0 | 0 | 0 | 0 | 0 | 0 | 0 | 0 | 0 | 0 | 0 | 0 | 0 | 0 | 0 | 0 | 0 | 0 | 0 | 0 | 0 | 0 | 0 |
| **as-de** | 0 | 0 | 0 | 0 | 0 | 0 | 0 | 0 | 0 | 0 | 0 | 0 | 0 | 0 | 0 | 0 | 0 | 0 | 0 | 0 | 0 | 0 | 0 | 0 | 0 | 0 | 0 | 0 | 0 | 0 |
| **sq-de** | 0 | 0 | 0 | 0 | 0 | 0 | 0 | 0 | 0 | 0 | 0 | 0 | 0 | 0 | 0 | 0 | 0 | 0 | 0 | 0 | 0 | 0 | 0 | 0 | 0 | 0 | 0 | 0 | 0 | 0 |

**Supplementary Table 12.** The data matrix for the onset of bone contact in *Dasyurus viverrinus*.

| **Genus** | *Dasyurus* | *Dasyurus* | *Dasyurus* | *Dasyurus* | *Dasyurus* | *Dasyurus* | *Dasyurus* | *Dasyurus* | *Dasyurus* | *Dasyurus* | *Dasyurus* | *Dasyurus* | *Dasyurus* | *Dasyurus* | *Dasyurus* |
| --- | --- | --- | --- | --- | --- | --- | --- | --- | --- | --- | --- | --- | --- | --- | --- |
| **Species** | *viverrinus* | *viverrinus* | *viverrinus* | *viverrinus* | *viverrinus* | *viverrinus* | *viverrinus* | *viverrinus* | *viverrinus* | *viverrinus* | *viverrinus* | *viverrinus* | *viverrinus* | *viverrinus* | *viverrinus* |
| **Specimen ID** | ZMB_EMB_MA759A | ZMB_EMB_MA750 | ZMB_EMB_MA752B | ZMB_EMB_MA718 | ZMB_EMB_MA758A | ZMB_EMB_MA759B | ZMB_EMB_MA758B | ZMB_EMB_MA735 | ZMB_EMB_MA752A | ZMB_EMB_MA726 | ZMB_EMB_MA716 | ZMB_EMB_MA717 | ZMB_EMB_MA712A | ZMB_EMB_MA712B | ZMB_Mam_72258 |
| **IW ID** | IW1530 | IW1531 | IW1532 | IW1535 | IW1534 | IW1536 | IW1537 | IW1533 | IW1538 | IW1539 | IW1541 | IW1540 | IW1542 | IW1543 | IW1544 |
| **Rank** | 1 | 2 | 3 | 4 | 5 | 6 | 7 |  | 8 | 9 | 10 | 11 | 12 | 13 | 14 |
| **so-ip** | 0 | 4 | 4 | 4 | 4 | 4 | 4 | 4 | 4 | 4 | 4 | 4 | 4 | 3.5 | 4 |
| **ip-pa** | 0 | 0 | 0 | 0 | 0 | 0 | 0 | 0 | 0 | 0 | 0 | 0 | 4 | 4 | 4 |
| **pa-fr** | 0 | 0 | 0 | 2 | 0 | 2 | 3 | 3 | 2.5 | 2 | 0 | 4 | 4 | 4 | 4 |
| **fr-na** | 0 | 0 | 2 | 0 | 0 | 0 | 2 | 2 | 2 | 0 | 2 | 2 | 3.5 | 4 | 4 |
| **sq-pa** | 0 | 0 | 0 | 0 | 0 | 0 | 0 | 0 | 0 | 0 | 0 | 4 | 4 | 4 | 4 |
| **as-sq** | 0 | 0 | 0 | 0 | 0 | 2 | 2 | 2 | 2 | 2 | 0 | 3 | 2.5 | 4 | 4 |
| **pt-bs** | 0 | 2 | 2 | 0 | 3.5 | 3.5 | 3.5 | 4 | 4 | 2 | 4 | 2 | 4 | 4 | 4 |
| **as-pt** | 0 | 0 | 0 | 2 | 0 | 0 | 0 | 0 | 0 | 2 | 3 | 2 | ? | 4 | 4 |
| **as-bs** | 0 | 0 | 2 | 0 | 0 | 0 | 0 | 0 | 0 | 0 | 3 | 0 | 4 | 4 | 4 |
| **pt-os** | 0 | 0 | 0 | 0 | 0 | 0 | 0 | 0 | 0 | 0 | 0 | 2 | ? | 4 | 4 |
| **as-os** | 0 | 0 | 0 | 2 | 0 | 2 | 2 | 0 | 2 | 2 | 0 | 0 | 2 | 4 | 4 |
| **bs-os** | 0 | 0 | 0 | 0 | 0 | 2 | 0 | 0 | 0 | 0 | 2 | 2 | 2 | 2 | 4 |
| **et-go** | 2 | 3 | 3 | 0 | 3.5 | 3.5 | 3.5 | 3.5 | 4 | 3 | 3 | 2 | 0 | 4 | 3.5 |
| **go-ml** | 4 | 4 | 4 | 4 | 4 | 4 | 4 | 4 | 4 | 4 | 4 | 4 | 4 | 4 | 4 |
| **go-pe** | 0 | 0 | 0 | 0 | 0 | 0 | 0 | 0 | 0 | 0 | 0 | 0 | 0 | 0 | 0 |
| **go-as** | 0 | 0 | 0 | 0 | 0 | 0 | 0 | 0 | 2 | 0 | 0 | 0 | 0 | 0 | 0 |
| **vo-pl** | 0 | 0 | 0 | 0 | 2 | 2 | 2 | 2 | 2 | 0 | 2 | 0 | 2 | 3 | 4 |
| **vo-os** | 0 | 0 | 0 | 0 | 0 | 0 | 0 | 0 | 0 | 0 | 0 | 0 | 0 | 4 | 4 |
| **vo-fr(na spt)** | 0 | 0 | 0 | 0 | 0 | 0 | 0 | 0 | 0 | 0 | 0 | 0 | 0 | 0 | 4 |
| **mx-pl** | 4 | 4 | 4 | 3 | 4 | 4 | 4 | 4 | 4 | 4 | 4 | 4 | 4 | 4 | 4 |
| **os-pl** | 0 | 0 | 0 | 0 | 0 | 0 | 0 | 0 | 0 | 0 | 0 | 0 | 0 | 2 | 2 |
| **mx-os** | 0 | 2 | 0 | 2 | 2 | 2.5 | 2 | 3 | 2.5 | 2 | 3 | 4 | ? | 4 | 4 |
| **os-fr** | 0 | 0 | 0 | 0 | 0 | 0 | 0 | 0 | 0 | 0 | 0 | 2 | 2 | 4 | 4 |
| **la-mx** | 0 | 0 | 0 | 0 | 0 | 0 | 2 | 3 | 0 | 2 | 0 | 2 | 0 | 4 | 4 |
| **la-pl** | 0 | 0 | 0 | 0 | 0 | 0 | 0 | 0 | 2 | 2 | 0 | 0 | 2 | 4 | 4 |
| **la-ju** | 0 | 0 | 0 | 0 | 0 | 0 | 0 | 0 | 0 | 0 | 0 | 0 | 2 | 4 | 4 |
| **mx-ju** | 0 | 2 | 2 | 3 | 2 | 3 | 2 | 3.5 | 2 | 4 | 3 | 4 | 3.5 | 4 | 4 |
| **ju-sq** | 0 | 0 | 0 | 0 | 0 | 0 | 2 | 0 | 0 | 2 | 0 | 0 | 0 | 3 | 4 |
| **et-de** | 0 | 0 | 0 | 0 | 0 | 0 | 0 | 0 | 0 | 0 | 0 | 0 | 0 | 0 | 0 |
| **pm-pl** | 0 | 0 | 0 | 0 | 0 | 0 | 0 | 0 | 0 | 0 | 0 | 0 | 0 | 4 | 4 |
| **pm-vo** | 0 | 0 | 0 | 0 | 0 | 0 | 0 | 0 | 0 | 0 | 0 | 0 | 0 | 4 | 4 |
| **pm-mx** | 0 | 0 | 0 | 0 | 2 | 0 | 0 | 2 | 0 | 0 | 0 | 2 | 2 | 3.5 | 4 |
| **pm-na** | 0 | 0 | 0 | 2 | 2 | 0 | 2 | 0 | 4 | 2 | 2 | 2 | 3.5 | 4 | 4 |
| **mx-na** | 0 | 0 | 0 | 2 | 2 | 0 | 0 | 0 | 2 | 3 | 2 | 3 | 4 | 4 | 4 |
| **fr-la** | 0 | 0 | 0 | 0 | 0 | 0 | 0 | 0 | 0 | 2 | 3 | 0 | 2 | 4 | 4 |
| **la-na** | 0 | 0 | 0 | 0 | 0 | 0 | 0 | 0 | 0 | 0 | 0 | 0 | 0 | 0 | 0 |
| **pe-eo** | 0 | 0 | 0 | 2 | 0 | 2.5 | 0 | 3 | 2 | 2 | 2 | 2 | 2 | 3.5 | 4 |
| **pe-bo** | 0 | 0 | 0 | 0 | 0 | 0 | 0 | 0 | 0 | 0 | 0 | 2 | 0 | 3.5 | 4 |
| **pe-sq** | 0 | 0 | 0 | 0 | 0 | 0 | 0 | 0 | 0 | 0 | 2 | 0 | 3 | 4 | 4 |
| **pe-pa** | 0 | 0 | 0 | 0 | 0 | 0 | 0 | 0 | 0 | 0 | 0 | 2 | 4 | 4 | 4 |
| **pe-as** | 0 | 0 | 0 | 0 | 0 | 0 | 0 | 0 | 0 | 0 | 0 | 2 | 0 | 4 | 4 |
| **pr-os** | 0 | 0 | 0 | 0 | 0 | 0 | 0 | 0 | 0 | 0 | 2 | 0 | 2 | 4 | 4 |
| **pr-fr** | 0 | 0 | 0 | 0 | 0 | 0 | 0 | 0 | 0 | 0 | 0 | 0 | 3 | 4 | 4 |
| **pr-vo** | 0 | 0 | 0 | 0 | 0 | 0 | 0 | 0 | 0 | 0 | 0 | 0 | 0 | 4 | 4 |
| **pr-as** | 0 | 0 | 0 | 0 | 0 | 0 | 0 | 0 | 0 | 0 | 0 | 0 | 0 | 2 | 4 |
| **pr-bs** | 0 | 0 | 0 | 0 | 0 | 0 | 0 | 0 | 0 | 0 | 0 | 0 | 0 | 0 | 4 |
| **in-ml** | 0 | 0 | 0 | 0 | 0 | 0 | 0 | 0 | 0 | 0 | 0 | 0 | 3 | 3 | 3 |
| **bo-bs** | 0 | 0 | 0 | 0 | 0 | 0 | 0 | 0 | 0 | 0 | 0 | 0 | 0 | 0 | 4 |
| **bs-pe** | 0 | 0 | 0 | 0 | 0 | 0 | 0 | 0 | 0 | 0 | 0 | 0 | 3 | 2 | 4 |
| **et-sq** | 0 | 0 | 0 | 0 | 0 | 0 | 0 | 0 | 0 | 0 | 0 | 0 | 0 | 0 | 0 |
| **as-et** | 0 | 0 | 0 | 0 | 0 | 0 | 0 | 0 | 0 | 2 | 2 | 0 | 0 | 0 | 0 |
| **in-pe** | 0 | 0 | 0 | 0 | 0 | 0 | 0 | 0 | 0 | 0 | 0 | 0 | 0 | 3 | 2 |
| **et-pe** | 0 | 0 | 0 | 0 | 0 | 0 | 0 | 0 | 0 | 0 | 0 | 2 | 2 | 3.5 | 2 |
| **fr-mx** | 0 | 0 | 0 | 0 | 0 | 0 | 0 | 0 | 0 | 0 | 0 | 0 | 2 | 4 | 4 |
| **fr-pl** | 0 | 0 | 0 | 0 | 0 | 0 | 0 | 0 | 0 | 0 | 0 | 2 | 2 | 4 | 4 |
| **as-fr** | 0 | 0 | 0 | 0 | 0 | 0 | 0 | 0 | 0 | 0 | 0 | 0 | 0 | 4 | 4 |
| **as-pa** | 0 | 0 | 0 | 0 | 0 | 0 | 0 | 0 | 0 | 0 | 0 | 0 | 0 | 0 | 0 |
| **bo-eo** | 0 | 0 | 0 | 0 | 0 | 0 | 0 | 0 | 0 | 0 | 0 | 0 | 2 | 3 | 4 |
| **ip-sq** | 0 | 0 | 0 | 0 | 0 | 0 | 0 | 0 | 0 | 0 | 0 | 0 | 0 | 0 | 0 |
| **so-eo** | 0 | 0 | 0 | 0 | 0 | 0 | 0 | 0 | 0 | 0 | 0 | 0 | 0 | 2.5 | 4 |
| **so-sq** | 0 | 0 | 0 | 0 | 0 | 0 | 0 | 0 | 0 | 0 | 0 | 0 | 0 | 0 | 0 |
| **so-pe** | 0 | 0 | 0 | 0 | 0 | 0 | 0 | 0 | 0 | 0 | 0 | 0 | 0 | 0 | 4 |
| **ju-de** | 0 | 0 | 0 | 0 | 0 | 0 | 0 | 0 | 0 | 0 | 0 | 0 | 0 | 2 | 0 |
| **as-de** | 0 | 0 | 0 | 0 | 0 | 0 | 0 | 0 | 0 | 0 | 0 | 0 | 0 | 0 | 0 |
| **sq-de** | 0 | 0 | 0 | 0 | 0 | 0 | 0 | 0 | 0 | 0 | 0 | 0 | 0 | 2 | 0 |

**Supplementary Table 13.** The data matrix for the onset of bone contact in *Phascolarctos cinereus*.

| **Genus** | *Phascolarctos* | *Phascolarctos* | *Phascolarctos* | *Phascolarctos* | *Phascolarctos* | *Phascolarctos* | *Phascolarctos* | *Phascolarctos* | *Phascolarctos* | *Phascolarctos* | *Phascolarctos* | *Phascolarctos* | *Phascolarctos* | *Phascolarctos* | *Phascolarctos* | *Phascolarctos* | *Phascolarctos* | *Phascolarctos* | *Phascolarctos* | *Phascolarctos* | *Phascolarctos* | *Phascolarctos* | *Phascolarctos* | *Phascolarctos* | *Phascolarctos* | *Phascolarctos* | *Phascolarctos* |
| --- | --- | --- | --- | --- | --- | --- | --- | --- | --- | --- | --- | --- | --- | --- | --- | --- | --- | --- | --- | --- | --- | --- | --- | --- | --- | --- | --- |
| **Species** | *cinereus* | *cinereus* | *cinereus* | *cinereus* | *cinereus* | *cinereus* | *cinereus* | *cinereus* | *cinereus* | *cinereus* | *cinereus* | *cinereus* | *cinereus* | *cinereus* | *cinereus* | *cinereus* | *cinereus* | *cinereus* | *cinereus* | *cinereus* | *cinereus* | *cinereus* | *cinereus* | *cinereus* | *cinereus* | *cinereus* | *cinereus* |
| **Specimen ID** | ZMB_EMB_MA487 2 | ZMB_EMB_MA487 1 | ZMB_EMB_MA488 | ZMB_EMB_MA485a | ZMB_EMB_MA486 | ZMB_EMB_MA503a 1 | ZMB_EMB_MA503a 2 | ZMB_EMB_MA499 2 | ZMB_EMB_MA500b | ZMB_EMB_MA497 2 | ZMB_EMB_MA499 1 | ZMB_EMB_MA502 | ZMB_EMB_MA497 1 | ZMB_EMB_MA484 | ZMB_EMB_MA498 2 | ZMB_EMB_MA498 1 | ZMB_EMB_MA496 | ZMB_EMB_MA483 | ZMB_EMB_MA495 2 | ZMB_EMB_MA494 2 | ZMB_EMB_MA495 1 | ZMB_EMB_MA500a | ZMB_EMB_MA491 | ZMB_EMB_MA492 | ZMB_EMB_MA506 | ZMB_EMB_MA507 | ZMB_Mam_36036 |
| **IW ID** | IW1545 | IW1546 | IW1547 | IW1550 | IW1549 | IW1552 | IW1553 | IW1548 | IW1554 | IW1551 | IW1555 | IW1560 | IW1556 | IW1557 | IW1559 | IW1561 | IW1562 | IW1566 | IW1563 | IW1565 | IW1564 | IW1558 | IW1567 | IW1568 | IW1569 | IW1570 | IW1571 |
| **Rank** |  |  |  |  | 1 | 2 |  | 3 | 4 | 5 | 6 |  | 7 | 8 | 9 |  | 10 | 11 |  | 12 | 13 | 14 | 15 | 16 | 17 | 18 | 19 |
| **so-ip** | 0 | 0 | 0 | 0 | 0 | 0 | 0 | 0 | 0 | 0 | 0 | 0 | 0 | 2 | 0 | 2 | 0 | 0 | 0 | 2 | 0 | 2 | 3 | 4 | 4 | 2.5 | 4 |
| **ip-pa** | 0 | 0 | 0 | 0 | 0 | 0 | 0 | 0 | 0 | 0 | 0 | 0 | 2 | 0 | 0 | 0 | 2 | 0 | 0 | 0 | 0 | 0 | 0 | 2 | 4 | 4 | 4 |
| **pa-fr** | 0 | 0 | 0 | 0 | 0 | 0 | 0 | 0 | 0 | 0 | 0 | 0 | 0 | 0 | 0 | 0 | 0 | 2 | 2 | 2.5 | 0 | 0 | 3 | 3 | 4 | 3.5 | 4 |
| **fr-na** | 0 | 0 | 0 | 0 | 0 | 0 | 0 | 0 | 0 | 0 | 0 | 0 | 0 | 0 | 0 | 0 | 0 | 2 | 2 | 0 | 0 | 2 | 2 | 2 | 4 | 3 | 4 |
| **sq-pa** | 0 | 0 | 0 | 0 | 0 | 0 | 0 | 0 | 0 | 0 | 0 | 0 | 0 | 0 | 0 | 0 | 0 | 0 | 0 | 0 | 0 | 0 | 2 | 4 | 3.5 | 4 | 4 |
| **as-sq** | 0 | 0 | 0 | 0 | 0 | 0 | 0 | 0 | 0 | 0 | 0 | 0 | 0 | 0 | 0 | 0 | 0 | 0 | 0 | 0 | 2 | 0 | 2 | 2 | 4 | 4 | 4 |
| **pt-bs** | 0 | 0 | 0 | 0 | 0 | 0 | 0 | 0 | 0 | 0 | 2 | 0 | 2 | 2 | 0 | 2 | 0 | 2 | 2 | 0 | 3 | 3 | 3.5 | 4 | 4 | 4 | 4 |
| **as-pt** | 0 | 0 | 0 | 0 | 0 | 0 | 0 | 0 | 0 | 0 | 0 | 0 | 0 | 0 | 0 | 0 | 0 | 0 | 0 | 0 | 0 | 0 | 0 | 4 | 4 | 4 | 4 |
| **as-bs** | 0 | 0 | 0 | 0 | 0 | 0 | 0 | 0 | 0 | 0 | 0 | 0 | 0 | 0 | 0 | 0 | 0 | 0 | 0 | 4 | 0 | 0 | 4 | 4 | 4 | 4 | 4 |
| **pt-os** | 0 | 0 | 0 | 0 | 0 | 0 | 0 | 0 | 0 | 0 | 0 | 0 | 0 | 0 | 0 | 0 | 0 | 0 | 0 | 0 | 0 | 0 | 2 | 2 | 2 | 4 | 4 |
| **as-os** | 0 | 0 | 0 | 0 | 0 | 0 | 0 | 0 | 0 | 0 | 0 | 0 | 0 | 0 | 0 | 0 | 0 | 0 | 0 | 0 | 0 | 0 | 2 | 0 | 2 | 4 | 4 |
| **bs-os** | 0 | 0 | 0 | 0 | 0 | 0 | 0 | 0 | 0 | 0 | 0 | 0 | 0 | 0 | 0 | 0 | 0 | 0 | 0 | 0 | 0 | 0 | 0 | 0 | 0 | 4 | 4 |
| **et-go** | 0 | 0 | 0 | 0 | 0 | 0 | 0 | 4 | 2 | 0 | 0 | 0 | 0 | 0 | 0 | 0 | 0 | 0 | 2 | 2 | 4 | 2 | 4 | 3.5 | 3.5 | 0 | 0 |
| **go-ml** | 0 | 0 | 0 | 0 | 0 | 0 | 0 | 0 | 0 | 4 | 0 | 4 | 4 | 4 | 4 | 4 | 4 | 4 | 4 | 4 | 4 | 4 | 4 | 4 | 4 | 0 | 0 |
| **go-pe** | 0 | 0 | 0 | 0 | 0 | 0 | 0 | 0 | 0 | 0 | 0 | 0 | 0 | 0 | 0 | 0 | 0 | 0 | 0 | 0 | 0 | 0 | 0 | 0 | 0 | 0 | 0 |
| **go-as** | 0 | 0 | 0 | 0 | 0 | 0 | 0 | 0 | 0 | 0 | 0 | 0 | 0 | 0 | 0 | 0 | 0 | 0 | 0 | 0 | 0 | 0 | 0 | 0 | 2 | 0 | 0 |
| **vo-pl** | 0 | 0 | 0 | 0 | 0 | 0 | 0 | 0 | 0 | 0 | 0 | 0 | 0 | 0 | 0 | 0 | 0 | 2.5 | 2 | 2.5 | 3.5 | 2 | 3.5 | 4 | 4 | 4 | 4 |
| **vo-os** | 0 | 0 | 0 | 0 | 0 | 0 | 0 | 0 | 0 | 0 | 0 | 0 | 0 | 0 | 0 | 0 | 0 | 0 | 0 | 0 | 2 | 0 | 2 | 2 | 4 | 4 | 4 |
| **vo-fr(na spt)** | 0 | 0 | 0 | 0 | 0 | 0 | 0 | 0 | 0 | 0 | 0 | 0 | 0 | 0 | 0 | 0 | 0 | 0 | 0 | 0 | 0 | 0 | 0 | 0 | 0 | 0 | 0 |
| **mx-pl** | 0 | 0 | 0 | 0 | 0 | 4 | 4 | 0 | 0 | 0 | 3 | 3 | 2 | 2 | 4 | 3.5 | 4 | 4 | 4 | 4 | 4 | 4 | 4 | 4 | 4 | 4 | 4 |
| **os-pl** | 0 | 0 | 0 | 0 | 0 | 0 | 0 | 0 | 0 | 0 | 0 | 0 | 0 | 0 | 0 | 0 | 0 | 0 | 0 | 0 | 0 | 0 | 2 | 2 | 2 | 3 | 4 |
| **mx-os** | 0 | 0 | 0 | 0 | 0 | 0 | 0 | 0 | 0 | 0 | 0 | 0 | 0 | 0 | 0 | 0 | 2 | 2 | 2 | 2.5 | 2 | 2 | 2 | 3.5 | 4 | 4 | 4 |
| **os-fr** | 0 | 0 | 0 | 0 | 0 | 0 | 0 | 0 | 0 | 0 | 0 | 0 | 0 | 0 | 0 | 0 | 0 | 0 | 0 | 0 | 0 | 0 | 2 | 0 | 4 | 4 | 4 |
| **la-mx** | 0 | 0 | 0 | 0 | 0 | 0 | 0 | 0 | 0 | 0 | 0 | 0 | 0 | 0 | 0 | 0 | 2 | 2 | 2 | 2 | 0 | 2.5 | 2 | 2.5 | 3.5 | 3.5 | 4 |
| **la-pl** | 0 | 0 | 0 | 0 | 0 | 0 | 0 | 0 | 0 | 0 | 0 | 0 | 0 | 0 | 0 | 0 | 0 | 0 | 0 | 0 | 0 | 2.5 | 0 | 2 | 4 | 2 | 4 |
| **la-ju** | 0 | 0 | 0 | 0 | 0 | 0 | 0 | 0 | 0 | 0 | 0 | 0 | 0 | 0 | 0 | 0 | 0 | 0 | 0 | 2 | 0 | 0 | 2 | 2 | 4 | 2.5 | 4 |
| **mx-ju** | 0 | 0 | 0 | 0 | 0 | 0 | 0 | 0 | 2 | 2 | 2 | 2 | 2 | 2 | 2 | 2 | 2 | 2 | 3 | 2 | 2.5 | 2 | 3 | 3 | 4 | 3 | 4 |
| **ju-sq** | 0 | 0 | 0 | 0 | 2 | 0 | 0 | 0 | 0 | 0 | 0 | 0 | 0 | 0 | 0 | 0 | 0 | 0 | 0 | 0 | 2 | 2 | 2 | 3 | 3 | 3.5 | 4 |
| **et-de** | 0 | 0 | 0 | 0 | 0 | 0 | 0 | 0 | 0 | 0 | 0 | 0 | 0 | 0 | 0 | 0 | 0 | 0 | 0 | 0 | 0 | 0 | 0 | 0 | 0 | 0 | 0 |
| **pm-pl** | 0 | 0 | 0 | 0 | 0 | 0 | 0 | 0 | 0 | 0 | 0 | 0 | 0 | 0 | 0 | 0 | 0 | 0 | 0 | 0 | 0 | 0 | 3.5 | 4 | 3 | 3 | 4 |
| **pm-vo** | 0 | 0 | 0 | 0 | 0 | 0 | 0 | 0 | 0 | 0 | 0 | 0 | 0 | 0 | 0 | 0 | 0 | 0 | 0 | 0 | 0 | 0 | 4 | 2.5 | 4 | 4 | 4 |
| **pm-mx** | 0 | 0 | 0 | 0 | 0 | 0 | 0 | 0 | 0 | 0 | 0 | 0 | 0 | 0 | 2 | 0 | 0 | 2 | 0 | 0 | 2 | 2 | 3 | 3.5 | 3.5 | 2 | 4 |
| **pm-na** | 0 | 0 | 0 | 0 | 0 | 0 | 0 | 0 | 0 | 0 | 0 | 0 | 0 | 0 | 0 | 0 | 0 | 0 | 0 | 0 | 0 | 0 | 4 | 4 | 4 | 3.5 | 4 |
| **mx-na** | 0 | 0 | 0 | 0 | 0 | 0 | 0 | 0 | 0 | 0 | 0 | 0 | 0 | 0 | 0 | 0 | 0 | 0 | 0 | 0 | 0 | 0 | 0 | 0 | 0 | 2 | 4 |
| **fr-la** | 0 | 0 | 0 | 0 | 0 | 0 | 0 | 0 | 0 | 0 | 0 | 0 | 0 | 0 | 0 | 0 | 0 | 0 | 0 | 0 | 0 | 2 | 2 | 3.5 | 4 | 4 | 4 |
| **la-na** | 0 | 0 | 0 | 0 | 0 | 0 | 0 | 0 | 0 | 0 | 0 | 0 | 0 | 0 | 0 | 0 | 0 | 0 | 0 | 0 | 0 | 0 | 0 | 0 | 0 | 0 | 0 |
| **pe-eo** | 0 | 0 | 0 | 0 | 0 | 0 | 0 | 0 | 0 | 0 | 0 | 0 | 0 | 0 | 0 | 0 | 0 | 0 | 0 | 0 | 0 | 0 | 2 | 2 | 2 | 0 | 2.5 |
| **pe-bo** | 0 | 0 | 0 | 0 | 0 | 0 | 0 | 0 | 0 | 0 | 0 | 0 | 0 | 0 | 0 | 0 | 0 | 0 | 0 | 0 | 0 | 0 | 2 | 0 | 2 | 0 | 2 |
| **pe-sq** | 0 | 0 | 0 | 0 | 0 | 0 | 0 | 0 | 0 | 0 | 0 | 0 | 0 | 0 | 0 | 0 | 0 | 0 | 0 | 0 | 0 | 0 | 2.5 | 3 | 3.5 | 3.5 | 4 |
| **pe-pa** | 0 | 0 | 0 | 0 | 0 | 0 | 0 | 0 | 0 | 0 | 0 | 0 | 0 | 0 | 0 | 0 | 0 | 0 | 0 | 0 | 0 | 0 | 0 | 0 | 0 | 3.5 | 4 |
| **pe-as** | 0 | 0 | 0 | 0 | 0 | 0 | 0 | 0 | 0 | 0 | 0 | 0 | 0 | 0 | 0 | 0 | 0 | 0 | 0 | 0 | 0 | 0 | 0 | 0 | 0 | 0 | 3 |
| **pr-os** | 0 | 0 | 0 | 0 | 0 | 0 | 0 | 0 | 0 | 0 | 0 | 0 | 0 | 0 | 0 | 0 | 0 | 0 | 0 | 0 | 0 | 0 | 3.5 | 4 | 4 | 2 | 3.5 |
| **pr-fr** | 0 | 0 | 0 | 0 | 0 | 0 | 0 | 0 | 0 | 0 | 0 | 0 | 0 | 0 | 0 | 0 | 0 | 0 | 0 | 0 | 0 | 0 | 0 | 3 | 4 | 3.5 | 4 |
| **pr-vo** | 0 | 0 | 0 | 0 | 0 | 0 | 0 | 0 | 0 | 0 | 0 | 0 | 0 | 0 | 0 | 0 | 0 | 0 | 0 | 0 | 0 | 0 | 0 | 0 | 2 | 3.5 | 4 |
| **pr-as** | 0 | 0 | 0 | 0 | 0 | 0 | 0 | 0 | 0 | 0 | 0 | 0 | 0 | 0 | 0 | 0 | 0 | 0 | 0 | 0 | 0 | 0 | 0 | 0 | 0 | 3.5 | 3.5 |
| **pr-bs** | 0 | 0 | 0 | 0 | 0 | 0 | 0 | 0 | 0 | 0 | 0 | 0 | 0 | 0 | 0 | 0 | 0 | 0 | 0 | 0 | 0 | 0 | 0 | 0 | 0 | 0 | 2 |
| **in-ml** | 0 | 0 | 0 | 0 | 0 | 0 | 0 | 0 | 0 | 0 | 0 | 0 | 0 | 0 | 0 | 0 | 0 | 0 | 0 | 0 | 0 | 0 | 3.5 | 3.5 | 4 | 4 | 4 |
| **bo-bs** | 0 | 0 | 0 | 0 | 0 | 0 | 0 | 0 | 0 | 0 | 0 | 0 | 0 | 0 | 0 | 0 | 0 | 0 | 0 | 0 | 0 | 0 | 0 | 0 | 0 | 2 | 3.5 |
| **bs-pe** | 0 | 0 | 0 | 0 | 0 | 0 | 0 | 0 | 0 | 0 | 0 | 0 | 0 | 0 | 0 | 0 | 0 | 0 | 0 | 0 | 0 | 0 | 0 | 0 | 0 | 0 | 0 |
| **et-sq** | 0 | 0 | 0 | 0 | 0 | 0 | 0 | 0 | 0 | 0 | 0 | 0 | 0 | 0 | 0 | 0 | 0 | 0 | 0 | 0 | 0 | 0 | 2 | 0 | 0 | 0 | 0 |
| **as-et** | 0 | 0 | 0 | 0 | 0 | 0 | 0 | 0 | 0 | 0 | 0 | 0 | 0 | 0 | 0 | 0 | 0 | 0 | 0 | 0 | 0 | 0 | 0 | 0 | 2 | 0 | 0 |
| **in-pe** | 0 | 0 | 0 | 0 | 0 | 0 | 0 | 0 | 0 | 0 | 0 | 0 | 0 | 0 | 0 | 0 | 0 | 0 | 0 | 0 | 0 | 0 | 0 | 2 | 2 | 2 | 2 |
| **et-pe** | 0 | 0 | 0 | 0 | 0 | 0 | 0 | 0 | 0 | 0 | 0 | 0 | 0 | 0 | 0 | 0 | 0 | 0 | 0 | 0 | 0 | 0 | 0 | 0 | 0 | 4 | 4 |
| **fr-mx** | 0 | 0 | 0 | 0 | 0 | 0 | 0 | 0 | 0 | 0 | 0 | 0 | 0 | 0 | 0 | 0 | 0 | 0 | 0 | 0 | 0 | 0 | 0 | 0 | 2 | 2 | 4 |
| **fr-pl** | 0 | 0 | 0 | 0 | 0 | 0 | 0 | 0 | 0 | 0 | 0 | 0 | 0 | 0 | 0 | 0 | 0 | 0 | 0 | 0 | 0 | 0 | 0 | 0 | 3 | 3 | 4 |
| **as-fr** | 0 | 0 | 0 | 0 | 0 | 0 | 0 | 0 | 0 | 0 | 0 | 0 | 0 | 0 | 0 | 0 | 0 | 0 | 0 | 0 | 0 | 0 | 0 | 0 | 0 | 3.5 | 4 |
| **as-pa** | 0 | 0 | 0 | 0 | 0 | 0 | 0 | 0 | 0 | 0 | 0 | 0 | 0 | 0 | 0 | 0 | 0 | 0 | 0 | 0 | 0 | 0 | 0 | 0 | 3.5 | 4 | 4 |
| **bo-eo** | 0 | 0 | 0 | 0 | 0 | 0 | 0 | 0 | 0 | 0 | 0 | 0 | 0 | 0 | 0 | 0 | 0 | 0 | 0 | 0 | 0 | 0 | 0 | 0 | 0 | 0 | 4 |
| **ip-sq** | 0 | 0 | 0 | 0 | 0 | 0 | 0 | 0 | 0 | 0 | 0 | 0 | 0 | 0 | 0 | 0 | 0 | 0 | 0 | 0 | 0 | 0 | 0 | 0 | 0 | 0 | 0 |
| **so-eo** | 0 | 0 | 0 | 0 | 0 | 0 | 0 | 0 | 0 | 0 | 0 | 0 | 0 | 0 | 0 | 0 | 0 | 0 | 0 | 0 | 0 | 0 | 0 | 0 | 0 | 0 | 4 |
| **so-sq** | 0 | 0 | 0 | 0 | 0 | 0 | 0 | 0 | 0 | 0 | 0 | 0 | 0 | 0 | 0 | 0 | 0 | 0 | 0 | 0 | 0 | 0 | 0 | 0 | 0 | 2 | 4 |
| **so-pe** | 0 | 0 | 0 | 0 | 0 | 0 | 0 | 0 | 0 | 0 | 0 | 0 | 0 | 0 | 0 | 0 | 0 | 0 | 0 | 0 | 0 | 0 | 0 | 0 | 0 | 0 | 4 |
| **ju-de** | 0 | 0 | 0 | 0 | 0 | 0 | 0 | 0 | 0 | 0 | 0 | 0 | 0 | 0 | 0 | 0 | 0 | 0 | 0 | 0 | 0 | 0 | 0 | 0 | 0 | 0 | 0 |
| **as-de** | 0 | 0 | 0 | 0 | 0 | 0 | 0 | 0 | 0 | 0 | 0 | 0 | 0 | 0 | 0 | 0 | 0 | 0 | 0 | 0 | 0 | 0 | 0 | 0 | 0 | 0 | 0 |
| **sq-de** | 0 | 0 | 0 | 0 | 0 | 0 | 0 | 0 | 0 | 0 | 0 | 0 | 0 | 0 | 0 | 0 | 0 | 0 | 0 | 0 | 0 | 0 | 0 | 0 | 0 | 0 | 0 |

**Supplementary Table 14.** The data matrix for the onset of bone contact in *Vombatus ursinus*.

| **Genus** | *Vombatus* | *Vombatus* | *Vombatus* | *Vombatus* | *Vombatus* | *Vombatus* | *Vombatus* | *Vombatus* | *Vombatus* | *Vombatus* | *Vombatus* | *Vombatus* |
| --- | --- | --- | --- | --- | --- | --- | --- | --- | --- | --- | --- | --- |
| **Species** | *ursinus* | *ursinus* | *ursinus* | *ursinus* | *ursinus* | *ursinus* | *ursinus* | *ursinus* | *ursinus* | *ursinus* | *ursinus* | *ursinus* |
| **Specimen ID** | ZMB_EMB_MA521a | ZMB_EMB_MA522 | ZMB_EMB_MA524 | ZMB_EMB_MA518 | ZMB_EMB_MA520 | ZMB_EMB_MA519 | ZMB_EMB_MA517 | ZMB_EMB_MA538 | ZMB_EMB_MA516 | ZMB_EMB_MA523 | ZMB_EMB_MA537 | ZMB_Mam_5702 |
| **IW ID** | IW1572 | IW1573 | IW1575 | IW1574 | IW1577 | IW1579 | IW1576 | IW1578 | IW1580 | IW1581 | IW1582 | IW1583 |
| **Rank** |  |  | 1 | 2 | 3 | 4 | 5 | 6 | 7 | 8 | 9 | 10 |
| **so-ip** | 0 | 0 | 0 | 0 | 4 | 0 | 4 | 0 | 0 | 0 | 0 | 4 |
| **ip-pa** | 0 | 0 | 0 | 0 | 0 | 0 | 0 | 0 | 0 | 0 | 2 | 4 |
| **pa-fr** | 0 | 0 | 0 | 0 | 0 | 2 | 0 | 0 | 2 | 2 | 3 | 4 |
| **fr-na** | 0 | 0 | 0 | 0 | 0 | 0 | 0 | 0 | 0 | 2 | 0 | 4 |
| **sq-pa** | 0 | 0 | 0 | 0 | 0 | 0 | 0 | 2 | 2 | 3 | 2.5 | 4 |
| **as-sq** | 0 | 0 | 0 | 0 | 0 | 0 | 0 | 0 | 2 | 4 | 4 | 4 |
| **pt-bs** | 0 | 0 | 0 | 0 | 0 | 2 | 4 | 3 | 2 | 0 | 0 | 4 |
| **as-pt** | 0 | 0 | 0 | 0 | 0 | 0 | 0 | 0 | 0 | 4 | 4 | 4 |
| **as-bs** | 0 | 0 | 0 | 0 | 0 | 0 | 0 | 0 | 0 | 4 | 4 | 4 |
| **pt-os** | 0 | 0 | 0 | 0 | 0 | 0 | 2 | 2 | 0 | 2 | 2.5 | 4 |
| **as-os** | 0 | 0 | 0 | 0 | 0 | 0 | 2 | 0 | 0 | 2 | 2.5 | 4 |
| **bs-os** | 0 | 0 | 0 | 0 | 0 | 0 | 0 | 0 | 0 | 2 | 2.5 | 4 |
| **et-go** | 0 | 0 | 0 | 0 | 3.5 | 2 | 3.5 | 3.5 | 3 | 4 | ? | 0 |
| **go-ml** | 0 | 0 | 4 | 0 | 4 | 4 | 4 | 4 | 4 | 4 | ? | 0 |
| **go-pe** | 0 | 0 | 0 | 0 | 0 | 0 | 0 | 0 | 0 | 0 | 0 | 0 |
| **go-as** | 0 | 0 | 0 | 0 | 0 | 0 | 0 | 0 | 0 | 0 | 0 | 0 |
| **vo-pl** | 0 | 0 | 0 | 0 | 2 | 2.5 | 2.5 | 3 | 3 | 2 | ? | 4 |
| **vo-os** | 0 | 0 | 0 | 0 | 0 | 0 | 0 | 0 | 2 | 2 | ? | 4 |
| **vo-fr(na spt)** | 0 | 0 | 0 | 0 | 0 | 0 | 0 | 0 | 0 | 0 | 0 | 4 |
| **mx-pl** | 0 | 0 | 3 | 4 | 3.5 | 4 | 4 | 4 | 4 | 4 | 4 | 4 |
| **os-pl** | 0 | 0 | 0 | 0 | 0 | 0 | 0 | 0 | 2 | 0 | ? | 4 |
| **mx-os** | 0 | 0 | 0 | 3 | 3 | 2.5 | 2.5 | 2.5 | 3 | 4 | ? | 4 |
| **os-fr** | 0 | 0 | 0 | 0 | 0 | 0 | 0 | 0 | 0 | 0 | ? | 4 |
| **la-mx** | 0 | 0 | 0 | 0 | 0 | 3.5 | 0 | 3.5 | 4 | 3.5 | 2 | 4 |
| **la-pl** | 0 | 0 | 0 | 0 | 0 | 0 | 0 | 0 | 0 | 0 | 0 | 4 |
| **la-ju** | 0 | 0 | 0 | 0 | 0 | 0 | 0 | 0 | 0 | 0 | 2 | 4 |
| **mx-ju** | 0 | 0 | 0 | 0 | 3.5 | 0 | 2 | 2 | 2 | 3.5 | 4 | 4 |
| **ju-sq** | 0 | 0 | 0 | 0 | 0 | 0 | 0 | 0 | 0 | 2 | 0 | 4 |
| **et-de** | 0 | 0 | 0 | 0 | 0 | 0 | 0 | 0 | 0 | 0 | 0 | 0 |
| **pm-pl** | 0 | 0 | 0 | 0 | 0 | 0 | 0 | 2 | 0 | 2 | 4 | 4 |
| **pm-vo** | 0 | 0 | 0 | 0 | 0 | 0 | 0 | 0 | 0 | 2.5 | 3.5 | 4 |
| **pm-mx** | 0 | 0 | 0 | 0 | 2 | 2 | 3 | 2 | 3 | 3 | 2.5 | 4 |
| **pm-na** | 0 | 0 | 0 | 0 | 2 | 3 | 0 | 2 | 2 | 3 | 3.5 | 4 |
| **mx-na** | 0 | 0 | 0 | 0 | 0 | 0 | 0 | 0 | 2 | 0 | 0 | 4 |
| **fr-la** | 0 | 0 | 0 | 0 | 0 | 0 | 0 | 0 | 0 | 0 | 2 | 4 |
| **la-na** | 0 | 0 | 0 | 0 | 0 | 0 | 0 | 0 | 0 | 0 | 0 | 0 |
| **pe-eo** | 0 | 0 | 0 | 0 | 0 | 0 | 0 | 0 | 0 | 2.5 | 2 | 4 |
| **pe-bo** | 0 | 0 | 0 | 0 | 0 | 0 | 0 | 0 | 0 | 2 | 2 | 4 |
| **pe-sq** | 0 | 0 | 0 | 0 | 0 | 0 | 0 | 0 | 0 | 0 | 4 | 4 |
| **pe-pa** | 0 | 0 | 0 | 0 | 0 | 0 | 0 | 0 | 0 | 0 | 0 | 0 |
| **pe-as** | 0 | 0 | 0 | 0 | 0 | 0 | 0 | 0 | 0 | 0 | 0 | 4 |
| **pr-os** | 0 | 0 | 0 | 0 | 0 | 0 | 0 | 0 | 0 | 2 | 0 | 4 |
| **pr-fr** | 0 | 0 | 0 | 0 | 0 | 0 | 0 | 0 | 0 | 0 | 2 | 4 |
| **pr-vo** | 0 | 0 | 0 | 0 | 0 | 0 | 0 | 0 | 0 | 0 | ? | 4 |
| **pr-as** | 0 | 0 | 0 | 0 | 0 | 0 | 0 | 0 | 0 | 0 | 0 | 4 |
| **pr-bs** | 0 | 0 | 0 | 0 | 0 | 0 | 0 | 0 | 0 | 0 | 0 | 4 |
| **in-ml** | 0 | 0 | 0 | 0 | 0 | 0 | 0 | 0 | 0 | 0 | 2 | 4 |
| **bo-bs** | 0 | 0 | 0 | 0 | 0 | 0 | 0 | 0 | 0 | 0 | 0 | 4 |
| **bs-pe** | 0 | 0 | 0 | 0 | 0 | 0 | 0 | 0 | 0 | 0 | 0 | 0 |
| **et-sq** | 0 | 0 | 0 | 0 | 0 | 0 | 0 | 0 | 0 | 0 | 0 | 0 |
| **as-et** | 0 | 0 | 0 | 0 | 0 | 0 | 0 | 0 | 0 | 0 | 0 | 0 |
| **in-pe** | 0 | 0 | 0 | 0 | 0 | 0 | 0 | 0 | 0 | 0 | 2 | 2 |
| **et-pe** | 0 | 0 | 0 | 0 | 0 | 0 | 0 | 0 | 0 | 0 | 0 | 4 |
| **fr-mx** | 0 | 0 | 0 | 0 | 0 | 0 | 0 | 0 | 0 | 0 | 0 | 0 |
| **fr-pl** | 0 | 0 | 0 | 0 | 0 | 0 | 0 | 0 | 0 | 0 | ? | 4 |
| **as-fr** | 0 | 0 | 0 | 0 | 0 | 0 | 0 | 0 | 0 | 0 | 2 | 4 |
| **as-pa** | 0 | 0 | 0 | 0 | 0 | 0 | 0 | 0 | 0 | 0 | 0 | 4 |
| **bo-eo** | 0 | 0 | 0 | 0 | 0 | 0 | 0 | 0 | 0 | 0 | 0 | 4 |
| **ip-sq** | 0 | 0 | 0 | 0 | 0 | 0 | 0 | 0 | 0 | 0 | 0 | 0 |
| **so-eo** | 0 | 0 | 0 | 0 | 0 | 0 | 0 | 0 | 0 | 0 | 0 | 4 |
| **so-sq** | 0 | 0 | 0 | 0 | 0 | 0 | 0 | 0 | 0 | 0 | 0 | 4 |
| **so-pe** | 0 | 0 | 0 | 0 | 0 | 0 | 0 | 0 | 0 | 0 | 0 | 4 |
| **ju-de** | 0 | 0 | 0 | 0 | 0 | 0 | 0 | 0 | 0 | 0 | 0 | 0 |
| **as-de** | 0 | 0 | 0 | 0 | 0 | 0 | 0 | 0 | 0 | 0 | 0 | 0 |
| **sq-de** | 0 | 0 | 0 | 0 | 0 | 0 | 0 | 0 | 0 | 0 | 0 | 0 |

**Supplementary Table 15.** The data matrix for the onset of bone contact in *Petrogale penicillata*.

| **Genus** | *Petrogale* | *Petrogale* | *Petrogale* | *Petrogale* | *Petrogale* | *Petrogale* | *Petrogale* | *Petrogale* | *Petrogale* | *Petrogale* | *Petrogale* | *Petrogale* | *Petrogale* | *Petrogale* | *Petrogale* | *Petrogale* | *Petrogale* |
| --- | --- | --- | --- | --- | --- | --- | --- | --- | --- | --- | --- | --- | --- | --- | --- | --- | --- |
| **Species** | *penicillata* | *penicillata* | *penicillata* | *penicillata* | *penicillata* | *penicillata* | *penicillata* | *penicillata* | *penicillata* | *penicillata* | *penicillata* | *penicillata* | *penicillata* | *penicillata* | *penicillata* | *penicillata* | *penicillata* |
| **Specimen ID** | ZMB_EMB_MA583 | ZMB_EMB_MA587 | ZMB_EMB_MA577 | ZMB_EMB_MA589 2 | ZMB_EMB_MA590 | ZMB_EMB_MA564 | ZMB_EMB_MA588 1 | ZMB_EMB_MA588 3 | ZMB_EMB_MA576 | ZMB_EMB_MA589 1 | ZMB_EMB_MA588 2 | ZMB_EMB_MA591 | ZMB_EMB_MA585 | ZMB_EMB_MA593b | ZMB_EMB_MA592 | ZMB_EMB_MA593a | ZMB_Mam_4212 |
| **IW ID** | IW1585 | IW1584 | IW1587 | IW1588 | IW1592 | IW1593 | IW1586 | IW1589 | IW1594 | IW1590 | IW1591 | IW1596 | IW1595 | IW1598 | IW1597 | IW1599 | IW1600 |
| **Rank** | 1 | 2 | 3 | 4 | 5 |  | 6 | 7 | 8 | 9 | 10 | 11 | 12 | 13 | 14 | 15 | 16 |
| **so-ip** | 0 | 0 | 0 | 0 | 0 | 0 | 0 | 0 | 0 | 0 | 2 | 2 | 2 | 0 | 2 | 2 | 4 |
| **ip-pa** | 0 | 0 | 0 | 0 | 0 | 0 | 0 | 0 | 0 | 2 | 2 | 2 | 2 | 2 | 2 | 3 | 4 |
| **pa-fr** | 0 | 0 | 0 | 0 | 0 | 0 | 0 | 0 | 0 | 0 | 0 | 2 | 0 | 3 | 3 | 3 | 4 |
| **fr-na** | 0 | 0 | 0 | 0 | 0 | 0 | 0 | 0 | 2 | 0 | 0 | ? | 2 | 2 | 3 | 2.5 | 4 |
| **sq-pa** | 0 | 0 | 0 | 2 | 0 | 2 | 0 | 0 | 2 | 0 | 0 | 3.5 | 2 | 3.5 | 3 | 3.5 | 4 |
| **as-sq** | 0 | 0 | 0 | 0 | 0 | 0 | 0 | 0 | 2 | 0 | 0 | 2 | 2 | 3 | 3 | 4 | 4 |
| **pt-bs** | 0 | 0 | 0 | 0 | 2 | 2 | 2.5 | 2 | 0 | 3 | 4 | 0 | 2 | 2 | 2 | 3.5 | 4 |
| **as-pt** | 0 | 0 | 0 | 0 | 0 | 0 | 0 | 0 | 0 | 0 | 0 | 0 | 2 | 2.5 | 2 | 3.5 | 4 |
| **as-bs** | 0 | 0 | 0 | 0 | 0 | 0 | 0 | 0 | 0 | 0 | 0 | 0 | 0 | 0 | 0 | 0 | 4 |
| **pt-os** | 0 | 0 | 0 | 0 | 0 | 0 | 0 | 0 | 0 | 0 | 0 | 2 | 3 | 0 | 2 | 2 | 4 |
| **as-os** | 0 | 0 | 0 | 0 | 0 | 0 | 0 | 0 | 0 | 0 | 0 | 2 | 2 | 0 | 0 | 0 | 4 |
| **bs-os** | 0 | 0 | 0 | 0 | 0 | 0 | 0 | 0 | 0 | 0 | 0 | 0 | 0 | 0 | 0 | 0 | 4 |
| **et-go** | 0 | 0 | 4 | 4 | 4 | 4 | 4 | 4 | 4 | 4 | 4 | 4 | 4 | 4 | 4 | 4 | 0 |
| **go-ml** | 0 | 0 | 0 | 0 | 4 | 4 | 0 | 0 | 4 | 4 | 0 | 4 | 4 | 4 | 4 | 4 | 0 |
| **go-pe** | 0 | 0 | 0 | 0 | 0 | 0 | 0 | 0 | 0 | 0 | 0 | 0 | 0 | 0 | 0 | 0 | 0 |
| **go-as** | 0 | 0 | 0 | 0 | 0 | 0 | 0 | 0 | 0 | 0 | 0 | 0 | 0 | 0 | 0 | 0 | 0 |
| **vo-pl** | 0 | 0 | 0 | 3 | 3 | 3.5 | 3 | 2 | 3.5 | 3.5 | 3.5 | 0 | 2 | 2.5 | 4 | 2 | 4 |
| **vo-os** | 0 | 0 | 0 | 0 | 0 | 0 | 0 | 0 | 0 | 0 | 0 | 0 | 2 | 0 | 0 | 0 | 4 |
| **vo-fr(na spt)** | 0 | 0 | 0 | 0 | 0 | 0 | 0 | 0 | 0 | 0 | 0 | 0 | 0 | 0 | 0 | 0 | 4 |
| **mx-pl** | 4 | 4 | 4 | 4 | 4 | 4 | 4 | 4 | 4 | 4 | 4 | 0 | 4 | 4 | 4 | 4 | 4 |
| **os-pl** | 0 | 0 | 0 | 0 | 0 | 0 | 2 | 2 | 0 | 0 | 2 | 0 | 3 | 0 | 2 | 0 | 4 |
| **mx-os** | 0 | 2 | 2 | 2 | 2 | 2 | 2 | 4 | 2 | 3.5 | 3.5 | 0 | 2 | 2 | 4 | 4 | 4 |
| **os-fr** | 0 | 0 | 0 | 0 | 0 | 0 | 0 | 0 | 2 | 0 | 0 | 2 | 3 | 0 | 2 | 0 | 4 |
| **la-mx** | 0 | 0 | 2 | 2.5 | 2 | 3 | 3 | 2 | 2 | 3 | 2 | 3.5 | 2 | 3.5 | 3.5 | 2.5 | 4 |
| **la-pl** | 0 | 0 | 0 | 0 | 0 | 0 | 0 | 0 | 0 | 0 | 0 | 0 | 2 | 2 | 2 | 2 | 4 |
| **la-ju** | 0 | 0 | 0 | 2 | 0 | 0 | 0 | 0 | 0 | 0 | 2-4 | 2 | 0 | 0 | 0 | 0 | 4 |
| **mx-ju** | 0 | 2 | 2 | 3 | 3 | 2 | 2 | 3.5 | 3 | 3 | 3 | 3.5 | 2 | 3 | 4 | 3 | 4 |
| **ju-sq** | 0 | 2 | 0 | 2 | 2 | 2 | 3.5 | 3.5 | 0 | 2 | 2 | 2 | 0 | 2 | 3.5 | 2.5 | 4 |
| **et-de** | 0 | 0 | 0 | 0 | 0 | 0 | 2 | 2 | 0 | 0 | 2 | 0 | 0 | 0 | 0 | 0 | 0 |
| **pm-pl** | 0 | 0 | 0 | 2 | 0 | 0 | 2 | 0 | 0 | 0 | 0 | ? | 3 | 4 | 2 | 4 | 4 |
| **pm-vo** | 0 | 0 | 0 | 0 | 2 | 3 | 0 | 0 | 0 | 0 | 0 | ? | 2 | 4 | 4 | 4 | 4 |
| **pm-mx** | 0 | 0 | 2 | 2.5 | 2 | 0 | 3 | 2 | 0 | 3 | 2.5 | ? | 2.5 | 2 | 2.5 | 0 | 4 |
| **pm-na** | 0 | 0 | 0 | 0 | 0 | 0 | 0 | 0 | 3 | 0 | 0 | ? | 4 | 4 | 4 | 3 | 4 |
| **mx-na** | 0 | 0 | 0 | 0 | 0 | 0 | 0 | 0 | 0 | 0 | 0 | ? | 2.5 | 2 | 2 | 3 | 4 |
| **fr-la** | 0 | 0 | 0 | 0 | 0 | 0 | 0 | 2 | 0 | 0 | 0 | 3.5 | 2 | 2.5 | 2 | 3.5 | 4 |
| **la-na** | 0 | 0 | 0 | 0 | 0 | 0 | 0 | 0 | 0 | 0 | 0 | 0 | 0 | 0 | 0 | 0 | 0 |
| **pe-eo** | 0 | 0 | 0 | 0 | 0 | 0 | 0 | 0 | 0 | 0 | 0 | 2 | ? | 3 | 2 | 2.5 | 4 |
| **pe-bo** | 0 | 0 | 0 | 0 | 0 | 0 | 0 | 0 | 0 | 0 | 0 | 0 | ? | 2 | 0 | 0 | 4 |
| **pe-sq** | 0 | 0 | 0 | 0 | 0 | 0 | 0 | 0 | 0 | 0 | 0 | 0 | 0 | 0 | 0 | 2 | 4 |
| **pe-pa** | 0 | 0 | 0 | 0 | 0 | 0 | 0 | 0 | 0 | 0 | 0 | 0 | 0 | 0 | 0 | 0 | 0 |
| **pe-as** | 0 | 0 | 0 | 0 | 0 | 0 | 0 | 0 | 0 | 0 | 0 | 0 | 0 | 0 | 0 | 0 | 4 |
| **pr-os** | 0 | 0 | 0 | 0 | 0 | 0 | 0 | 0 | 0 | 0 | 0 | 0 | 0 | 0 | 0 | 2 | 4 |
| **pr-fr** | 0 | 0 | 0 | 0 | 0 | 0 | 0 | 0 | 0 | 0 | 0 | 0 | 0 | 0 | 0 | 0 | 4 |
| **pr-vo** | 0 | 0 | 0 | 0 | 0 | 0 | 0 | 0 | 0 | 0 | 0 | 0 | 0 | 0 | 0 | 0 | 4 |
| **pr-as** | 0 | 0 | 0 | 0 | 0 | 0 | 0 | 0 | 0 | 0 | 0 | 0 | 0 | 0 | 0 | 0 | 4 |
| **pr-bs** | 0 | 0 | 0 | 0 | 0 | 0 | 0 | 0 | 0 | 0 | 0 | 0 | 0 | 0 | 0 | 0 | 4 |
| **in-ml** | 0 | 0 | 0 | 0 | 0 | 0 | 0 | 0 | 0 | 0 | 0 | 0 | 0 | 3 | 3 | 3.5 | 4 |
| **bo-bs** | 0 | 0 | 0 | 0 | 0 | 0 | 0 | 0 | 0 | 0 | 0 | 0 | ? | 0 | 0 | 0 | 4 |
| **bs-pe** | 0 | 0 | 0 | 0 | 0 | 0 | 0 | 0 | 0 | 0 | 0 | 0 | ? | 0 | 0 | 0 | 0 |
| **et-sq** | 0 | 0 | 0 | 0 | 0 | 0 | 0 | 0 | 0 | 0 | 0 | 0 | 0 | 0 | 0 | 2 | 0 |
| **as-et** | 0 | 0 | 0 | 0 | 0 | 0 | 0 | 0 | 0 | 0 | 0 | 0 | 0 | 0 | 0 | 0 | 0 |
| **in-pe** | 0 | 0 | 0 | 0 | 0 | 0 | 0 | 0 | 0 | 0 | 0 | 0 | 0 | 0 | 2 | 2 | 2 |
| **et-pe** | 0 | 0 | 0 | 0 | 0 | 0 | 0 | 0 | 0 | 0 | 0 | 2 | 0 | 0 | 0 | 2 | 4 |
| **fr-mx** | 0 | 0 | 0 | 0 | 0 | 0 | 0 | 0 | 0 | 0 | 0 | ? | 0 | 2 | 2 | 3 | 4 |
| **fr-pl** | 0 | 0 | 0 | 0 | 0 | 0 | 0 | 0 | 0 | 0 | 0 | 0 | 0 | 0 | 0 | 0 | 4 |
| **as-fr** | 0 | 0 | 0 | 0 | 0 | 0 | 0 | 0 | 0 | 0 | 0 | 0 | 0 | 0 | 0 | 0 | 4 |
| **as-pa** | 0 | 0 | 0 | 0 | 0 | 0 | 0 | 0 | 0 | 0 | 0 | 0 | 0 | 0 | 0 | 0 | 4 |
| **bo-eo** | 0 | 0 | 0 | 0 | 0 | 0 | 0 | 0 | 0 | 0 | 0 | 0 | ? | 0 | 0 | 0 | 4 |
| **ip-sq** | 0 | 0 | 0 | 0 | 0 | 0 | 0 | 0 | 0 | 0 | 0 | 0 | 0 | 0 | 0 | 0 | 0 |
| **so-eo** | 0 | 0 | 0 | 0 | 0 | 0 | 0 | 0 | 0 | 0 | 0 | 0 | 0 | 0 | 0 | 0 | 4 |
| **so-sq** | 0 | 0 | 0 | 0 | 0 | 0 | 0 | 0 | 0 | 0 | 0 | 0 | 0 | 0 | 0 | 0 | 4 |
| **so-pe** | 0 | 0 | 0 | 0 | 0 | 0 | 0 | 0 | 0 | 0 | 0 | 0 | 0 | 0 | 0 | 0 | 4 |
| **ju-de** | 0 | 0 | 0 | 0 | 0 | 0 | 0 | 0 | 0 | 0 | 0 | 0 | 0 | 0 | 0 | 0 | 0 |
| **as-de** | 0 | 0 | 0 | 0 | 0 | 0 | 0 | 0 | 0 | 0 | 0 | 0 | 0 | 0 | 0 | 0 | 0 |
| **sq-de** | 0 | 0 | 0 | 0 | 0 | 0 | 0 | 0 | 0 | 0 | 0 | 0 | 0 | 0 | 0 | 0 | 0 |

**Supplementary Table 16.** Part one of the table showing the ranking of the timing of all of the individual bone contacts.

| **Ranking** | **so-ip** | **ip-pa** | **pa-fr** | **fr-na** | **sq-pa** | **as-sq** | **pt-bs** | **as-pt** | **as-bs** | **pt-os** | **as-os** | **bs-os** | **et-go** | **go-ml** | **go-pe** | **go-as** | **vo-pl** | **vo-os** | **vo-fr** | **mx-pl** | **os-pl** |
| --- | --- | --- | --- | --- | --- | --- | --- | --- | --- | --- | --- | --- | --- | --- | --- | --- | --- | --- | --- | --- | --- |
| *T. vulpecula* | 1 | 9 | 7 | 12 | 13 | 14 | 3 | 6 | 6 | 11 | 15 | 20 | 4 | 2 | - | - | 8 | 20 | 20 | 3 | 21 |
| *D. viverrinus* | 2 | 12 | 4 | 3 | 11 | 6 | 2 | 4 | 3 | 11 | 4 | 6 | 1 | 1 | - | 8 | 5 | 13 | 14 | 1 | 13 |
| *Ph. cinereus* | 8 | 7 | 11 | 11 | 15 | 13 | 6 | 16 | 12 | 15 | 15 | 18 | 3 | 5 | - | 17 | 11 | 13 | - | 2 | 15 |
| *V. ursinus* | 3 | 9 | 4 | 8 | 6 | 7 | 4 | 8 | 8 | 5 | 5 | 8 | 3 | 1 | - | - | 3 | 7 | 10 | 1 | 7 |
| *Pe. penicillata* | 10 | 9 | 11 | 8 | 4 | 8 | 5 | 12 | 16 | 11 | 11 | 16 | 3 | 5 | - | - | 4 | 12 | 16 | 1 | 6 |

**Supplementary Table 17.** Part two of the table showing the ranking of the timing of all of the individual bone contacts.

| **Ranking** | **mx-os** | **os-fr** | **la-mx** | **la-pl** | **la-ju** | **mx-ju** | **ju-sq** | **et-de** | **pm-pl** | **pm-vo** | **pm-mx** | **pm-na** | **mx-na** | **fr-la** | **la-na** | **pe-eo** | **pe-bo** | **pe-sq** | **pe-pa** | **pe-as** | **pr-os** | **pr-fr** |
| --- | --- | --- | --- | --- | --- | --- | --- | --- | --- | --- | --- | --- | --- | --- | --- | --- | --- | --- | --- | --- | --- | --- |
| *T. vulpecula* | 2 | 14 | 12 | 10 | 12 | 5 | 9 | 11 | 16 | 21 | 12 | 7 | 14 | 13 | 20 | 17 | 17 | 16 | - | 20 | 16 | 16 |
| *D. viverrinus* | 2 | 11 | 7 | 8 | 12 | 2 | 7 | - | 13 | 13 | 5 | 4 | 4 | 9 | - | 4 | 11 | 10 | 11 | 11 | 10 | 12 |
| *Ph. cinereus* | 10 | 15 | 10 | 14 | 12 | 4 | 1 | - | 15 | 15 | 9 | 15 | 18 | 14 | - | 15 | 15 | 15 | 18 | 19 | 15 | 16 |
| *V. ursinus* | 2 | 10 | 4 | 10 | 9 | 3 | 8 | - | 6 | 8 | 3 | 3 | 7 | 9 | - | 8 | 8 | 9 | - | 10 | 8 | 9 |
| *Pe. penicillata* | 2 | 8 | 3 | 12 | 4 | 2 | 2 | 6 | 4 | 5 | 3 | 8 | 12 | 7 | - | 11 | 13 | 15 | - | 16 | 15 | 16 |

**Supplementary Table 18.** Part three of the table showing the ranking of the timing of all of the individual bone contacts.

| **Ranking** | **pr-vo** | **pr-as** | **pr-bs** | **in-ml** | **bo-bs** | **bs-pe** | **et-sq** | **as-et** | **in-pe** | **et-pe** | **fr-mx** | **fr-pl** | **as-fr** | **as-pa** | **bo-eo** | **ip-sq** | **so-eo** | **so-sq** | **so-pe** | **ju-de** | **as-de** | **sq-de** |
| --- | --- | --- | --- | --- | --- | --- | --- | --- | --- | --- | --- | --- | --- | --- | --- | --- | --- | --- | --- | --- | --- | --- |
| *T. vulpecula* | 16 | 21 | 21 | 16 | 21 | - | 19 | 20 | 16 | 18 | - | 17 | 20 | - | 21 | 20 | 20 | 20 | - | - | - | - |
| *D. viverrinus* | 13 | 13 | 14 | 12 | 14 | 12 | - | 9 | 13 | 11 | 12 | 11 | 13 | - | 12 | - | 13 | - | 14 | 13 | - | 13 |
| *Ph. cinereus* | 17 | 18 | 19 | 15 | 18 | - | 15 | 17 | 16 | 18 | 17 | 17 | 18 | 17 | 19 | - | 19 | 18 | 19 | - | - | - |
| *V. ursinus* | 10 | 10 | 10 | 9 | 10 | - | - | - | 9 | - | 10 | 10 |  | - | 10 | - | 10 | - | - | - | - | - |
| *Pe. penicillata* | 16 | 16 | 16 | 13 | 16 | - | 15 | - | 14 | 11 | 13 | 16 |  | - | 16 | - | 16 | - | - | - | - | - |

**Supplementary Table 19.** Part one of the table showing the relative timing of all of the bone contacts.

| **Relative timing** | **so-ip** | **ip-pa** | **pa-fr** | **fr-na** | **sq-pa** | **as-sq** | **pt-bs** | **as-pt** | **as-bs** | **pt-os** | **as-os** | **bs-os** | **et-go** | **go-ml** | **go-pe** | **go-as** | **vo-pl** | **vo-os** | **vo-fr** | **mx-pl** | **os-pl** |
| --- | --- | --- | --- | --- | --- | --- | --- | --- | --- | --- | --- | --- | --- | --- | --- | --- | --- | --- | --- | --- | --- |
| *T. vulpecula* | 0.05 | 0.43 | 0.33 | 0.57 | 0.62 | 0.67 | 0.14 | 0.29 | 0.29 | 0.52 | 0.71 | 0.95 | 0.19 | 0.10 | - | - | 0.38 | 0.95 | 0.95 | 0.14 | 1.00 |
| *D. viverrinus* | 0.14 | 0.86 | 0.29 | 0.21 | 0.79 | 0.43 | 0.14 | 0.29 | 0.21 | 0.79 | 0.29 | 0.43 | 0.07 | 0.07 | - | 0.57 | 0.36 | 0.93 | 1.00 | 0.07 | 0.93 |
| *Ph. cinereus* | 0.42 | 0.37 | 0.58 | 0.58 | 0.79 | 0.68 | 0.32 | 0.84 | 0.63 | 0.79 | 0.79 | 0.95 | 0.16 | 0.26 | - | 0.89 | 0.58 | 0.68 | - | 0.11 | 0.79 |
| *V. ursinus* | 0.30 | 0.90 | 0.40 | 0.80 | 0.60 | 0.70 | 0.40 | 0.80 | 0.80 | 0.50 | 0.50 | 0.80 | 0.30 | 0.10 | - | - | 0.30 | 0.70 | 1.00 | 0.10 | 0.70 |
| *Pe. penicillata* | 0.63 | 0.56 | 0.69 | 0.50 | 0.25 | 0.50 | 0.31 | 0.75 | 1.00 | 0.69 | 0.69 | 1.00 | 0.19 | 0.31 | - | - | 0.25 | 0.75 | 1.00 | 0.06 | 0.38 |

**Supplementary Table 20.** Part two of the table showing the relative timing of all of the bone contacts.

| **Relative timing** | **mx-os** | **os-fr** | **la-mx** | **la-pl** | **la-ju** | **mx-ju** | **ju-sq** | **et-de** | **pm-pl** | **pm-vo** | **pm-mx** | **pm-na** | **mx-na** | **fr-la** | **la-na** | **pe-eo** | **pe-bo** | **pe-sq** | **pe-pa** | **pe-as** | **pr-os** | **pr-fr** |
| --- | --- | --- | --- | --- | --- | --- | --- | --- | --- | --- | --- | --- | --- | --- | --- | --- | --- | --- | --- | --- | --- | --- |
| *T. vulpecula* | 0.00 | 0.67 | 0.57 | 0.48 | 0.57 | 0.24 | 0.43 | - | 0.76 | 1.00 | 0.57 | 0.33 | 0.67 | 0.62 | 0.95 | 0.81 | 0.81 | 0.76 | - | 0.95 | 0.76 | 0.76 |
| *D. viverrinus* | 0.14 | 0.79 | 0.50 | 0.57 | 0.86 | 0.14 | 0.50 | - | 0.93 | 0.93 | 0.36 | 0.29 | 0.29 | 0.64 | - | 0.29 | 0.79 | 0.71 | 0.79 | 0.79 | 0.71 | 0.86 |
| *Ph. cinereus* | 0.53 | 0.79 | 0.53 | 0.74 | 0.63 | 0.21 | 0.05 | - | 0.79 | 0.79 | 0.47 | 0.79 | 0.95 | 0.74 | - | 0.79 | 0.79 | 0.79 | 0.95 | 1.00 | 0.79 | 0.84 |
| *V. ursinus* | 0.20 |  | 0.40 | 1.00 | 0.90 | 0.30 | 0.80 | - | 0.60 | 0.80 | 0.30 | 0.30 | 0.70 | 0.90 | - | 0.80 | 0.80 | 0.90 | - | 1.00 | 0.80 | 0.90 |
| *Pe. penicillata* | 0.13 | 0.50 | 0.19 | 0.75 | 0.25 | 0.13 | 0.13 | 0.38 | 0.25 | 0.31 | 0.19 | 0.50 | 0.75 | 0.44 | - | 0.69 | 0.81 | 0.94 | - | 1.00 | 0.94 | 1.00 |

**Supplementary Table 21.** Part three of the table showing the relative timing of all of the bone contacts.

| **Relative timing** | **pr-vo** | **pr-as** | **pr-bs** | **in-ml** | **bo-bs** | **bs-pe** | **et-sq** | **as-et** | **in-pe** | **et-pe** | **fr-mx** | **fr-pl** | **as-fr** | **as-pa** | **bo-eo** | **ip-sq** | **so-eo** | **so-sq** | **so-pe** | **ju-de** | **as-de** | **sq-de** |
| --- | --- | --- | --- | --- | --- | --- | --- | --- | --- | --- | --- | --- | --- | --- | --- | --- | --- | --- | --- | --- | --- | --- |
| *T. vulpecula* | 0.76 | 1.00 | 1.00 | 0.76 | 1.00 | - | 0.90 | 0.95 | 0.76 | 0.86 | - | 0.81 | 0.95 | - | 1.00 | 0.95 | 0.95 | 0.95 | - | - | - | - |
| *D. viverrinus* | 0.93 | 0.93 | 1.00 | 0.86 | 1.00 | 0.86 | - | 0.64 | 0.93 | 0.79 | 0.86 | 0.79 | 0.93 | - | 0.86 | - | 0.93 | - | 1.00 | 0.93 | - | 0.93 |
| *Ph. cinereus* | 0.89 | 0.95 | 1.00 | 0.79 | 0.95 | - | 0.79 | 0.89 | 0.84 | 0.95 | 0.89 | 0.89 | 0.95 | 0.89 | 1.00 | - | 1.00 | 0.95 | 1.00 | - | - | - |
| *V. ursinus* | 1.00 | 1.00 | 1.00 | 0.90 | 1.00 | - | - | - | 0.90 | - | 1.00 | 1.00 | 0.00 | - | 1.00 | - | 1.00 | - | - | - | - | - |
| *Pe. penicillata* | 1.00 | 1.00 | 1.00 | 0.81 | 1.00 | - | 0.94 | - | 0.88 | 0.69 | 0.81 | 1.00 | 0.00 | - | 1.00 | - | 1.00 | - | - | - | - | - |

**Supplementary Table 22.** List of the contacts that deviate most from the reconstructed australidelphian ancestor. Bone contacts in general occur relatively early in *D. viverrinus* and *Pe. penicillata* and relatively late in *Ph. cinereus* (See Supplementary Tables S17 to 19).

| **Species** | **Bone contact** | **Relative timing of bone contact species** | **Relative timing of bone contact ancestor** |
| --- | --- | --- | --- |
| *Phascolarctos cinereus* | premaxilla-nasal | 0.79 | 0.42 |
|  | maxilla-nasal | 0.95 | 0.63 |
|  | jugal-squamosal | 0.05 | 0.39 |
|  | interparietal-parietal | 0.9 | 0.65 |
| *Vombatus ursinus* | jugal-squamosal | 0.8 | 0.39 |
|  | lacrimal-palatine | 0.9 | 0.66 |
|  | interparietal-parietal | 0.9 | 0.65 |
| *Dasyurus viverrinus* | lacrimal-jugal | 0.86 | 0.66 |
|  | interparietal-parietal | 0.86 | 0.65 |
|  | premaxilla-palatine | 0.93 | 0.69 |
|  | supraoccipital-interparietal | 0.14 | 0.29 |
|  | frontal-nasal | 0.21 | 0.49 |
|  | alisphenoid-orbitosphenoid | 0.29 | 0.56 |
|  | maxilla-nasal | 0.29 | 0.63 |
|  | petrosal-exoccipital | 0.29 | 0.63 |
|  | basisphenoid-orbitosphenoid | 0.43 | 0.78 |
| *Trichosurus vulpecula* | suproccipital-interparietal | 0.05 | 0.29 |
| *Petrogale penicillata* | supraoccipital-interparietal | 0.63 | 0.29 |
|  | alisphenoid-basisphenoid | 1 | 0.54 |
|  | lacrimal-maxilla | 0.19 | 0.44 |
|  | squamosal-parietal | 0.25 | 0.62 |
|  | lacrimal-jugal | 0.25 | 0.66 |
|  | premaxilla-palatine | 0.25 | 0.69 |
|  | premaxilla-vomer | 0.31 | 0.78 |
